# Towards dual inhibitors of the MET kinase and WNT signaling pathway; design, synthesis and biological evaluation†

**DOI:** 10.1039/c9ra08954c

**Published:** 2019-11-13

**Authors:** Vegard Torp Lien, Margrethe Konstanse Kristiansen, Solveig Pettersen, Mads Haugland Haugen, Dag Erlend Olberg, Jo Waaler, Jo Klaveness

**Affiliations:** Department of Pharmacy, University of Oslo Oslo Norway v.t.lien@farmasi.uio.no; Department of Tumor Biology, Institute for Cancer Research, OUS Radiumhospitalet Oslo Norway; Norsk Medisinsk Syklotronsenter AS, OUS Rikshospitalet Oslo Norway; Department of Microbiology, Section for Cell Signaling, OUS Rikshospitalet Oslo Norway

## Abstract

Both the kinase MET and the WNT signaling pathway are attractive targets in cancer therapy, and synergistic effects have previously been observed in animal models upon simultaneous inhibition. A strategy towards a designed multiple ligand of MET and WNT signaling is pursued based on the two hetero biaryl systems present in both the MET inhibitor tepotinib and WNT signaling inhibitor TC-E 5001. Initial screening was conducted to find the most suitable ring systems for further optimization, whereas a second screen explored modifications towards pyridazinones and triazolo pyridazines. Up to 54% reduction of WNT signaling activity at 10 μM concentration was achieved, however, only low affinities towards MET were observed. Overall, the thiophene substituted pyridazinone 40 was the best dual MET and WNT signaling inhibitor, with a 17% and 19% reduction of activity, respectively. Although further optimizations are needed to achieve more potent dual inhibitors, the strategy presented herein can be valuable towards the development of a dual inhibitor of MET and WNT signaling.

## Abstract 2

Dual inhibitors of MET and WNT signaling may have synergistic effects, and the design, synthesis and evaluation of such first-in-class small-molecules are reported.

## Graphical abstract

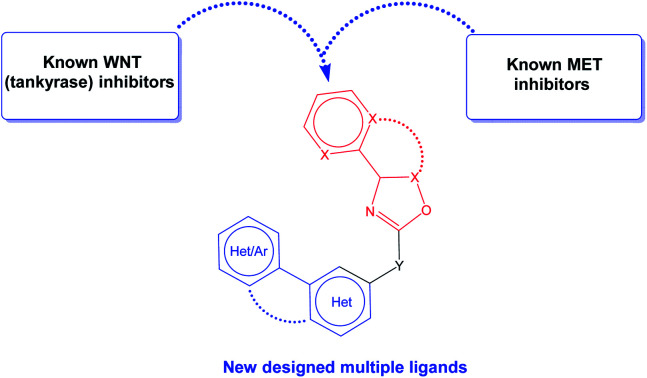

## Introduction

The tyrosine kinase MET and the WNT/β-catenin signaling pathway (WNT signaling) have both received considerable interest as targets in cancer therapy due their involvement in carcinogenesis.^1–3^ MET is dysregulated in a wide range of malignancies, and is correlated with a poor prognosis.^4^ WNT signaling is critical for cell repair and stem cell maintenance, and aberrant activity is closely associated with the development and growth of a plethora of cancerous diseases. Therefore, small-molecule WNT signaling inhibitors are predicted to be effective anti-tumor drugs in humans.^5^ Importantly, both MET and WNT signaling pathways hold a central role in the development of metastasis, and a strong biochemical connection between the two have been documented.^6,7^ Hence, simultaneous inhibition have demonstrated synergistic effects in breast cancer models,^8^ and overcoming of resistance in lung cancer^9^ and melanoma cell lines.^10^ In addition, a bispecific antibody inhibiting both MET and WNT signaling has been developed, which synergistically reduced tumor growth in a lung cancer model *in vivo*.^11^

While the prevalent strategy in drug discovery is “one drug, one target”, the realization that many drugs exerts their effect through more than one target have led to the increased interest in designed multiple ligands (DML).^12,13^ Well-designed DMLs can reduce drug–drug interactions and side effects, and simplify drug pharmacokinetics.^14,15^ In search of novel treatment strategies for cancer, we aim to develop dual inhibitors of MET and WNT signaling.

**Fig. 1:**
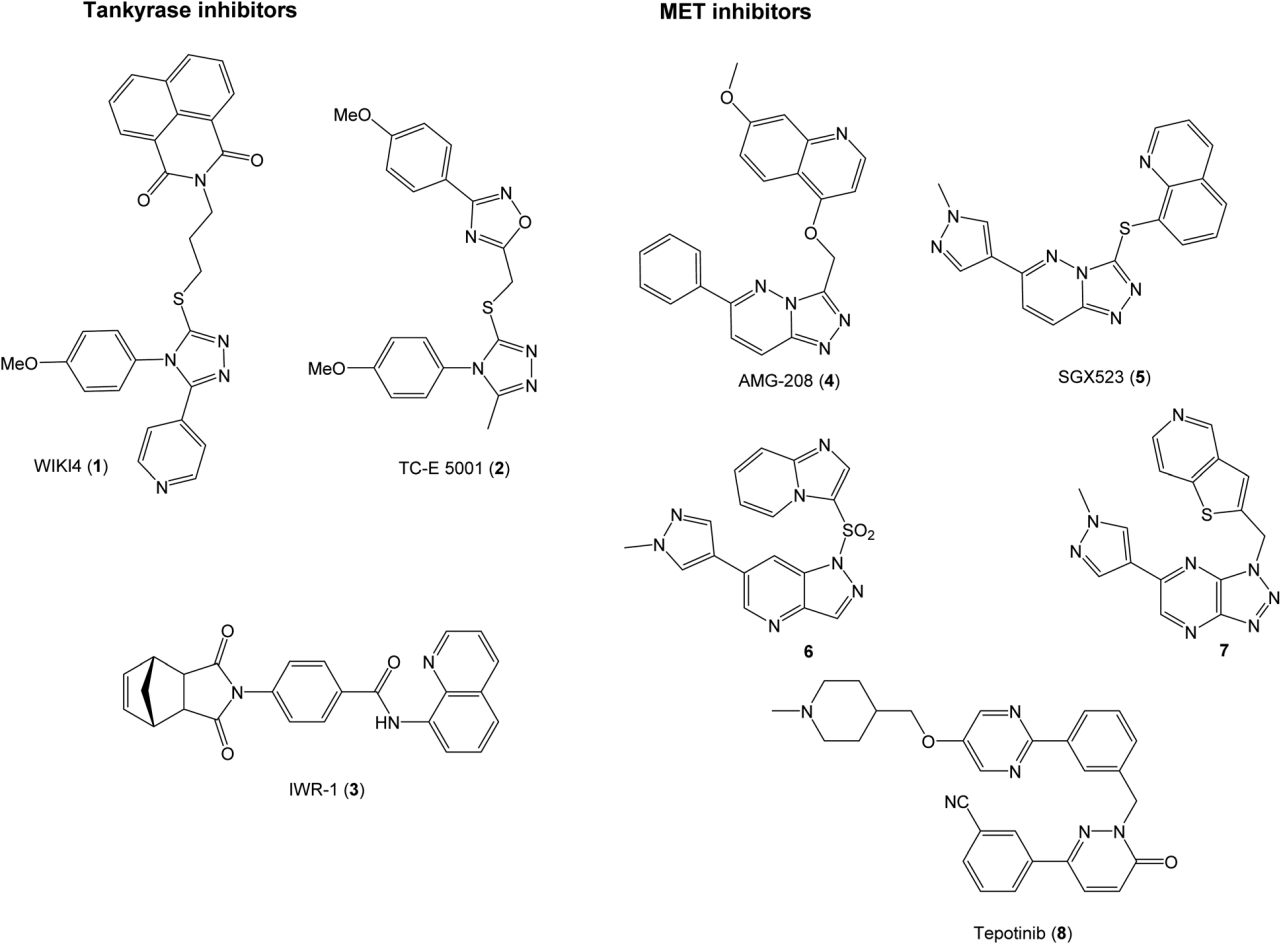
Examples of inhibitors of TNKS1/2 and MET.

The proteins tankyrase 1 and 2 (TNKS1/2) are central regulators of WNT signaling,^16^ and TNKS1/2 inhibitors are promising in the development of WNT signaling modulators. TNKS1/2 poly(ADP-ribosyl)ate AXIN proteins to earmark them for degradation by the ubiquitin–proteasomal system. Inhibition of TNKS1/2 can therefore lead to stabilization of AXIN proteins and hence the β-catenin degradosomes that in turn enhances the degradation of β-catenin.^17^ The TNKS1/2 inhibitors can attain three different binding modes in the active site. This, combined with the fact that many of the known inhibitors are discovered through high-throughput screening leads to structures spanning a wide chemical space.^18^ Some examples of TNKS1/2^19–21^ and MET inhibitors^22–26^ are shown in Fig. 1.

**Fig. 2:**
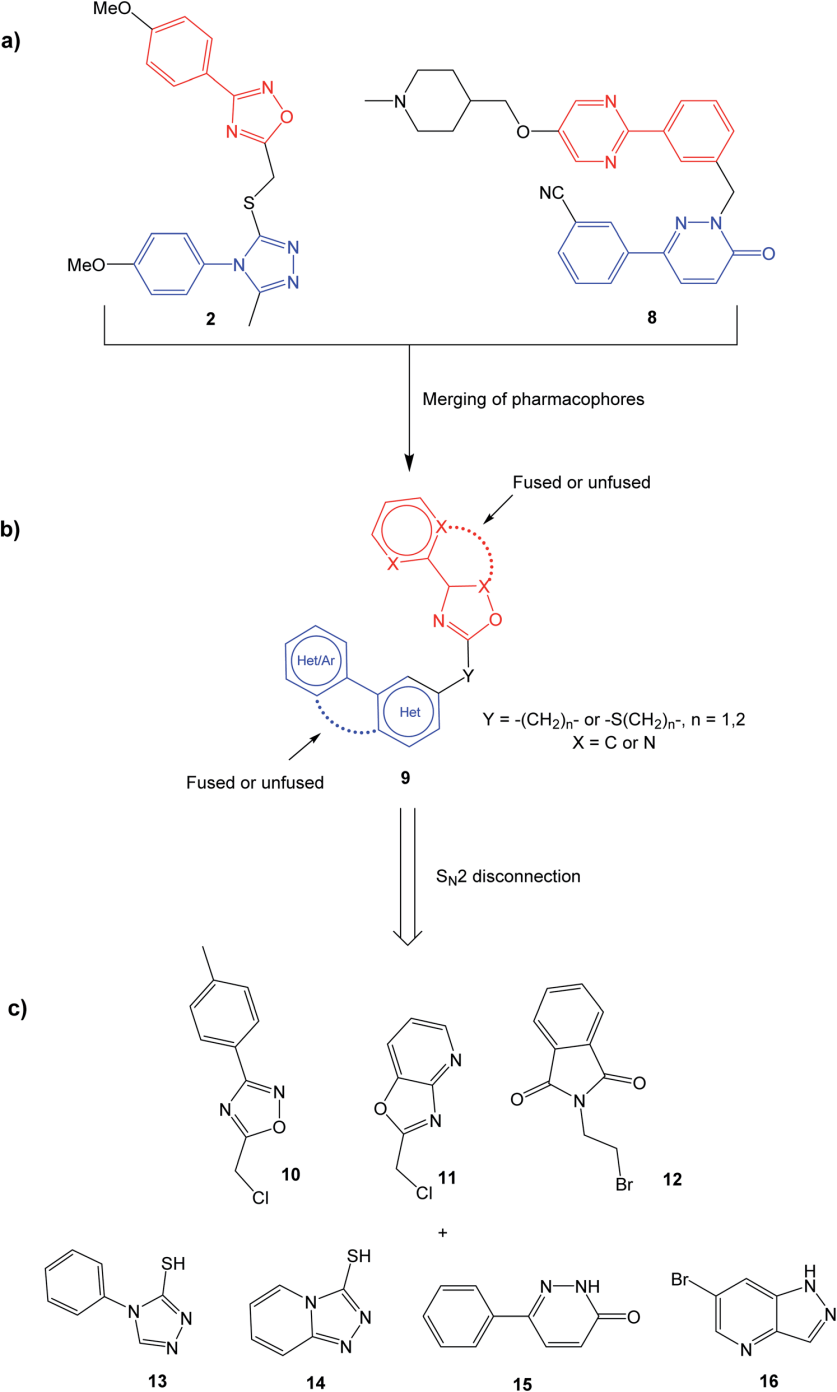
Construction of the generalized target structure 9. (a) Similarities between TC-E 5001 (2) and tepotinib (8) are highlighted; (b) merging of pharmacophores yields the target structure 9; (c) the target structure can be synthesized *via* S_N_2 reactions from commercial starting materials. See the experimental data for synthetic details.

The selective MET inhibitors have traditionally two fused heterocycles connected with a one or two atom linker, as exemplified with structures 4–7. Several different heterocyclic moieties have been explored, and the linker enables the ligands to adopt a U-shape in the active site of MET.^27^ One example of a MET inhibitor deviating from this pattern is tepotinib (8), consisting of two unfused hetero biaryl systems. As such, tepotinib creates a link from the traditional MET inhibitors to the TNKS1/2 inhibitor TC-E 5001 (2). This similarity is elaborated in Fig. 2a; two hetero biaryls, each colored in red or blue, connected by a linker make up a common structural core. For a dual MET and WNT signaling inhibitor, these two pharmacophores are merged to the target scaffold 9 in Fig. 2b. In 9, the ring systems can be either fused or unfused, and the positions of heteroatoms and substituents can be altered for structural optimization.

Initially, the aim was to evaluate which heteroaromatic ring systems that best could be applied in the target structure 9. By utilizing a S_N_2 disconnection and commercial starting materials, a selection of structural moieties from known TNKS1/2 and MET inhibitors could be combined efficiently to the target scaffold 9. In Fig. 2c, oxadiazole 10 is similar to the oxadiazole in TC-E 5001 (2), while 11 represents a fused analog of this moiety, and at the same time bearing similarities with MET inhibitors 6 and 7. Phthalimide 12 is inspired by the TNKS1/2 inhibitors WIKI4 (1) and IWR-1 (3). Triazole 13 is inspired by the triazole in TC-E 5001 (2), while the triazolopyridine 14 is the fused analog of 13, at the same time representing known MET inhibitors. The pyridazinone 15 is similar to tepotinib (8), while pyrazolo pyridine 16 is inspired by the MET inhibitor 6.

## Results and discussion

**Fig. 3:**
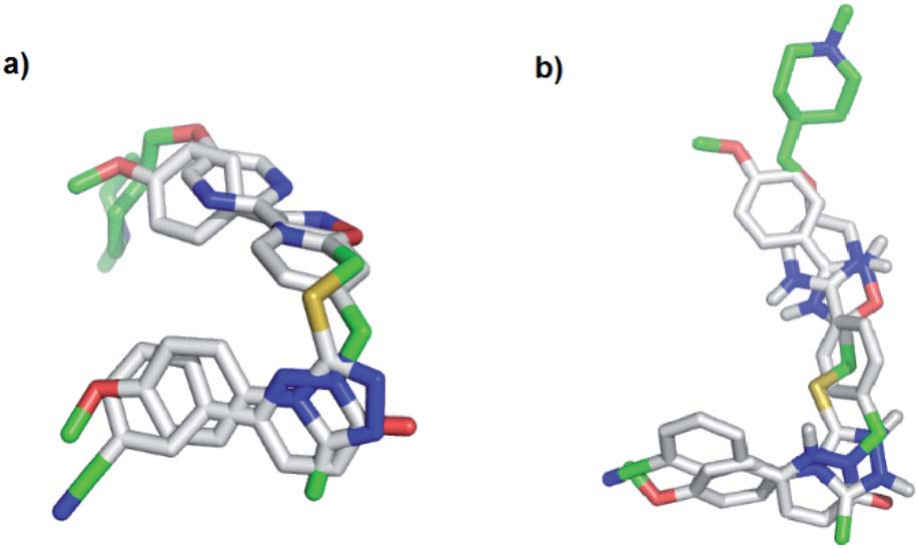
*In silico* docking of: (a) tepotinib (8) and TC-E 5001 (2) in MET; (b) tepotinib (8) and TC-E 5001 (2) in TNKS1.

To evaluate the hypothesis that known MET and TNKS1/2 inhibitors have structural similarities that can be applied in the development of dual MET and WNT signaling inhibitors, *in silico* docking was performed. Tepotinib and TC-E 5001 were docked into the TNKS1 and MET active site, respectively, and a good overlap with the experimental structures were observed in both proteins (Fig. 3). The pyridazinone in tepotinib and the triazole in TC-E 5001 replace each other in the two active sites, and the linkers enables the molecules to adopt the conformations observed in both parent crystallographic structures.

**Fig. 4:**
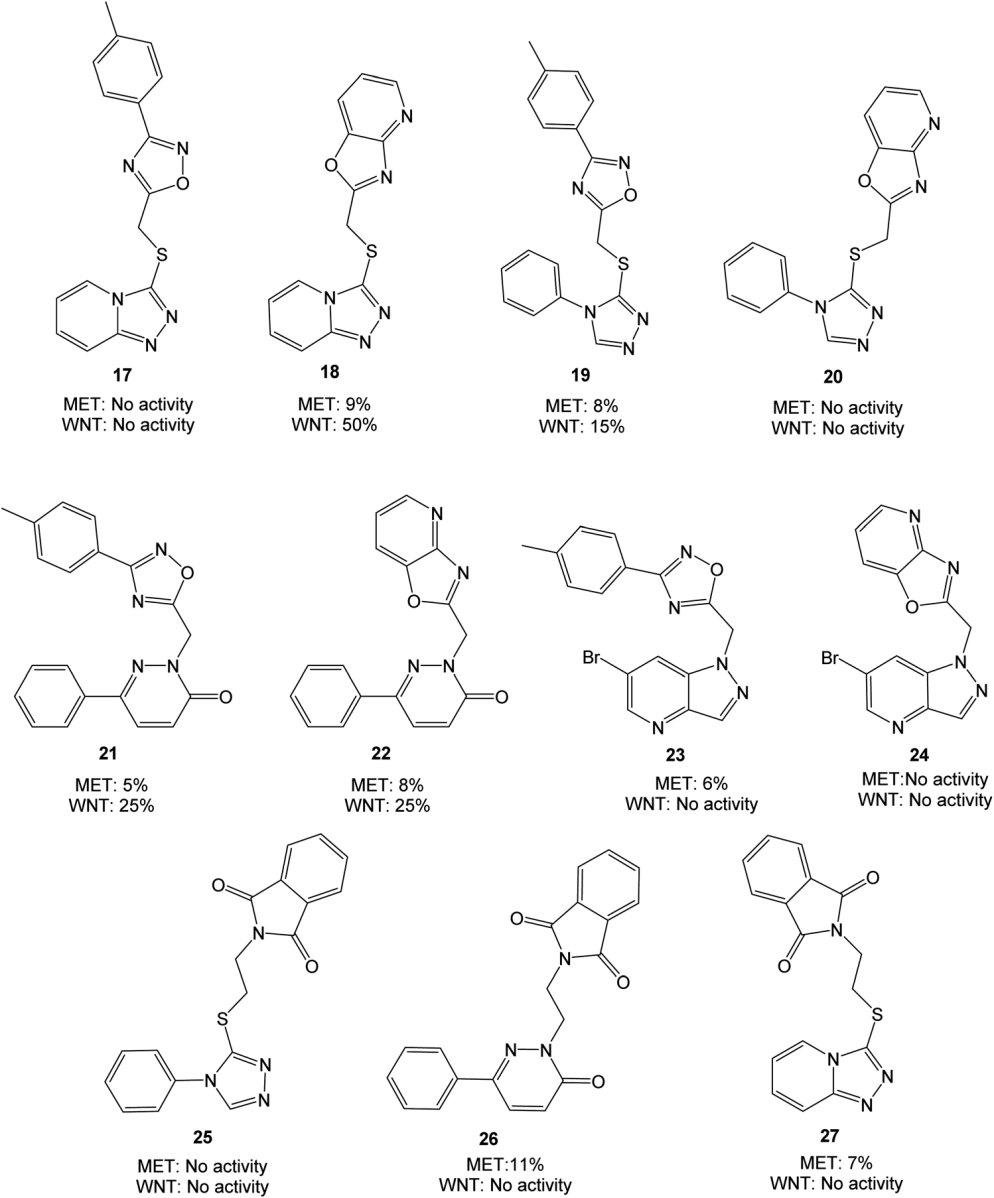
Molecules evaluated in the initial screen at 10 μM concentration, presented with values from MET and WNT signaling assays. Inhibition values are presented as averages from ≥2 parallels.

Based on the target scaffold 9 in Fig. 2b, combinations of the nucleophiles and electrophiles in Fig. 2c resulted in the molecules presented in Fig. 4, which were synthesized and evaluated in enzymatic MET (Cyclex) and cellular luciferase-based WNT/β-catenin signaling reporter assays (SuperTOPFlash assay) at 10 μM concentration.

The triazolopyridine 18 was the most potent in the WNT signaling assay with a 50% reduction of activity, while the unfused analog 17 was inactive. The pyridazinones displayed the same inhibition (25%) regardless of whether an unfused oxadiazole (21) or a fused oxazolo pyridine (22) was present. The large structural similarity between 19 and TC-E 5001 (2) and the modest inhibition of WNT signaling activity with 19 should be noted, indicating a relatively sensitive pharmacophore. MET inhibition did not reach the anticipated level for neither of the analyzed compounds.

**Scheme 1:**
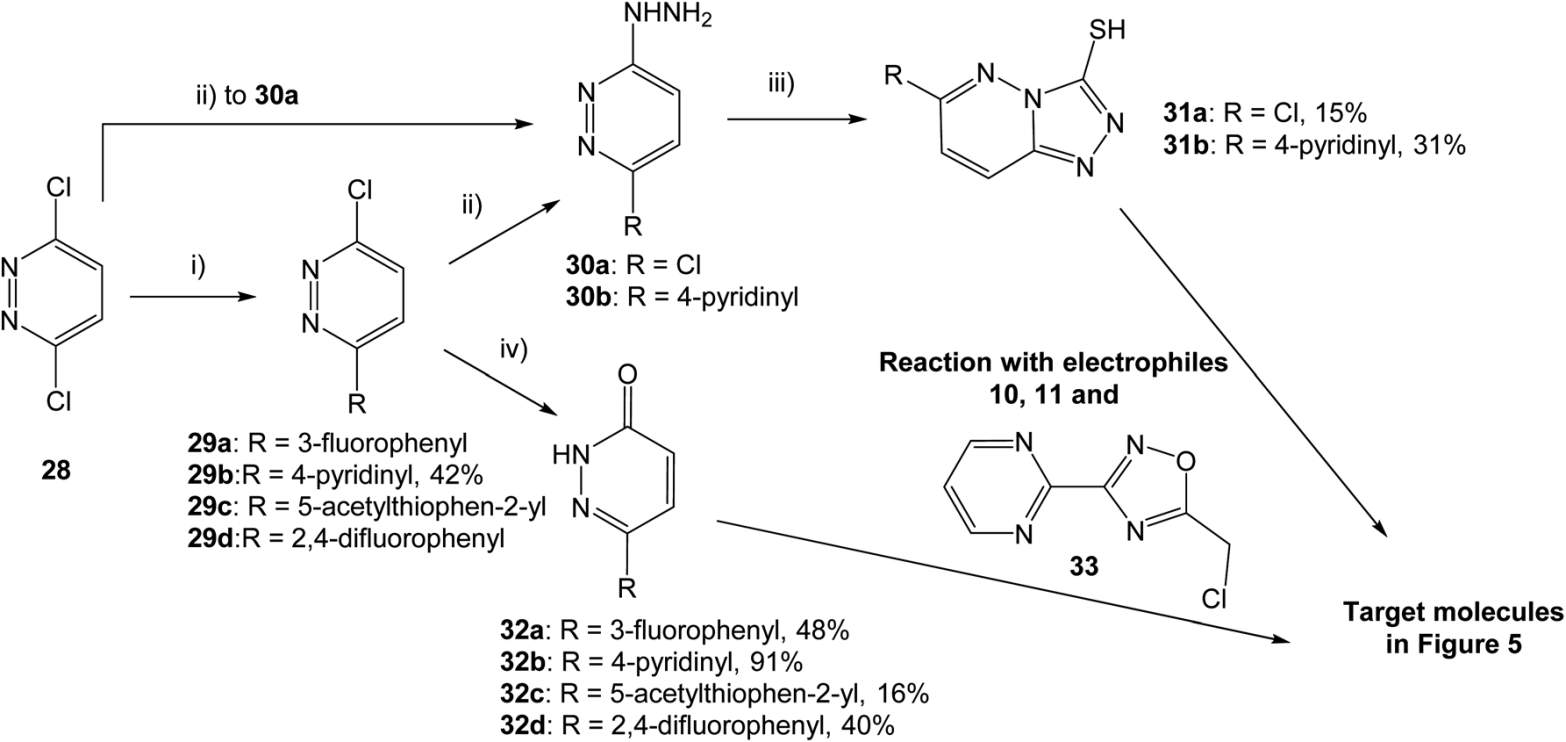
Synthesis of pyridazinone and triazolo pyridazine analogs. (i) Boronic acid, Pd(dppf)Cl_2_·CH_2_Cl_2_, K_2_CO_3_, 1,4-dioxane, H_2_O, 80 °C, 15 h; (ii) hydrazine hydrate, EtOH, 80 °C, 3–5 h; (iii) CS_2_, KOH, EtOH, rt for 48 h or 80 °C for 15 h; (iv) AcOH, 80 °C for 4 h or 100 °C for 15 h. Yields are given for isolated structures.

With two fused heterocycles, 18 shares common chemical characteristics with known MET inhibitors, and should allow for improvement of the MET affinity. The pyridazinone in 21 and 22 can also serve as a foundation for optimization, and further modifications were therefore based on 18, 21 and 22. With the syntheses shown in Scheme 1, the strategy was to interchange the triazolo pyridine in 18 to a triazolo pyridazine, and to introduce various substituents on the pyridazinone and triazolo pyridazine.

**Fig. 5:**
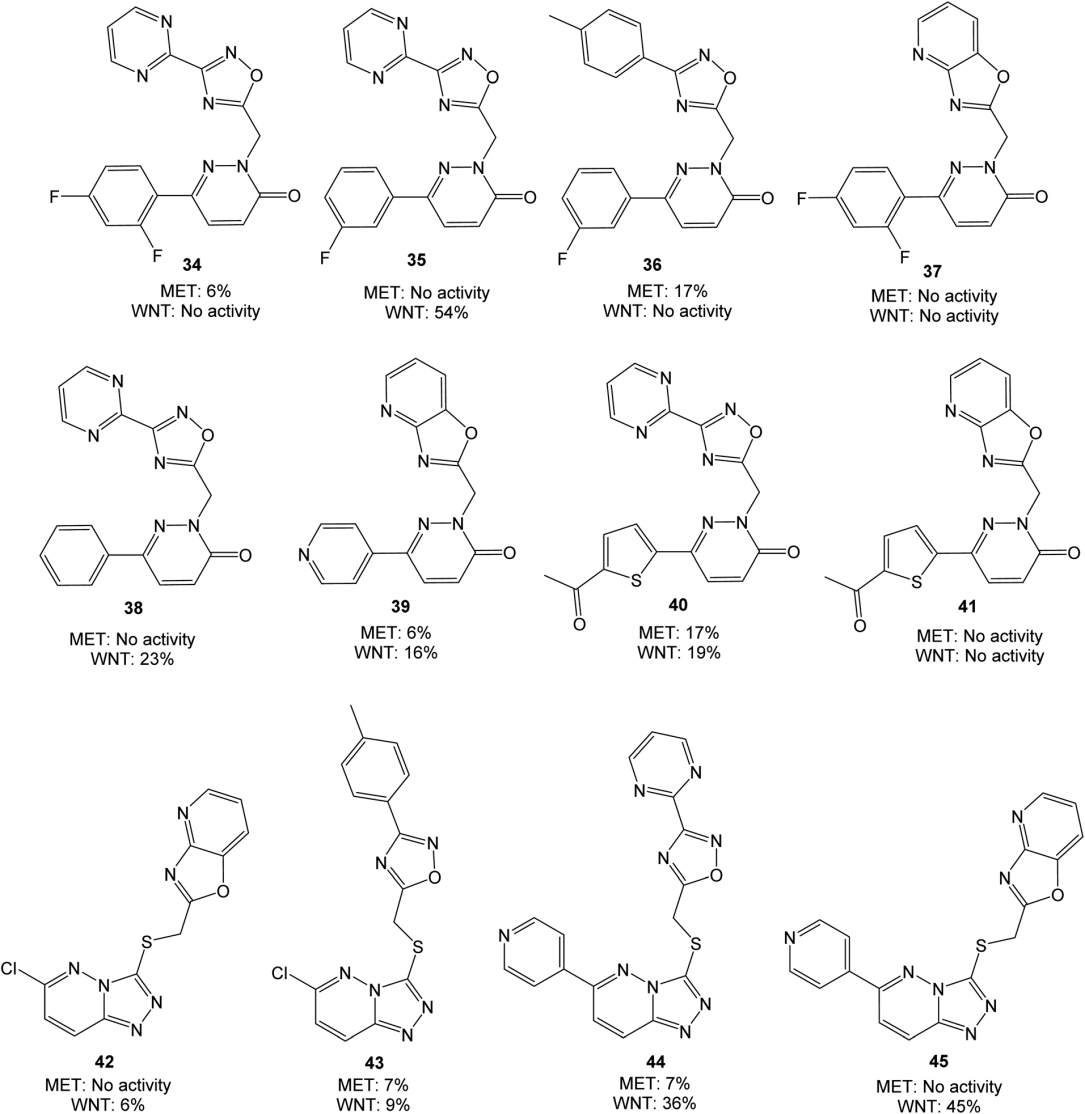
Molecules evaluated in the second screen at 10 μM concentration, presented with values from MET and WNT signaling assays. Inhibition values are presented as averages from ≥2 parallels.

Both pyridazinones and triazolo pyridazines could be synthesized starting from 3,6-dichloropyridazine (28). Suzuki–Miyaura coupling gave the intermediate chloro pyridazines 29, which could be hydrolyzed with AcOH to substituted pyridazinones 32. Nucleophilic attack on 29 by hydrazine hydrate and subsequent cyclization with CS_2_ gave the corresponding triazolo pyridazines 31. To obtain the target structures, the pyridazinones and triazolo pyridazines were reacted with electrophilic chlorides. To potentially increase the affinity for MET, the oxadiazole pyrimidine 33 was included due to reported interactions between the pyrimidine ring and the active site in the crystal structure of tepotinib in MET.^26^ The novel analogs were evaluated in MET and WNT signaling assays, and the results are presented in Fig. 5.

Overall, the performed modifications did not increase the affinity for MET. Comparing *e.g.*35 or 38 with tepotinib, the main difference is the oxadiazole in place for the phenyl group. This modification can alter the orientation of the ligand, although successful modifications have been made at this position earlier.^28,29^ Neither of the triazolo pyridazines 44 and 45 were able to inhibit MET, despite large similarities with *e.g.* AMG-208 (4). An increase from 25% to 54% in WNT signaling inhibition was obtained when moving from 21 and 22 to 35, however, low WNT signaling inhibition was observed for the remaining pyridazinones. Optimization of 18 to the structures 44 and 45 led to a similar or somewhat reduced WNT signaling inhibition. For this triazolo pyridazine series, the chlorine substituent in 42 and 43 reduced the WNT inhibition. The best combined inhibition of MET and WNT signaling, albeit modest, was achieved with the thiophene analog 40, with 17% and 19% reduction, respectively.

## Conclusion

The design of a scaffold for potential dual inhibitors of MET and WNT signaling was performed from the MET inhibitor tepotinib (8) and the tankyrase inhibitor TC-E 5001 (2). The target scaffold 9 was designed so that facile S_N_2 reactions would furnish the final products directly, facilitating screening of the most favorable structural cores. *In silico* docking revealed a potential good overlap of tepotinib and TC-E 5001 in both active sites. The first compound screen demonstrated low MET inhibition, while WNT signaling activity could be reduced by 50% at 10 μM concentration. Further structural modifications were carried out based on the triazolo pyridine 18 and pyridazinones 21 and 22. In the second compound screen, the pyridazinone 35 inhibited WNT signaling by 54%, however with no inhibition of MET. The thiophene substituted pyridazinone 40 was overall the best dual MET and WNT signaling inhibitor, with a 17% and 19% reduction of activity, respectively. Although further structural optimizations are needed, the novel strategy provided herein could prove valuable for further development of dual MET and WNT signaling inhibitors.

## Experimental

### General

All chemicals were purchased from Sigma-Aldrich or Fluorochem and used without further purification. Air and/or moisture sensitive reactions were performed under argon atmosphere with dried solvents and reagents. TLC was performed on Merck silica gel 60 F_254_ plates, and visualized using UV light at 312 nm or 365 nm, a phosphomolybdic acid solution (12 g phosphomolybdic acid in 250 mL EtOH) or a potassium permanganate (1.5 g KMnO_4_, 10 g K_2_CO_3_, 2.5 mL 5 M NaOH/H_2_O, 200 mL H_2_O) solution for detection. Column chromatography was performed with silica gel (pore size 60 Å, 230–400 mesh particle size). ^1^H and ^13^C NMR spectra were obtained on a Bruker AVIII HD 400 instrument (400/101 MHz) or Bruker AVII 600 (600/151 MHz). Chemical shifts (*δ*) are reported in parts per million (ppm), and coupling constants are reported in hertz (Hz). The residual proton solvent resonance in ^1^H NMR (CDCl_3_ at *δ* 7.27, DMSO-*d*_6_ at *δ* 2.50) and the residual carbon solvent resonance in ^13^C NMR (CDCl_3_ at *δ* 77.16 ppm and DMSO-*d*_6_ at *δ* 39.52) are used as reference. Accurate mass determination (HRMS) in positive or negative mode was performed on a Waters Prospec Q instrument, ionized by electrospray (ESI). LC-MS was performed on a Thermo Finnigan LCQ Deca XP Plus with a gradient from 10% to 90% acetonitrile in water over 10 minutes.

### General procedure for the alkylations towards the biologically tested compounds

The nucleophile and Cs_2_CO_3_ were dissolved in DMF (2 mL). After 5 minutes, the electrophile dissolved in DMF (1 mL) was added dropwise, and the reaction was stirred at ambient temperature for 15 hours (70 °C where noted). Work-up and purification: method A; The reaction mixture was diluted with EtOAc (2 mL) and water (2 mL), and the organic phase was separated. The aqueous phase was extracted with EtOAc (2 × 5 mL), and the combined organic phases were washed with water (4 × 5 mL) and brine (5 mL), dried over MgSO_4_ and reduced on a rotary evaporator. The crude product was purified by column chromatography (Hep : EtOAc (1 : 1) → Hep : EtOAc : MeOH (10 : 10 : 2)). Method B: The reaction mixture was reduced on a rotary evaporator, and purified by column chromatography (2–5% MeOH in DCM).

#### 5-(([1,2,4]Triazolo[4,3-*a*]pyridin-3-ylthio)methyl)-3-(*p*-tolyl)-1,2,4-oxadiazole (17)

Prepared according to the general procedure by reacting [1,2,4]triazolo[4,3-*a*]pyridine-3-thiol (14) (40 mg, 0.265 mmol) and 5-(chloromethyl)-3-(*p*-tolyl)-1,2,4-oxadiazole (10) (71 mg, 0.340 mmol) in the presence of Cs_2_CO_3_ (85 mg, 0.261 mmol). Work-up and purification were performed according to method A and the title compound was obtained as a white solid (58 mg, 67%). ^1^H NMR (400 MHz, CDCl_3_): *δ* 8.10 (d, 1H, *J* = 6.9 Hz), 7.81 (d, 1H, *J* = 9.2 Hz), 7.73 (d, 2H, *J* = 8.2 Hz), 7-32-7-28 (m, 1H), 7.22 (d, 2H, *J* = 8.0), 6.85 (t, 1H, *J* = 6.7 Hz), 4.45 (s, 2H), 2.39 (s, 3H). ^13^C NMR (101 MHz, CDCl_3_) *δ* 175.27, 168.77, 151.60, 141.95, 138.64, 129.63, 128.18, 127.36, 123.35, 123.23, 116.69, 114.61, 30.28, 21.67. HRMS (ESI+) *m*/*z* calcd for C_16_H_13_N_5_NaOS [MNa]^+^: 346.0733, found 346.0733.

### Synthesis of intermediates

#### General procedure for Suzuki–Miyaura reactions

3,6-Dichloropyridazine, boronic acid and K_2_CO_3_ were put under argon atmosphere. 1,4-dioxane and H_2_O were added using syringes, and the solution was degassed with argon for 15 minutes. Pd(dppf)Cl_2_·CH_2_Cl_2_ was added, the solution degassed for 5 minutes and the reaction tube was sealed. The reaction mixture was stirred at 80 °C for 15 hours and reduced on a rotary evaporator. AcOH was added and the mixture was stirred at 80 °C for 4 hours (except 32b). The reaction mixture was reduced on a rotary evaporator and the crude product was purified by column chromatography (5% MeOH in DCM).

##### 3-Chloro-6-(pyridin-4-yl)pyridazine (29b)

3,6-Dichloropyridazine (28) (686 mg, 4.6 mmol), 4-pyridinyl boronic acid (531 mg, 4.3 mmol), K_2_CO_3_ (565 mg, 4.1 mmol) and Pd(dppf)Cl_2_·CH_2_Cl_2_ (170 mg, 0.208 mmol) in 1,4-dioxane (6 mL) and H_2_0 (2 mL) were reacted according to the general protocol. The reaction mixture was reduced on a rotary evaporator and the crude product was purified by column chromatography (5% MeOH in DCM) and the title compound was obtained as a grey solid (349 mg, 42%). The spectroscopic analysis was in agreement with earlier reported data.^28^^1^H NMR (400 MHz, CDCl_3_) *δ* 8.83 (d, 2H, *J* = 5.5 Hz), 7.98 (d, 2H, *J* = 5.7 Hz), 7.90 (d, 1H, *J* = 8.9 Hz), 7.66 (d, 1H, *J* = 8.9 Hz).

##### 6-(3-Fluorophenyl)pyridazin-3(2*H*)-one (32a)

3,6-Dichloropyridazine (28) (249 mg, 1.68 mmol), (3-fluorophenyl)boronic acid 24 (286 mg, 2.04 mmol), K_2_CO_3_ (460 mg, 3.32 mmol) and Pd(dppf)Cl_2_·CH_2_Cl_2_ (136 mg, 0.167 mmol) in 1,4-dioxane (10 mL) and H_2_O (3 mL) were reacted according to the general protocol. The intermediate 3-chloro-6-(3-fluorophenyl)pyridazine (29a) was hydrolyzed in AcOH (3 mL). The title compound was obtained as a light grey solid (153 mg, 48%). The spectroscopic analysis was in agreement with earlier reported data.^30^^1^H NMR (400 MHz, DMSO-*d*_6_) *δ* 13.27 (s, 1H), 8.06 (d, *J* = 9.93 Hz, 1H), 7.71 (d, *J* = 8.02 Hz, 1H), 7.67 (dt, *J* = 10.48, 2.08 Hz, 1H), 7.52 (td, *J* = 8.16 Hz, 6.17 Hz, 1H), 7.27 (td, *J* = 8.52, 2.66 Hz, 1H), 7.00 (d, *J* = 9.94 Hz, 1H). ^13^C NMR (400 MHz, DMSO-*d*_6_) *δ* 163.1 (d, *J* = 243.5 Hz), 160.7, 143.1 (d, *J* = 2.94 Hz), 137.5 (d, *J* = 7.98 Hz), 131.9, 131.5 (d, *J* = 8.38 Hz), 130.6, 122.2 (d, *J* = 2.74 Hz), 116.4 (d, *J* = 20.83 Hz), 112.8 (d, *J* = 23.31 Hz).

##### 6-(Pyridin-4-yl)pyridazin-3(2*H*)-one (32b)

AcOH (3 mL) was added to 3-chloro-6-(pyridin-4-yl)pyridazine (29b) (100 mg, 0.52 mmol) and stirred at 100 °C for 15 hours. The reaction mixture was reduced on a rotary evaporator, and the title compound was obtained as a grey solid (82 mg, 91%), and used in the next steps without further purification. The spectroscopic analysis was in agreement with earlier reported data.^28^^1^H NMR (400 MHz, DMSO-*d*_6_) *δ* 13.81 (bs, 1H), 8.94 (d, 2H, *J* = 6.5 Hz), 8.37 (d, 2H, *J* = 6.5 Hz), 8.28 (d, 1H, *J* = 9.9 Hz), 7.12 (d, 1H, *J* = 9.9 Hz).

##### 6-(5-Acetylthiophen-2-yl)pyridazin-3(2*H*)-one (32c)

3,6-Dichloropyridazine (28) (301 mg 2.02 mmol), (5-acetylthiophen-2-yl)boronic acid (339 mg, 1.99 mmol), K_2_CO_3_ (592 mg, 4.28 mmol) and Pd(dppf)Cl_2_·CH_2_Cl_2_ (164 mg, 0.201 mmol) in 1,4-dioxane (10 mL) and H_2_O (3 mL) were reacted according to the general protocol. The intermediate 1-(5-(6-chloropyridazin-3-yl)thiophen-2-yl)ethan-1-one (29c) was hydrolyzed in AcOH (10 mL). The title compound was obtained as a brown solid (69 mg, 16%). ^1^H NMR (400 MHz, DMSO-*d*_6_) *δ* 13.26 (s, 1H), 8.09 (d, *J* = 9.86 Hz, 1H), 7.95 (d, *J* = 3.82 Hz, 1H), 7.78 (d, *J* = 3.69 Hz, 1H), 7.02 (d, *J* = 10.2 Hz, 1H), 2.54 (s, 3H). ^13^C NMR (400 MHz, DMSO-*d*_6_) *δ* 191.5, 160.5, 146.8, 144.8, 140.3, 135.1, 131.0, 130.7, 127.8, 26.95. HRMS (ESI+) *m*/*z* calcd for C_10_H_8_N_2_O_2_S [MNa]^+^: 243.0199, found 243.0198.

##### 6-(2,4-Difluorophenyl)pyridazin-3(2*H*)-one (32d)

3,6-Dichloropyridazine (28) (54 mg, 0.36 mmol), 2,4-difluorophenyl boronic acid (68 mg, 0.43 mmol), K_2_CO_3_ (99 mg, 0.72 mmol) and Pd(dppf)Cl_2_·CH_2_Cl_2_ (24 mg, 0.029 mmol) in 1,4-dioxane (2 mL) and H_2_O (0.5 mL) were reacted according to the general protocol. The intermediate 3-chloro-6-(2,4-difluorophenyl)pyridazine (29d) was hydrolyzed in AcOH (3 mL). The title compound was obtained as a light grey solid (30 mg, 40%). ^1^H NMR (600 MHz, DMSO-*d*_6_) *δ* 13.35 (s, 1H), 7.76–7.70 (m, 2H), 7.42 (ddd, 1H, *J* = 11.3 Hz, 9.0 Hz, 2.5 Hz), 7.23 (td, 2H, *J* = 8.5, 2.4 Hz), 6.99 (d, 1H, *J* = 9.90 Hz). ^13^C NMR (151 MHz, DMSO-*d*_6_) *δ* 162.7 (dd, *J* = 249.3, 12.4 Hz), 160.0, 159.6 (dd, *J* = 251.2, 12.6 Hz), 140.3 (d, *J* = 1.7 Hz), 133.8 (d, *J* = 5.7 Hz), 131.3 (dd, *J* = 10.0, 4.2 Hz), 129.8, 119.9 (dd, *J* = 12.3, 3.7 Hz), 112.3 (dd, *J* = 21.5, 3.5 Hz), 104.8 (t, *J* = 26.2 Hz). HRMS (ESI+) *m*/*z* calcd for C_10_H_6_F_2_N_2_NaO [M + H]^+^: 231.0340, found 231.0340.

##### 6-Chloro-[1,2,4]triazolo[4,3-*b*]pyridazine-3-thiol (31a)

3,6-Dichloropyridazine (28) (214 mg, 1.44 mmol) was suspended in EtOH (3 mL) and added hydrazine hydrate (74 μL, 1.53 mmol). The resultant mixture was heated to reflux for 3 hours and reduced on a rotary evaporator. The intermediate 3-chloro-6-hydrazinylpyridazine (30a) (36 mg, 0.249 mmol) was suspended in EtOH (1.5 mL) and added a solution of KOH (14 mg, 0.249 mmol) in H_2_O (1.5 mL). CS_2_ (30 μL, 0.496 mmol) was added and the mixture was stirred at ambient temperature for 48 h, with similar addition of KOH and CS_2_. The reaction mixture was reduced on a rotary evaporator and purified by column chromatography (5–10% MeOH in DCM). The title compound was obtained as a yellow solid (7 mg, 15% over two steps). The spectroscopic analysis was in agreement with earlier reported data.^31^^1^H NMR (600 MHz, DMSO-*d*_6_) *δ* 14.92 (s, 1H), 8.24 (d, 2H, *J* = 9.77 Hz), 7.48 (d, 2H, *J* = 9.77 Hz). ^13^C NMR (151 MHz, DMSO-*d*_6_) *δ* 160.47, 148.74, 140.16, 126.67, 124.75. HRMS (ESI−) *m*/*z* calcd for C_5_H_2_ClN_4_S [M − H]^−^: 184.9694, found 184.9694.

##### 6-(Pyridin-4-yl)-[1,2,4]triazolo[4,3-*b*]pyridazine-3-thiol (31b)

3-Chloro-6-(pyridin-4-yl)pyridazine (29b) (250 mg, 1.30 mmol) was suspended in EtOH (4 mL) and added hydrazine hydrate (150 μL, 3.1 mmol). The resultant mixture was heated to reflux for 5 hours in a sealed tube and reduced on a rotary evaporator. The intermediate 3-hydrazinyl-6-(pyridin-4-yl)pyridazine (30b) was suspended in EtOH (5 mL) and added KOH (90 mg, 1.61 mmol). The reaction mixture was placed under argon and degassed for 5 minutes. CS_2_ (30 μL, 0.496 mmol) was added and the mixture was stirred in a closed vial at 80 °C for 15 h. The reaction mixture was reduced on a rotary evaporator and purified by column chromatography (5–10% MeOH in DCM). The title compound was achieved as an orange solid (92 mg, 31% over two steps). ^1^H NMR (400 MHz, DMSO-*d*_6_) *δ* 14.89 (bs, 1H), 8.82 (m, 2H), 8.32 (d, 1H, *J* = 10.0 Hz), 8.06 (m, 2H), 8.01 (d, 1H, *J* = 10.90 Hz). ^13^C NMR (101 MHz, DMSO-*d*_6_) *δ* 151.38, 150.67, 141.71, 141.06, 126.16, 121.96, 121.08. HRMS (ESI+) *m*/*z* calcd for C_10_H_8_N_5_S [M + H]^+^: 230.0495, found 230.0495.

### *In silico* docking

Dockings were performed using Autodock Vina^32^*via* the PyRX^33^ interface. The experimental crystal structures of tepotinib in MET and TC-E 5001 in tankyrase were downloaded from the Protein Data Bank (PDB: 4R1V and 3UDD), and prepared for docking using Autodock Tools (ligand and water removed, and polar hydrogens added). The ligands were build using Avogadro,^34^ and initial geometrical optimization was done using the same software. After docking, visualization of the conformations and binding interactions were done in PyMol (The PyMOL Molecular Graphics System, Version 1.8 Schrödinger, LLC). Initially, redocking of tepotinib and TC-E 5001 into the active site of MET and tankyrase, respectively, showed to reproduce the experimental results with good accuracy.

### Biological assays

#### MET assay

The enzymatic MET assay was purchased from Cyclex and used following the manufacturer's instructions. Briefly, while placed on ice, recombinant MET was added to wells precoated with substrate, and the reaction was started by adding buffer containing the inhibitors in appropriate dilutions. The plate was incubated at 30 °C for 60 minutes. After washing with buffer, a HRP conjugated detection antibody PY-39 was added to each well, and incubated at ambient temperature for 60 minutes. The TMB substrate was added after another round of washing, and incubated at ambient temperature for 10 minutes. Stop solution was added, and absorbance was measured using a spectrophotometric plate reader (PerkinElmer VICTOR™ X3).

#### WNT/β-catenin signaling assay (SuperTOPFlash assay)

The cellular luciferase-based WNT/β-catenin signaling reporter was performed using various compound concentrations and incubated for 24 hours as previously described.^35^

## Conflicts of interest

There are no conflicts to declare.

## Supplementary Material

RA-009-C9RA08954C-s001
