# Correction: Towards dual inhibitors of the MET kinase and WNT signaling pathway; design, synthesis and biological evaluation

**DOI:** 10.1039/d0ra90007a

**Published:** 2020-01-31

**Authors:** Vegard Torp Lien, Margrethe Konstanse Kristiansen, Solveig Pettersen, Mads Haugland Haugen, Stefan Krauss, Dag Erlend Olberg, Jo Waaler, Jo Klaveness

**Affiliations:** Department of Pharmacy, University of Oslo Oslo Norway v.t.lien@farmasi.uio.no; Department of Tumor Biology, Institute for Cancer Research, OUS Radiumhospitalet Oslo Norway; Hybrid Technology Hub - Centre of Excellence, Institute of Basic Medical Sciences, University of Oslo Oslo Norway; Department of Immunology and Transfusion Medicine, Oslo University Hospital Oslo Norway; Norsk Medisinsk Syklotronsenter AS, OUS Rikshospitalet Oslo Norway

## Abstract

Correction for ‘Towards dual inhibitors of the MET kinase and WNT signaling pathway; design, synthesis and biological evaluation’ by Vegard Torp Lien *et al.*, *RSC Adv.*, 2019, **9**, 37092–37100.

The authors regret that one of the affiliations (affiliation *d*) was incorrectly shown in the original manuscript. In addition, the authors regret the omission of one of the authors, Stefan Krauss, from the original manuscript. The corrected author list and affiliations are as shown above.

In addition, the authors regret the omission of the following funding acknowledgments in the original article: MHH was supported by South-Eastern Norway Regional Health Authority (grant No. 2019081) and SP from the Norwegian Cancer Society (grant No. 190257-2017). J. W. and S. K. were supported by the Research Council of Norway (grant No. 262613, 267639), by South-Eastern Norway Regional Health Authority (grant No. 16/00528-9, 15/00779-2, 2015012 and 2019090) and from the Norwegian Cancer Society (grant No. 5803958).

The Royal Society of Chemistry apologises for these errors and any consequent inconvenience to authors and readers.

## Supplementary Material

