# Supplementary material for: Towards dual inhibitors of the MET kinase and WNT signaling pathway; design, synthesis and biological evaluation
Source: RSC Adv. 2019 Nov 13;9(63):37092–100. doi: 10.1039/c9ra08954c (PMC9075752; doi:10.1039/c9ra08954c)
Supplement: RA-009-C9RA08954C-s001 [file RA-009-C9RA08954C-s001.pdf]

Electronic Supplementary Material (ESI) for RSC Advances.
This journal is © The Royal Society of Chemistry 2019

Supporting information for

Towards dual inhibitors of the MET kinase and WNT signaling pathway; design, synthesis and biological evaluation.

Vegard Torp Lien,[a] Margrethe Konstanse Kristiansen,[a] Solveig Pettersen,[b] Mads Haugland Haugen,[b] Dag Erlend Olberg,[a,c] Jo Waaler[d] and Jo Klaveness.[a]

[a.] Department of Pharmacy, University of Oslo, Oslo, Norway

[b.] Department of Tumor Biology, Institute for Cancer Research, OUS Radiumhospitalet, Oslo, Norway

[c.] Norsk Medisinsk Syklotronsenter AS, OUS Rikshospitalet, Oslo, Norway

[d.] Department of Microbiology, Section for Cell Signaling OUS Rikshospitalet, Oslo, Norway

Content:

Experimental and analytical data not given in the main article (page 2-8).

^{1}H NMR and ^{13}C NMR spectra for all novel compounds (page 9-35).

Experimental and analytical data not given in the main article

General

All chemicals were purchased from Sigma–Aldrich or Fluorochem and used without further purification. Air and/or moisture sensitive reactions were performed under argon atmosphere with dried solvents and reagents. TLC was performed on Merck silica gel 60 F_{254} plates, and visualized using UV light at 312 nm or 365 nm, a phosphomolybdic acid solution (12 g phosphomolybdic acid in 250 mL EtOH) or a potassium permanganate (1,5 g $KMnO_4$, 10 g K_2CO_3, 2,5 mL 5M NaOH/H_2O, 200 mL H_2O) solution for detection. Column chromatography was performed with silica gel (pore size 60 Å, 230–400 mesh particle size). ^{1}H and ^{13}C NMR spectra were obtained on a Bruker AVIII HD 400 instrument (400/101 MHz) or Bruker AVII 600 (600/151 MHz). Chemical shifts (δ) are reported in parts per million (ppm), and coupling constants are reported in Hertz (Hz). The residual proton solvent resonance in ^{1}H NMR ($CDCl_3$ at δ 7.27, DMSO-d_6 at δ 2.50) and the residual carbon solvent resonance in ^{13}C NMR ($CDCl_3$ at δ 77.16 ppm and DMSO-d_6 at δ 39.52) are used as reference. Accurate mass determination (HRMS) in positive or negative mode was performed on a Waters Prospec Q instrument, ionized by electrospray (ESI). LC-MS was performed on a Thermo Finnigan LCQ Deca XP Plus using a gradient from 10% to 90% acetonitrile in water over 10 minutes.

General procedure for the alkylations towards the biologically tested compounds

The nucleophile and Cs_2CO_3 were dissolved in DMF (2 mL). After 5 minutes, the electrophile dissolved in DMF (1 mL) was added dropwise, and the reaction was stirred at ambient temperature for 15 hours (70 °C where noted). Work-up and purification: Method A; The reaction mixture was diluted with EtOAc (2 mL) and water (2 mL), and the organic phase was separated. The aqueous phase was extracted with EtOAc (2x5 mL), and the combined organic phases were washed with water (4x5 mL) and brine (5 mL), dried over $MgSO_4$ and reduced on a rotary evaporator. The crude product was purified by column chromatography (Hep:EtOAc (1:1) → Hep:EtOAc:MeOH (10:10:2). Method B: The reaction mixture was reduced on a rotary evaporator, and purified by column chromatography (2-5% MeOH in DCM).

5-(([1,2,4]Triazolo[4,3-a]pyridin-3-ylthio)methyl)-3-(p-tolyl)-1,2,4-oxadiazole (**17**)

Prepared according to the general procedure by reacting [1,2,4]triazolo[4,3-a]pyridine-3-thiol (**14**) (40 mg, 0.265 mmol) and 5-(chloromethyl)-3-(p-tolyl)-1,2,4-oxadiazole (**10**) (71 mg, 0.340 mmol) in the presence of Cs_2CO_3 (85 mg, 0.261 mmol). Work-up and purification were performed according to method A and the title compound was obtained as a white solid (58 mg, 67%). ^{1}H NMR (400 MHz, $CDCl_3$): δ 8.10 (d, 1H, J = 6.9 Hz), 7.81 (d, 1H, J = 9.2 Hz), 7.73 (d, 2H, J = 8.2 Hz), 7-32-7-28 (m, 1H), 7.22 (d, 2H, J = 8.0), 6.85 (t, 1H, J = 6.7 Hz), 4.45 (s, 2H), 2.39 (s, 3H). ^{13}C NMR (101 MHz, $CDCl_3$) δ 175.27, 168.77, 151.60, 141.95, 138.64, 129.63, 128.18, 127.36, 123.35, 123.23, 116.69, 114.61, 30.28, 21.67. HRMS (ESI+) m/z calcd. for $C_{16}H_{13}N_5NaOS$ [MNa]+: 346.0733, found 346.0733.

2-(([1,2,4]Triazolo[4,3-a]pyridin-3-ylthio)methyl)-7,7a-dihydrooxazolo[4,5-b]pyridine (**18**)

Prepared according to the general procedure by reacting [1,2,4]triazolo[4,3-a]pyridine-3-thiol (**14**) (42 mg, 0.278 mmol) and 2-(chloromethyl)oxazolo[4,5-b]pyridine (**11**) (62 mg, 0.368 mmol) in the presence of Cs_2CO_3 (139 mg, 0.427 mmol). Work-up and purification were performed according to method A and the title compound was obtained as a white solid (52 mg, 66%). ^{1}H NMR (400 MHz, $CDCl_3$) δ 8.54

(dd, *J* = 4.9, 1.5 Hz, 1H), 8.20 (d, *J* = 7.0 Hz, 1H), 7.85 (d, *J* = 9.3 Hz, 1H), 7.70 (dd, *J* = 8.2, 1.5 Hz, 1H), 7.37 (ddd, *J* = 9.3, 6.6, 1.2 Hz, 1H), 7.28 (dd, *J* = 8.2, 4.9 Hz, 1H), 6.91 (t, *J* = 6.8 Hz, 1H), 4.57 (s, 2H). ^{13}C NMR (101 MHz, $CDCl_3$) δ 165.13, 155.13, 151.12, 147.01, 143.38, 139.06, 128.72, 123.36, 120.76, 118.64, 116.47, 114.83, 76.84, 32.43. HRMS (ESI+) m/z calcd. for $C_{13}H_9N_5NaOS$ [MNa]+: 306.0420, found 306.0420

5-(((4-Phenyl-4H-1,2,4-triazol-3-yl)thio)methyl)-3-(p-tolyl)-1,2,4-oxadiazole (**19**)
Prepared according to the general procedure by reacting 4-phenyl-4H-1,2,4-triazole-3-thiol (**13**) (28 mg, 0.158 mmol) and 5-(chloromethyl)-3-(p-tolyl)-1,2,4-oxadiazole (**10**) (42 mg, 0.201 mmol) in the presence of Cs_2CO_3 (85 mg, 0.261 mmol). Work-up and purification were performed according to method A and the title compound was obtained as a white solid (45 mg, 82%). ^{1}H NMR (400 MHz, $CDCl_3$): δ 8.41 (s, 1H), 7.91 (d, 1H, *J* = 8.1 Hz), 7.54-7.50 (m, 3H), 7.37-7.35 (m, 2H), 7.28-7.26 (m, 2H), 4.74 (s, 2H), 2.41 (s, 3H). ^{13}C NMR (101 MHz, $CDCl_3$) δ 174.86, 168.78, 148.78, 145.03, 141.87, 133.03, 130.20, 130.10, 129.69, 127.53, 125.29, 123.61, 27.53, 21.72. HRMS (ESI+) m/z calcd. for $C_{18}H_{15}N_5NaOS$ [MNa]+: 372.0890, found 372.0889.

2-(((4-Phenyl-4H-1,2,4-triazol-3-yl)thio)methyl)oxazolo[4,5-b]pyridine (**20**)
Prepared according to the general procedure by reacting 4-phenyl-4H-1,2,4-triazole-3-thiol (**13**) (33 mg, 0.186 mmol) and 2-(chloromethyl)oxazolo[4,5-b]pyridine (**11**) (45 mg, 0.267 mmol) in the presence of Cs_2CO_3 (95 mg, 0.292 mmol). Work-up and purification were performed according to method A and the title compound was obtained as a white solid (39 mg, 68%). ^{1}H NMR (400 MHz, $CDCl_3$) δ 8.56 (d, 1H, *J* = 4.8 Hz), 8.37 (s, 1H), 7.81 (dd, 1H, *J* = 8.2, 1.4), 7.53-7.48 (m, 3H, *J* = 5.3, 1.8 Hz), 7.39-7-37 (m, 2H), 7.31 (dd, 1H, *J* = 8.2, 4.9 Hz), 4.78 (s, 2H). ^{13}C NMR (151 MHz, $CDCl_3$) δ 165.08, 155.20, 148.96, 146.84, 145.16, 143.50, 132.97, 130.13, 130.00, 125.24, 120.69, 118.86, 29.79. HRMS (ESI+) m/z calcd. for $C_{15}H_{11}N_5NaOS$ [MNa]+: 332.0577, found 332.0576.

6-Phenyl-2-((3-(p-tolyl)-1,2,4-oxadiazol-5-yl)methyl)pyridazin-3(2H)-one (**21**)
Prepared according to the general procedure by reacting 6-phenylpyridazin-3(2H)-one (**15**) (52 mg, 0.296 mmol) and 5-(chloromethyl)-3-(p-tolyl)-1,2,4-oxadiazole (**10**) (74 mg, 0.355 mmol) in the presence of Cs_2CO_3 (121 mg, 0.371 mmol). Work-up and purification were performed according to method A and the title compound was obtained as a white solid (90 mg, 88%). ^{1}H NMR (400 MHz, $CDCl_3$) δ 7.94 (d, 2H, *J* = 8.16 Hz), 7.77 (d, 1H, *J* = 9.64 Hz), 7.78-7.76 (m, 2H), 7.49-7.42 (m, 3H), 7.26 (d, 2H, *J* = 7.96 Hz), 7.12 (d, 1H, *J* = 9.76 Hz), 5.70 (s, 2H), 2.40 (s, 3H). ^{13}C NMR (101 MHz, $CDCl_3$) δ 173.88, 168.82, 159.53, 145.69, 141.79, 134.24, 131.46, 130.55, 130.01, 129.65, 129.16, 127.65, 126.22, 123.73, 47.91, 21.73. HRMS (ESI+) m/z calcd. for $C_{20}H_{16}N_4NaO_2$ [MNa]+: 367.1165, found 367.1165.

2-(Oxazolo[4,5-b]pyridin-2-ylmethyl)-6-phenylpyridazin-3(2H)-one (**22**)
Prepared according to the general procedure by reacting 6-phenylpyridazin-3(2H)-one (**15**) (51 mg, 0.296 mmol) and 2-(chloromethyl)oxazolo[4,5-b]pyridine (**11**) (52 mg, 0.308 mmol) in the presence of Cs_2CO_3 (154 mg, 0.473 mmol). Work-up and purification were performed according to method A and the title compound was obtained as a white solid 40 mg, 44%). ^{1}H NMR (400 MHz, $CDCl_3$) δ 8.60 (d, 1H, *J* = 4.6 Hz), 7.95 (dd, 1H, *J* = 8.2, 1.3 Hz), 7.79 (d, 1H, *J* = 9.8 Hz), 7.77-7.75 (m, 2H), 7.46-7-43 (m, 3H), 7.41 (dd, 1H, *J* = 8.2, 5.1 Hz), 7.12 (d, 1H, *J* = 9.8 Hz), 5.78 (s, 2H). ^{13}C NMR (101 MHz, $CDCl_3$) δ 164.55,

159.71, 154.91, 146.20, 145.72, 143.59, 134.25, 131.48, 130.55, 129.96, 129.12, 126.21, 120.59, 119.38, 49.72. HRMS (ESI+) m/z calcd. for $C_{17}H_{12}N_4NaO_2$ [MNa]+: 327.0852, found 327.0852.

5-((6-Bromo-1H-pyrazolo[4,3-b]pyridin-1-yl)methyl)-3-(p-tolyl)-1,2,4-oxadiazole (**23**)
Prepared according to the general procedure by reacting 6-bromo-1H-pyrazolo[4,3-b]pyridine (**16**) (50 mg, 0.252 mmol) and 5-(chloromethyl)-3-(p-tolyl)-1,2,4-oxadiazole (**10**) (60 mg, 0.288 mmol) in the presence of Cs_2CO_3 (107 mg, 0.328 mmol). Work-up and purification were performed according to method A and the title compound was obtained as a white solid (74 mg, 80%). ^{1}H NMR (400 MHz, $CDCl_3$) δ 8.68 (s, 1H), 8.31 (s, 1H), 8.12 (s, 1H), 7.90 (d, 2H, *J* = 8.24 Hz), 7.26 (d, 2H, *J* = 7.92 Hz), 5.84 (s, 2H), 2.40 (s, 3H). ^{13}C NMR (101 MHz, $CDCl_3$) δ 172.77, 168.84, 147.36, 142.06, 140.58, 136.01, 133.63, 129.63, 127.45, 123.07, 119.62, 118.72, 45.12, 21.60. HRMS (ESI+) m/z calcd. for $C_{16}H_{12}BrN_5NaO$ [MNa]+: 392.0117, found 392.0117.

2-((6-Bromo-1H-pyrazolo[4,3-b]pyridin-1-yl)methyl)oxazolo[4,5-b]pyridine (**24**)
Prepared according to the general procedure by reacting 6-bromo-1H-pyrazolo[4,3-b]pyridine (**16**) (49 mg, 0.247 mmol) and 2-(chloromethyl)oxazolo[4,5-b]pyridine (**11**) (47 mg, 0.278 mmol) in the presence of Cs_2CO_3 (164 mg, 0.503 mmol). Work-up and purification were performed according to method A and the title compound was obtained as a white solid (45 mg, 55%). ^{1}H NMR (600 MHz, $CDCl_3$) δ 8.65 (d, 1H, *J* = 1.8 Hz), 8.59 (dd, 1H, *J* = 4.8, 1.32 Hz), 8.31 (d, 1H, *J* = 0.8 Hz), 8.17 (t, 1H, *J* = 1.3), 7.82 (dd, 1H, *J* = 8.22, 1.38 Hz), 7.33 (dd, 1H, *J* = 8.22, 4.86 Hz), 5.90 (s, 2H). ^{13}C NMR (151 MHz, $CDCl_3$) δ 162.71, 154.85, 147.49, 147.27, 143.52, 140.67, 135.98, 133.82, 121.20, 119.96, 119.26, 118.89, 47.43. HRMS (ESI+) m/z calcd. for $C_{13}H_8BrN_5NaO$ [MNa]+: 351.9804, found 351.9804.

2-(2-((4-Phenyl-4H-1,2,4-triazol-3-yl)thio)ethyl)isoindoline-1,3-dione (**25**)
Prepared according to the general procedure by reacting 4-phenyl-4H-1,2,4-triazole-3-thiol (**13**) (57 mg, 0.322 mmol) and 2-(2-bromoethyl)isoindoline-1,3-dione (**12**) (108 mg, 0.425 mmol) in the presence of Cs_2CO_3 (195 mg, 0.598 mmol) at 70 °C. Work-up and purification were performed according to method B and the title compound was obtained as a white solid (66 mg, 58%). ^{1}H NMR (400 MHz, DMSO-d_6) δ 8.82 (s, 1H), 7.88-7.80 (m, 4H), 7.59- 7-48 (m, 3H), 7.45-7.40 (m, 2H), 3.97 (t, 2H, *J* = 6.2 Hz), 3.47 (t, 2H, *J* = 6.2 Hz). ^{13}C NMR (101 MHz, DMSO- d_6) δ 167.60, 148.51, 145.25, 134.41, 133.21, 131.50, 129.71, 129.37, 125.39, 123.06, 36.74, 30.36. HRMS (ESI+) m/z calcd. for $C_{18}H_{14}N_4NaO_2S$ [MNa]+: 373.0730, found 373.0729.

2-(2-(6-Oxo-3-phenylpyridazin-1(6H)-yl)ethyl)isoindoline-1,3-dione (**26**)
Prepared according to the general procedure by reacting 6-phenylpyridazin-3(2H)-one (**15**) (52 mg, 0.302 mmol) and 2-(2-bromoethyl)isoindoline-1,3-dione (**12**) (114 mg, 0.449 mmol) in the presence of Cs_2CO_3 (191 mg, 0.586 mmol) at 70 °C. Work-up and purification were performed according to method B and the title compound was obtained as a white solid (90 mg, 86%). ^{1}H NMR (400 MHz, $CDCl_3$) δ 7.78-7.73 (m, 2H), 7.66-7-61 (m, 3H), 7.46-7.42 (m, 2H), 7.34-7.22 (m, 3H), 7.00 (d, 1H, *J* = 9.7 Hz), 4.59-4.57 (m, 2H), 4.24-4.21 (m, 2H). ^{13}C NMR (101 MHz, $CDCl_3$) δ 168.30, 160.08, 144.66, 134.37, 134.00, 132.07, 130.38, 130.09, 129.28, 128.76, 125.60, 123.33, 49.91, 36.71. HRMS (ESI+) m/z calcd. for $C_{20}H_{15}N_3NaO_3$ [MNa]+: 368.1006, found 368.1006.

2-(2-[1,2,4]Triazolo[4,3-α]pyridine-3-ylthio)ethyl)isoindoline-1,3-dione (**27**)
Prepared according to the general procedure by reacting [1,2,4]triazolo[4,3-α]pyridine-3-thiol (**14**) (52.6 mg, 0.348 mmol) and 2-(2-bromoethyl)isoindoline-1,3-dione (**12**) (137 mg, 0.544 mmol)in the presence of Cs_2CO_3 (353 mg, 1.08 mmol) at 70 °C. Work-up and purification were performed according to method B and the title compound was obtained as a white solid (18 mg, 16%). ^{1}H NMR (400 MHz, $CDCl_3$) δ 8.09 (d, *J* = 7.62 Hz, 1H), 7.88 (d, *J* = 8.66 Hz, 1H), 7.81 (dd, *J* = 5.39 Hz, 3.00 Hz, 2H), 7.72 (dd, *J* = 5.52 Hz, 3.05 Hz, 2H), 7.40 (m, 1H), 7.00 (t, *J* = 6.81 Hz, 1H), 4.06 (t, *J* = 6.19 Hz, 2H), 3.59 (t, *J* = 6.75 Hz, 2H). ^{13}C NMR (400MHz, $CDCl_3$) δ 168.0, 149.7, 140.9. 134.8, 131.8, 130.4, 124.8, 123.4, 115.4, 115.1, 37.9, 32.2. HRMS (ESI+) m/z calcd. for $C_{16}H_{12}N_4O_2S$ [MNa]+: 347.0579, found 347.0573.

6-(2,4-Difluorophenyl)-2-((3-(pyrimidin-2-yl)-1,2,4-oxadiazol-5-yl)methyl)pyridazin-3(2H)-one (**34**)
Prepared according to the general procedure by reacting 6-(2,4-difluorophenyl)pyridazin-3(2H)-one (**32d**) (71 mg, 0.340 mmol) and 5-(chloromethyl)-3-(pyrimidin-2-yl)-1,2,4-oxadiazole (**33**) (98 mg, 0.499 mmol) in the presence of Cs_2CO_3 (358 mg, 1.09 mmol). Work-up and purification were performed according to method B and the title compound was obtained as an off-white solid (4 mg, 4%). ^{1}H NMR (600 MHz, $CDCl_3$) δ 8.99 (s, 2H), 7.70 (m, 2H), 7.47 (s, 1H), 7.07 (d, *J* = 9.82 Hz, 1H), 6.96 (td, *J* = 8.23 Hz, 2.55 Hz, 1H), 6.92 (ddd, *J* = 11.23 Hz, 8.48 Hz. 2.49 Hz, 1H), 5.77 (s, 2H). ^{13}C NMR (151 MHz, $CDCl_3$) δ 163.9 (dd, 252.9 Hz, 12.04 Hz), 160.5 (dd, 252.9 Hz, 12.20 Hz), 159.4, 159.1, 158.0, 142.0 (d, *J* = 1.8 Hz), 138.9, 135.1, 133.9 (d, *J* = 8.64 Hz), 132.5, 131.2 (dd, *J* = 9.98 Hz, 4.06 Hz), 129.9, 118.8 (dd, *J* = 11.79 Hz, 3.84 Hz), 112.4 (dd, *J* = 21.56 Hz, 3.65 Hz), 104.7 (t, *J* = 26.54 Hz), 47.74. HRMS (ESI+) m/z calcd. for $C_{17}H_{10}F_2N_6O_2$ [MNa]+: 391.07326, found 391.0725.

6-(3-Fluorophenyl)-2-((3-(pyrimidin-2-yl)-1,2,4-oxadiazol-5-yl)methyl)pyridazin-3(2H)-one (**35**)
Prepared according to the general procedure by reacting 6-(3-fluorophenyl)pyridazin-3(2H)-one (**32a**) (37 mg, 0.194 mmol) and 5-(chloromethyl)-3-(pyrimidin-2-yl)-1,2,4-oxadiazole (**33**) (60 mg, 0.306 mmol) in the presence of Cs_2CO_3 (131 mg, 0.402 mmol). Work-up and purification were performed according to method B and the title compound was obtained as a white solid (11 mg, 17%). ^{1}H NMR (600 MHz, DMSO-d_6) δ 9.00 (d, *J* = 4.90 Hz, 2H), 8.23 (d, *J* = 9.80, 1H), 7.76 (m, 2H), 7.70 (t, *J* = 4.90 Hz, 1H), 7.56 (td, *J* = 7.98 Hz, 6.15 Hz, 1H), 7.33 (td, *J* = 8.55 Hz, 2.40 Hz, 1H), 7.21 (d, *J* = 9.80 Hz, 1H), 5.82 (s, 2H). ^{13}C NMR (151 MHz, DMSO-d_6) δ 176.6, 168.0, 163.0 (d, *J* = 243.5 Hz), 159.2, 158.8, 155.5, 143.8 (d, *J* = 2.81 Hz), 136.7 (d, *J* = 8.08 Hz), 132.5, 131.6 (d, *J* = 8.27 Hz), 130.5, 123.5, 122.58 (d, *J* = 2.54 Hz), 117.0 (d, *J* = 21.11 Hz), 113.2 (d, *J* = 23.59 Hz), 48.69. HRMS (ESI+) m/z calcd. For $C_{17}H_{11}FN_6O_2$ [MNa]+: 373.0820, found 373.0819.

6-(3-Fluorophenyl)-2-((3-(p-tolyl)-1,2,4-oxadiazol-5-yl)methyl)pyridazin-3(2H)-one (**36**)
Prepared according to the general procedure by reacting 6-(3-fluorophenyl)pyridazin-3(2H)-one (**32a**) (28 mg, 0.147 mmol) and 5-(chloromethyl)-3-(p-tolyl)-1,2,4-oxadiazole (**10**) (42 mg, 0.221 mmol) in the presence of Cs_2CO_3 (106 mg, 0.326 mmol). Work-up and purification were performed according to method B and the title compound was obtained as a brown solid (11 mg, 21%). ^{1}H NMR (400 MHz, DMSO-d_6) δ 8.22 (d, *J* = 9.88 Hz, 1H), 7.86 (d, *J* = 8.05 Hz, 2H), 7.76 (t, *J* = 9.26 Hz, 2H), 7.56 (td, *J* = 8.11 Hz, 6.15 Hz, 1H), 7.35 (d, *J* = 8.02 Hz, 2H), 7.31 (dd, *J* = 8.34 Hz, 2.36 Hz, 1H), 7.21 (d, *J* = 9.76 Hz, 1H), 5.77 (s, 2H), 2.36 (s, 3H). ^{13}C NMR (101 MHz, DMSO-d_6) δ 175.5, 168.2, 163.1 (d, *J* = 243.9 Hz), 159.2, 143.7 (d, *J* = 2.81 Hz), 142.2, 136.7 (d, *J* = 8.24 Hz), 132.4, 131.6 (d, *J* = 8.17 Hz), 130.5, 130.3, 127.5,

123.4, 122.5 (d, *J* = 2.79 Hz), 117.0 (d, 21.1 Hz), 113.2 (d, 23.5 Hz), 48.6, 21.5. HRMS (ESI+) m/z calcd. for $C_{20}H_{15}FN_4O_2$ [MNa]+: 385.1071, found 385.1070.

6-(2,4-Difluorophenyl)-2-(oxazolo[4,5-b]pyridin-2-ylmethyl)pyridazin-3(2H)-one (**37**)
Prepared according to the general procedure by reacting 6-(2,4-difluorophenyl)pyridazin-3(2H)-one (**32d**) (25 mg, 0.120 mmol) and 2-(chloromethyl)oxazolo[4,5-b]pyridine (**11**) (27 mg, 0.160 mmol) in the presence of Cs_2CO_3 (66 mg, 0.203 mmol). Work-up and purification were performed according to method B and the title compound was obtained as a white solid (25 mg, 61%). ^1H NMR (600 MHz, $CDCl_3$) δ 8.57 (d, 1H, *J* = 4.4 Hz), 7.82 (dd, 1H, *J* = 8.2, 1.1 Hz), 7.74-7.70 (m, 2H), 7.30 (d, 1H, *J* = 8.1, 4.9 Hz), 7.08 (d, 1H, *J* = 9.8 Hz), 6.97-6.89 (m, 2H), 5.74 (s, 2H). ^{13}C NMR (151 MHz, $CDCl_3$) δ 164.01, 163.91 (dd, *J* = 253.0, 12.2 Hz), 160.72 (dd, *J* = 252.5 12.2 Hz), 159.49, 155.28, 146.82, 143.40, 142.01 (d, *J* = 2,5 Hz), 133.93 (d, *J* = 8.9 Hz), 131.39 (dd, *J* = 10.1, 4.4 Hz), 130.00, 120.57, 119.08 (dd, *J* = 11.9, 4.1 Hz), 118.88, 112.53 (dd, *J* = 21.6, 4.1 Hz), 104.83 (t, *J* = 26.1 Hz), 49.58. HRMS (ESI+) m/z calcd. for $C_{17}H_{10}F_2N_4NaO_2$ [MNa]+: 363.0664, found 363.0664.

6-Phenyl-2-((3-(pyrimidin-2-yl)-1,2,4-oxadiazol-5-yl)methyl)pyridazin-3(2H)-one (**38**)
Prepared according to the general procedure by reacting 6-phenylpyridazin-3(2H)-one (**15**) (52 mg, 0.300 mmol) and 5-(chloromethyl)-3-(pyrimidin-2-yl)-1,2,4-oxadiazole (**33**) (43 mg, 0.220 mmol) in the presence of Cs_2CO_3 (174 mg, 0.534 mmol). Work-up and purification were performed according to method B and the title compound was obtained as a white solid (33 mg, 46%). ^1H NMR (400 MHz, DMSO-d_6) δ 9.00 (d, *J* = 4.84 Hz, 2H), 8.19 (d, *J* = 9.83 Hz, 1H), 7.91 (dd, *J* = 8.19 Hz, 1.56 Hz, 2H), 7.70 (t, *J* = 4.93 Hz, 1H), 7.50 (m, 3H), 7.20 (d, *J* = 9.79 Hz, 1H), 5.81 (s, 2H). ^{13}C NMR (101 MHz, DMSO-d_6) δ 176.6, 167.9, 159.2, 158.7, 155.5, 145.1, 134.3, 132.6, 130.5, 130.2, 129.5, 126.4, 123.5, 48.61. HRMS (ESI+) m/z calcd. for $C_{17}H_{12}N_6O_2$ [MNa]+: 355.0914, found 355.0914.

2-(Oxazolo[4,5-b]pyridin-2-ylmethyl)-6-(pyridin-4-yl)pyridazin-3(2H)-one (**39**)
Prepared according to the general procedure by reacting 6-(pyridin-4-yl)pyridazin-3(2H)-one (**32b**) (45 mg, 0.260 mmol) and 2-(chloromethyl)oxazolo[4,5-b]pyridine (**11**) in the presence of Cs_2CO_3 (127 mg, 0.390 mmol). Work-up and purification were performed according to method B and the title compound was obtained as a pale yellow solid (36 mg, 45%). ^1H NMR (400 MHz, $CDCl_3$) δ 8.73 (d, 2H, *J* = 4.1 Hz), 8.57 (dd, 1H, *J* = 4.8, 1.1 Hz), 7.82 (dd, 1H, *J* = 8.2, 1.2 Hz), 7.81 (d, 1H, *J* = 9.8 Hz), 7.74 (d, 2H, *J* = 5.9 Hz), 7.31 (dd, 1H, *J* = 8.2, 4.9 Hz), 7.17 (d, 1H, *J* = 9.8 Hz), 5.76 (s, 2H). ^{13}C NMR (151 MHz, $CDCl_3$) δ 163.63, 159.51, 155.26, 150.06, 147.01, 143.37, 142.68, 142.12, 130.98, 130.46, 120.66, 120.33, 118.80, 49.88. HRMS (ESI+) m/z calcd. for $C_{16}H_{11}N_5NaO_2$ [MNa]+: 328.0805, found 328.0805.

6-(5-Acetylthiophen-2-yl)-2-((3-(pyrimidin-2-yl)-1,2,4-oxadiazol-5-yl)methyl)pyridazin-3(2H)-one (**40**)
Prepared according to the general procedure by reacting 6-(5-acetylthiophen-2-yl)pyridazin-3(2H)-one (**32c**) (36 mg, 0.165 mmol) and 5-(chloromethyl)-3-(pyrimidin-2-yl)-1,2,4-oxadiazole (**33**) (54 mg, 0.275 mmol) in the presence of Cs_2CO_3 (188 mg, 0.578 mmol). Work-up and purification were performed according to method B and the title compound was obtained as a white solid (13 mg, 21%). ^1H NMR (400 MHz, DMSO-d_6) δ 9.00 (d, *J* = 4.90 Hz, 2H), 8.25 (d, *J* = 9.86 Hz, 1H), 7.98 (d, *J* = 4.11 Hz, 1H), 7.88 (d, *J* = 4.04 Hz, 1H), 7.71 (t, *J* = 4.85 Hz, 1H), 7.22 (d, *J* = 9.96 Hz, 1H), 5.79 (s, 2H), 2.55 (s, 3H). ^{13}C NMR (101 MHz, DMSO-d_6) δ 191.6, 176.4, 167.9, 158.9, 158.7, 158.1, 155.4, 145.5, 141.0, 135.0, 131.7,

130.5, 128.9, 123.5, 48.62, 27.01. HRMS (ESI+) m/z calcd. for $C_{17}H_{12}N_6O_3S$ [MNa]+: 403.0584, found 403.0583.

6-(5-Acetylthiophen-2-yl)-2-(oxazolo[4,5-b]pyridin-2-ylmethyl)pyridazin-3(2H)-one (**41**)
Prepared according to the general procedure by reacting 6-(5-acetylthiophen-2-yl)pyridazin-3(2H)-one (**32c**) (33 mg, 0.149 mmol) and 2-(chloromethyl)oxazolo[4,5-b]pyridine (**11**) (42 mg, 0.250 mmol) in the presence of Cs_2CO_3 (123 mg, 0.377 mmol). Work-up and purification were performed according to method B and the title compound was obtained as a brown solid (3 mg, 6%). ^{1}H NMR (600MHz, DMSO-d_6) δ 8.53 (dd, *J* = 4.86 Hz, 1.36 Hz, 1H), 8.25 (d, *J* =9.84 Hz, 1H), 8.22 (dd, *J* = 8.21 Hz, 1.37 Hz, 1H), 7.97 (d, *J* = 4.04 Hz, 1H), 7.87 (d, *J* = 4.06 Hz, 1H), 7.46 (dd, *J* = 8.49 Hz, 4.89 Hz, 1H), 7.22 (d, *J* = 9.81 Hz, 1H), 5.70 (s, 2H), 2.54 (s, 3H). ^{13}C NMR (151 MHz, DMSO-d_6) δ 191.5, 164.9, 159.0, 155.0, 146.9, 145.6, 145.4, 143.1, 140.8, 135.0, 131.5, 130.5, 128.7, 121.3, 119.8, 50.27, 27.02. HRMS (ESI+) m/z calcd. for $C_{17}H_{12}N_4O_3S$ [MNa]+: 375.0522, found 375.0522.

2-(((6-Chloro-[1,2,4]triazolo[4,3-b]pyridazin-3-yl)thio)methyl)oxazolo[4,5-b]pyridine (**42**)
Prepared according to the general procedure by reacting 6-chloro-[1,2,4]triazolo[4,3-b]pyridazine-3-thiol (**31a**) (20 mg, 0.107 mmol) and 2-(chloromethyl)oxazolo[4,5-b]pyridine (**11**) (27 mg, 0.160 mmol) in the presence of Cs_2CO_3 (65 mg, 0.199 mmol). Work-up and purification were performed according to method B and the title compound was obtained as a light yellow solid (13 mg, 38%). ^{1}H NMR (600 MHz, DMSO-d_6) δ 8.49 (d, 1H, *J* = 9.5 Hz), 8.48 (dd, 1H, *J* = 4.9, 1.5 Hz), 8.16 (dd, 1H, *J* = 8.2, 1.5 Hz), 7.50 (d, 1H, *J* = 9.5 Hz), 7.44 (dd, 1H, *J* = 8.1, 4.9 Hz), 4.81 Hz). ^{13}C NMR (151 MHz, DMSO-d_6) δ 165.52, 154.71, 149.64, 146.34, 144.68, 142.87, 142.78, 127.27, 123.41, 120.89, 119.11, 29.35. HRMS (ESI+) m/z calcd. for $C_{12}H_7ClN_6NaOS$ [M+H]+: 340.9983, found 340.9983.

5-(((6-Chloro-[1,2,4]triazolo[4,3-b]pyridazin-3-yl)thio)methyl)-3-(p-tolyl)-1,2,4-oxadiazole (**43**)
Prepared according to the general procedure by reacting 6-chloro-[1,2,4]triazolo[4,3-b]pyridazine-3-thiol (**31a**) (20 mg, 0.107 mmol) and 5-(chloromethyl)-3-(p-tolyl)-1,2,4-oxadiazole (**10**) (27 mg, 0.129 mmol) in the presence of Cs_2CO_3 (70 mg, 0.214 mmol). Work-up and purification were performed according to method B and the title compound was obtained as a light yellow solid (6 mg, 16%). ^{1}H NMR (600 MHz, $CDCl_3$) δ 8.09 (d, 1H, *J* = 9.5), 7.86 (d, 2H, *J* = 8.1 Hz), 7.25 (d, 2H, *J* = 8.1 Hz), 7.12 (d, 1H, *J* = 9.5), ^{13}C NMR (151 MHz, $CDCl_3$) δ 174.79, 168.82, 150.21, 141.87, 129.67, 127.51, 126.58, 123.58, 122.91, 26.71, 21.73. HRMS (ESI+) m/z calcd. for $C_{15}H_{11}ClN_6NaOS$ [MNa]+: 381.0296, found 381.0296.

5-(((6-(Pyridin-4-yl)-[1,2,4]triazolo[4,3-b]pyridazin-3-yl)thio)methyl)-3-(pyrimidin-2-yl)-1,2,4-oxadiazole (**44**)
Prepared according to the general procedure by reacting 6-(pyridin-4-yl)-[1,2,4]triazolo[4,3-b]pyridazine-3-thiol (**31b**) (40 mg, 0.174 mmol) and 5-(chloromethyl)-3-(pyrimidin-2-yl)-1,2,4-oxadiazole (**33**) (42 mg, 0.214 mmol) in the presence of Cs_2CO_3 (108 mg, 0.552 mmol). Work-up and purification were performed according to method B and the title compound was obtained as an orange solid (44 mg, 66%). ^{1}H NMR (400 MHz, $CDCl_3$) δ 8.89 (d, 2H, *J* = 4.8 Hz), 8.75 (s, 2H), 8.27 (d, 1H, *J* = 9.7 Hz), 7.85 (d, 2H, *J* = 5.0 Hz), 7.61 (d, 1H, *J* = 9.7 Hz), 7.43 (t, 1H, *J* = 4.9 Hz), 4.81 (s, 2H). ^{13}C NMR (101

MHz, $CDCl_3$) δ 176.96, 168.05, 158.35, 158.11, 155.84, 151.65, 149.96, 145.45, 142.11, 126.36, 122.42, 121.61, 118.91, 27.21. HRMS (ESI+) m/z calcd. for $C_{17}H_{11}ClN_9NaOS$ [MNa]+: 412.0699, found 412.0699.

2-(((6-(Pyridin-4-yl)-[1,2,4]triazolo[4,3-b]pyridazin-3-yl)thio)methyl)oxazolo[4,5-b]pyridine (**45**)
Prepared according to the general procedure by reacting 6-(pyridin-4-yl)-[1,2,4]triazolo[4,3-b]pyridazine-3-thiol (**31b**) and 2-(chloromethyl)oxazolo[4,5-b]pyridine (**11**) (39 mg, 0.231 mmol) in the presence of Cs_2CO_3 (86 mg, 0.262 mmol). Work-up and purification were performed according to method B and the title compound was obtained as an orange solid (50 mg, 79%). ^{1}H NMR (400 MHz, $CDCl_3$) δ 8.78 (d, 2H, *J* = 6.1 Hz), 8.43 (dd, 1H, *J* = 4.9, 1.4 Hz), 8.25 (d, 1H, *J* = 9.7 Hz), 7.74 (d, 2H, *J* = 6.1 Hz), 7.71 (dd, 1H, *J* = 8.2, 1.4 Hz), 7.57 (d, 1H, *J* = 9.7 Hz), 7.18 (dd, 1H, *J* = 8.2, 4.9 Hz), 4.79 (s, 2H). ^{13}C NMR (101 MHz, DMSO) δ 165.61, 154.58, 151.61, 150.53, 146.16, 145.22, 143.01, 142.68, 140.54, 125.98, 121.04, 120.66, 120.12, 118.90, 29.55. HRMS (ESI+) m/z calcd. for $C_{17}H_{11}N_7NaOS$ [MNa]+: 384.0638, found 384.0638.

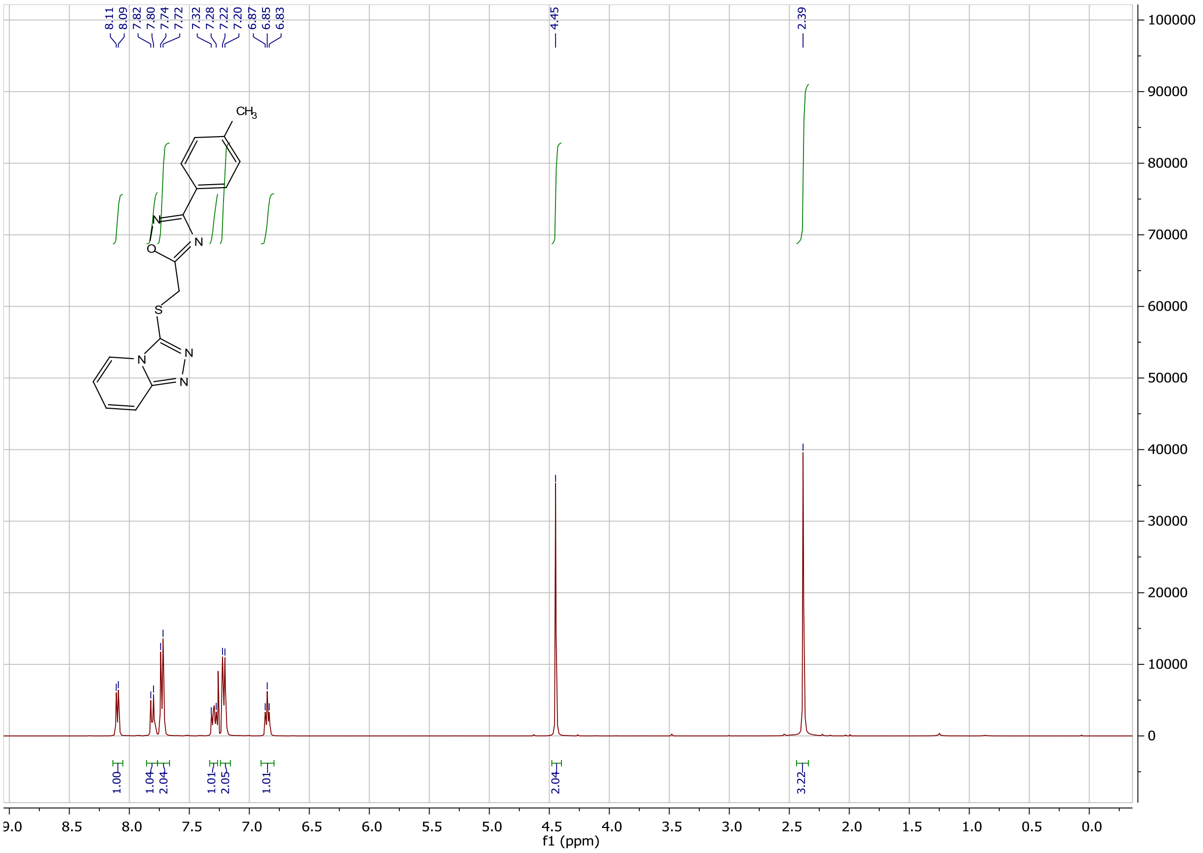

Figure S1: ^{1}H NMR for compound **17**.

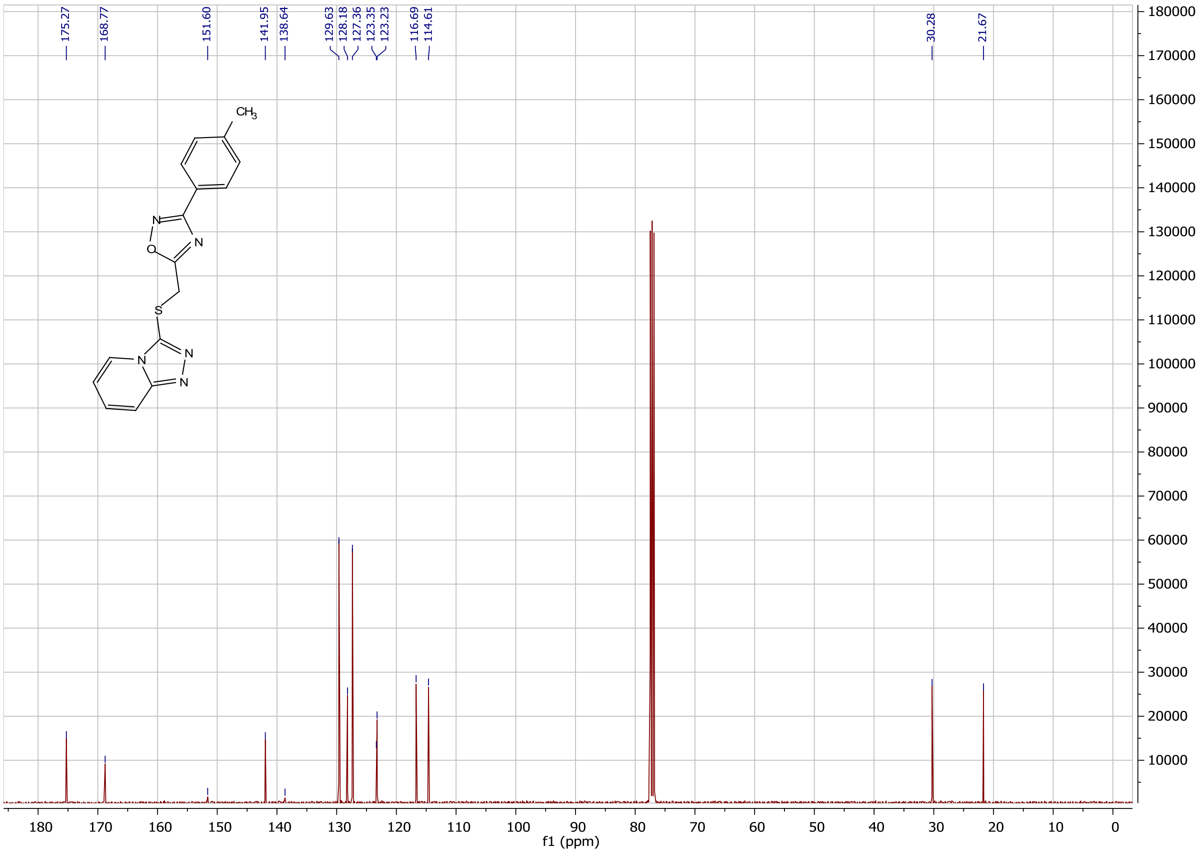

Figure S2: ^{13}C NMR for compound **17.**

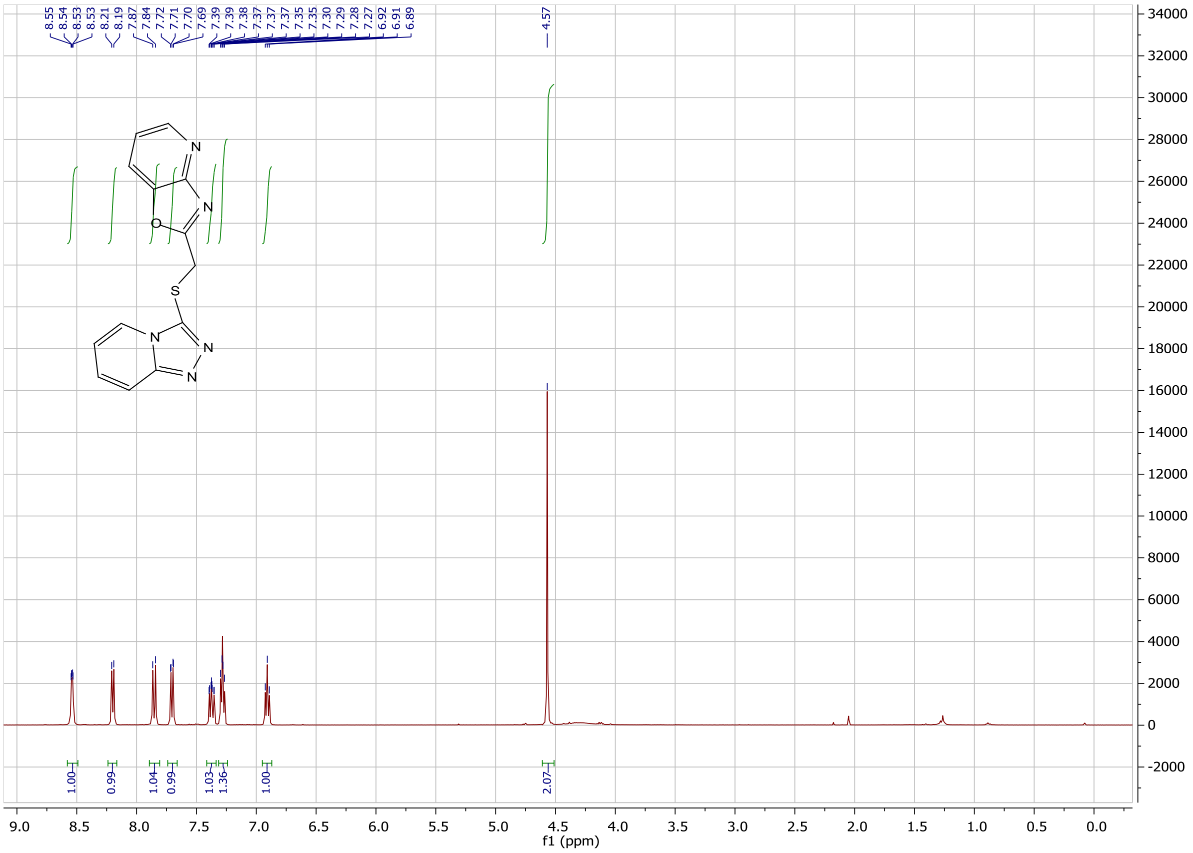

Figure S3: ^{1}H NMR for compound **18**.

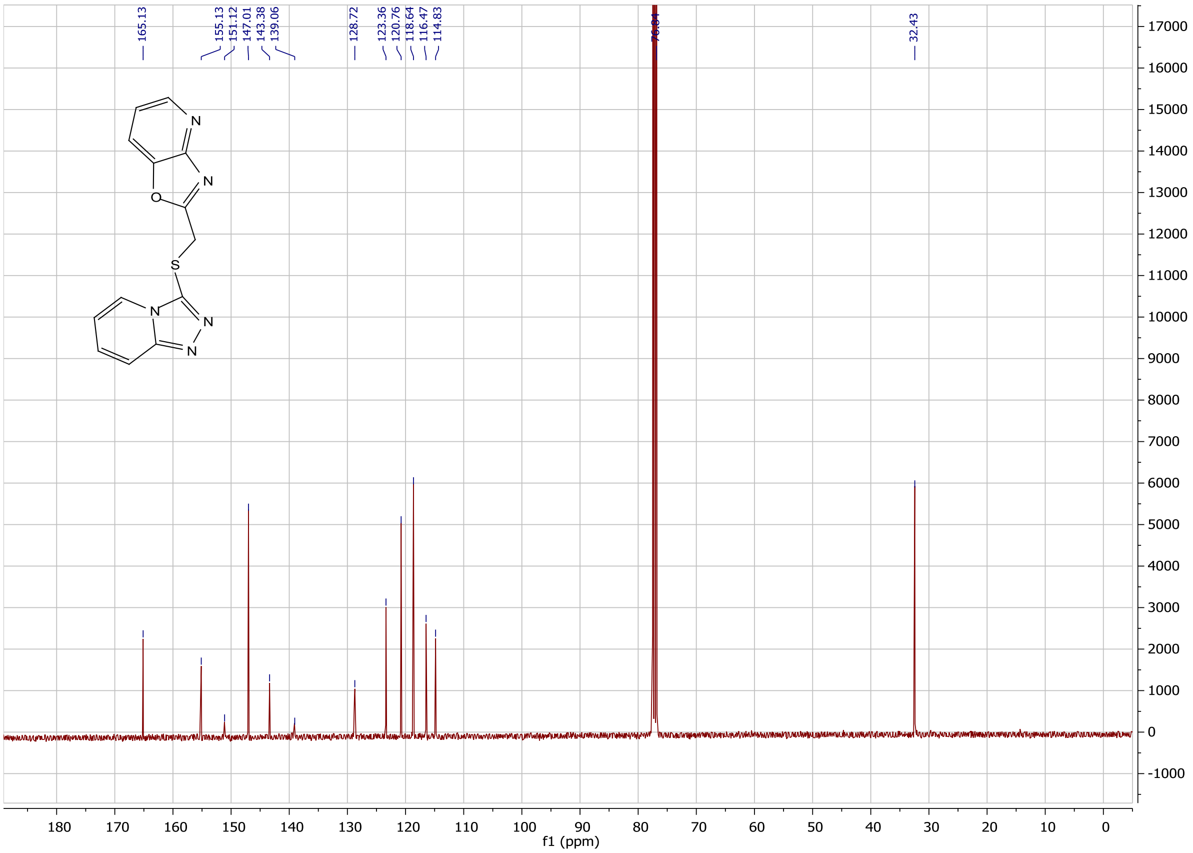

Figure S4: ^{13}C NMR for compound **18**.

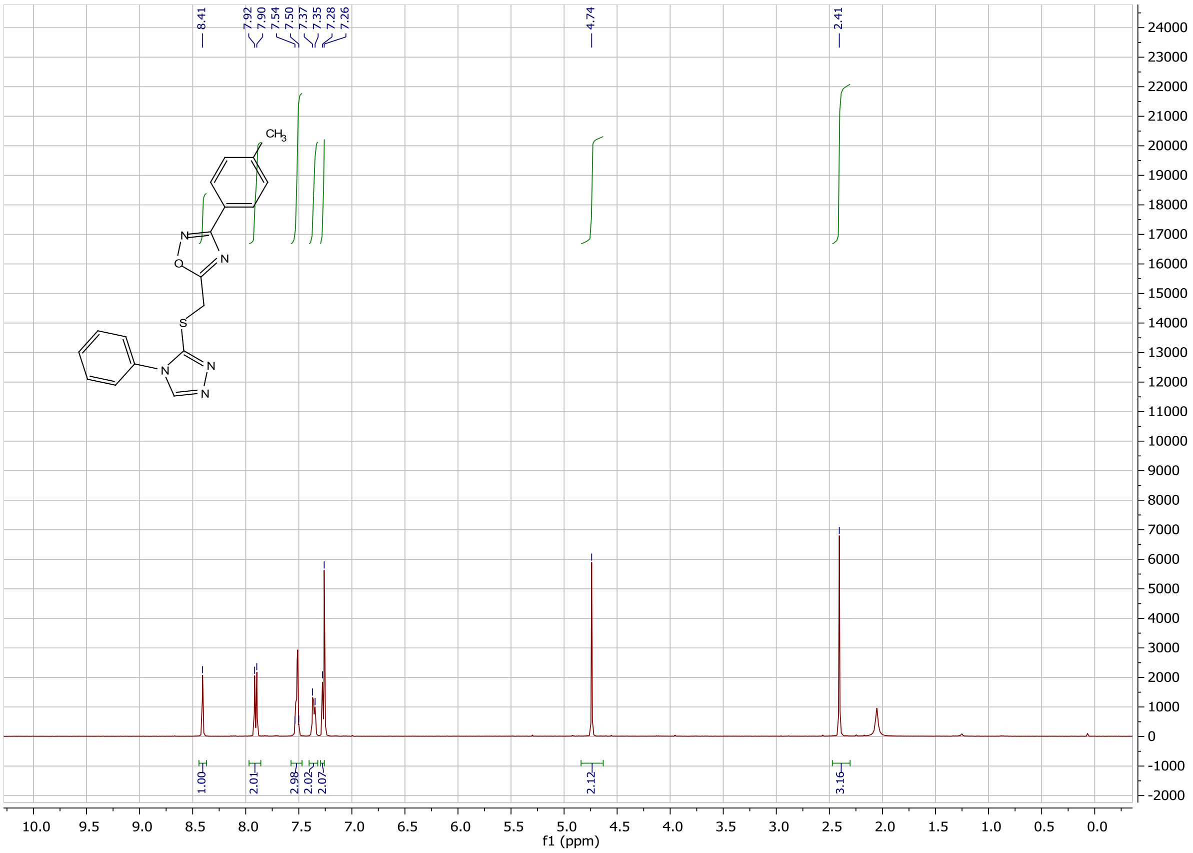

Figure S5: ^{1}H NMR for compound **19**.

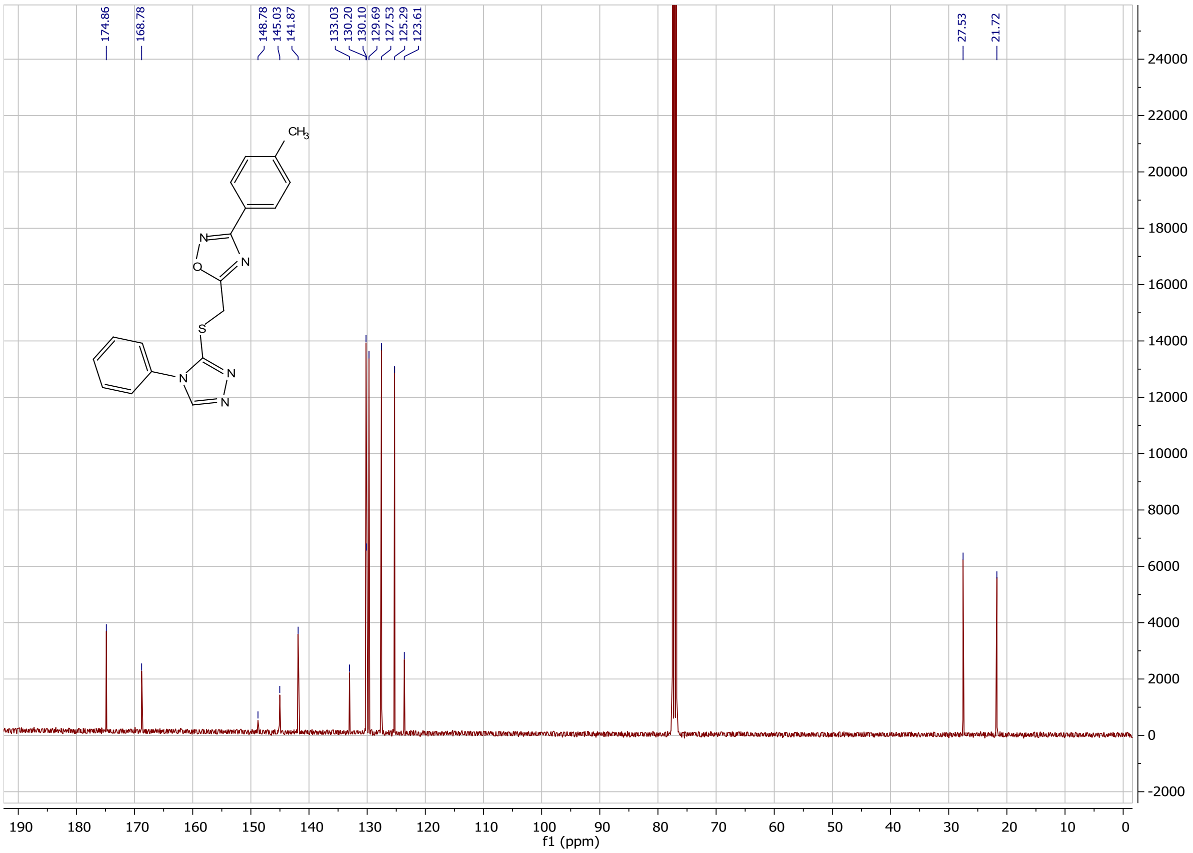

Figure S6: ^{13}C NMR for compound **19**.

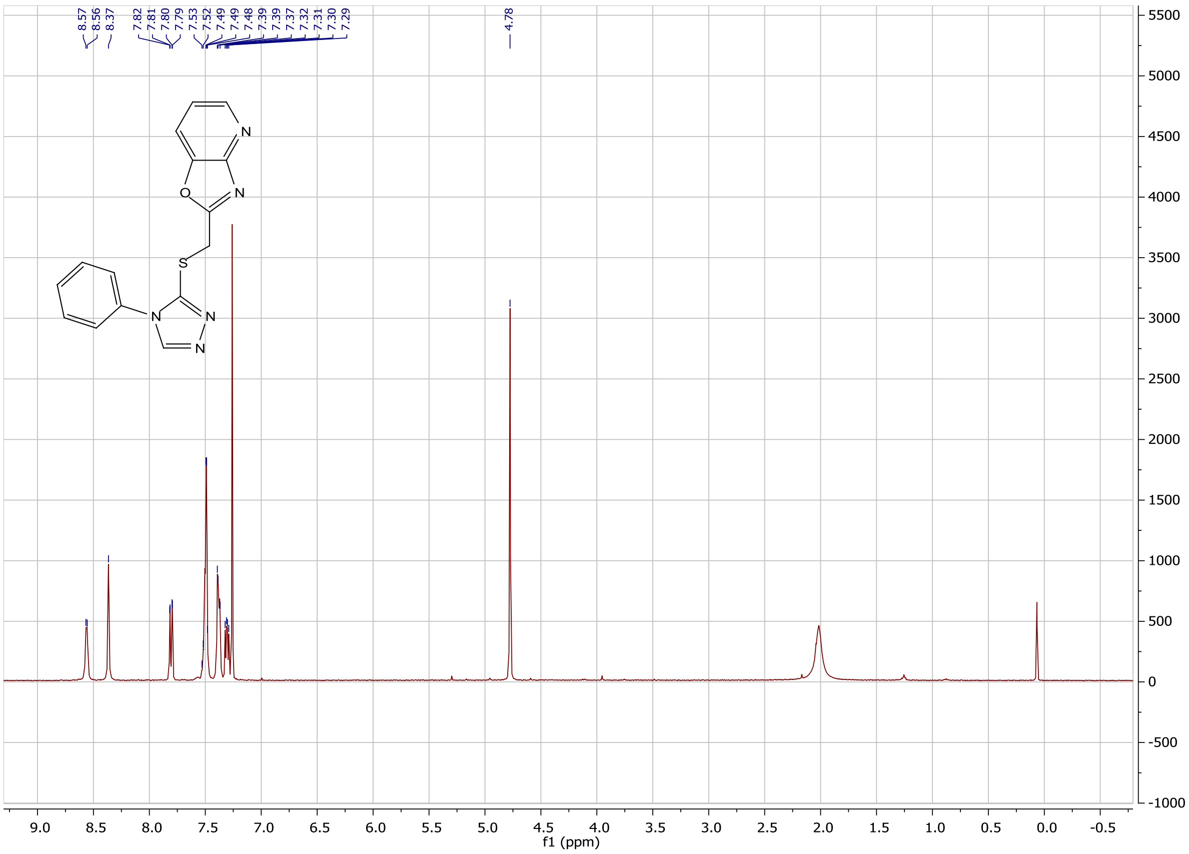

Figure S7: ^{1}H NMR for compound **20**.

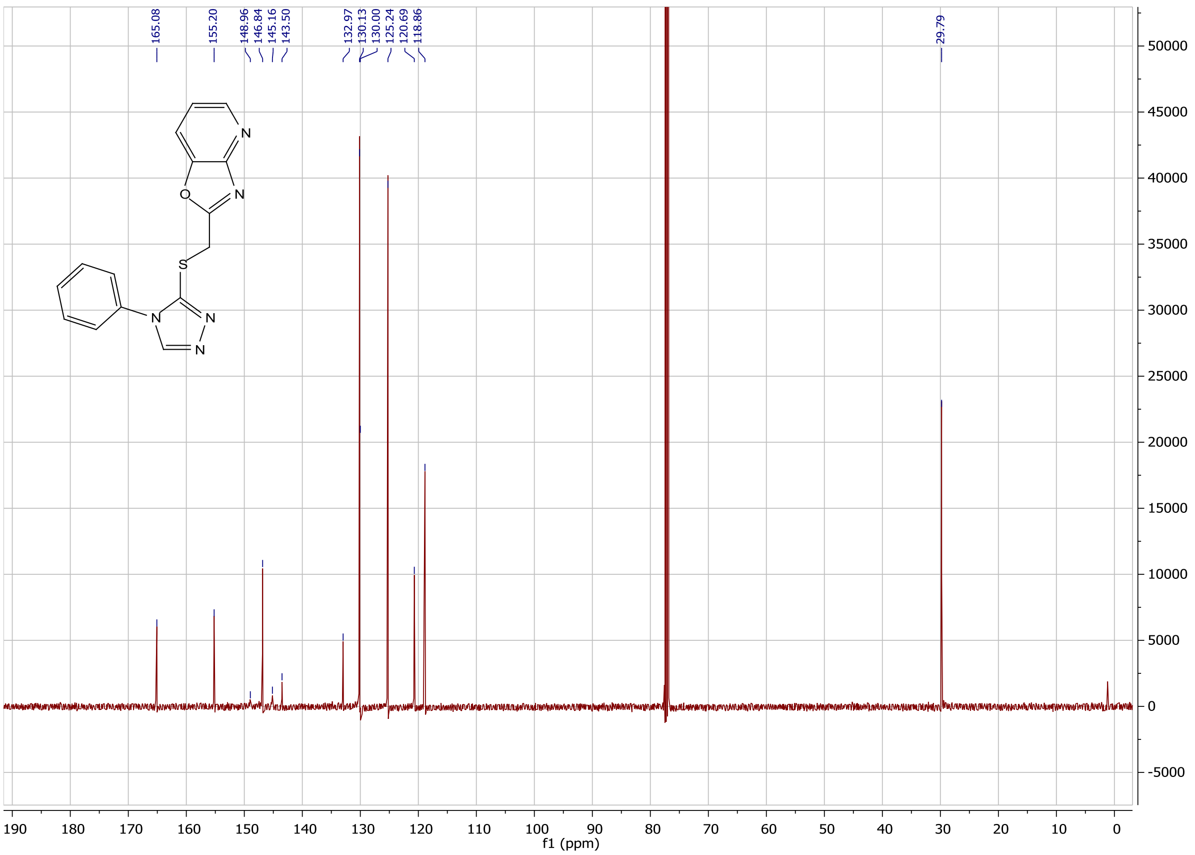

Figure S8: ^{13}C NMR for compound **20**.

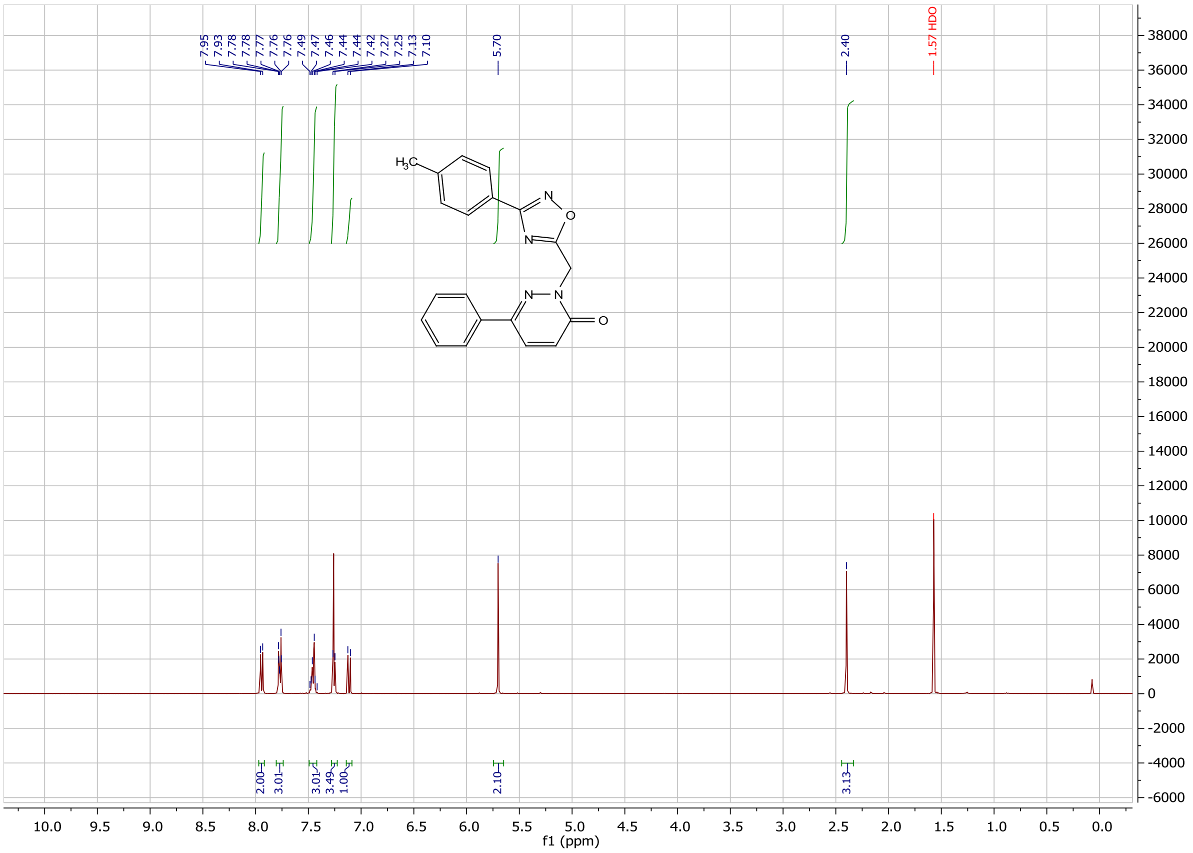

Figure S9: ^{1}H NMR for compound **21**.

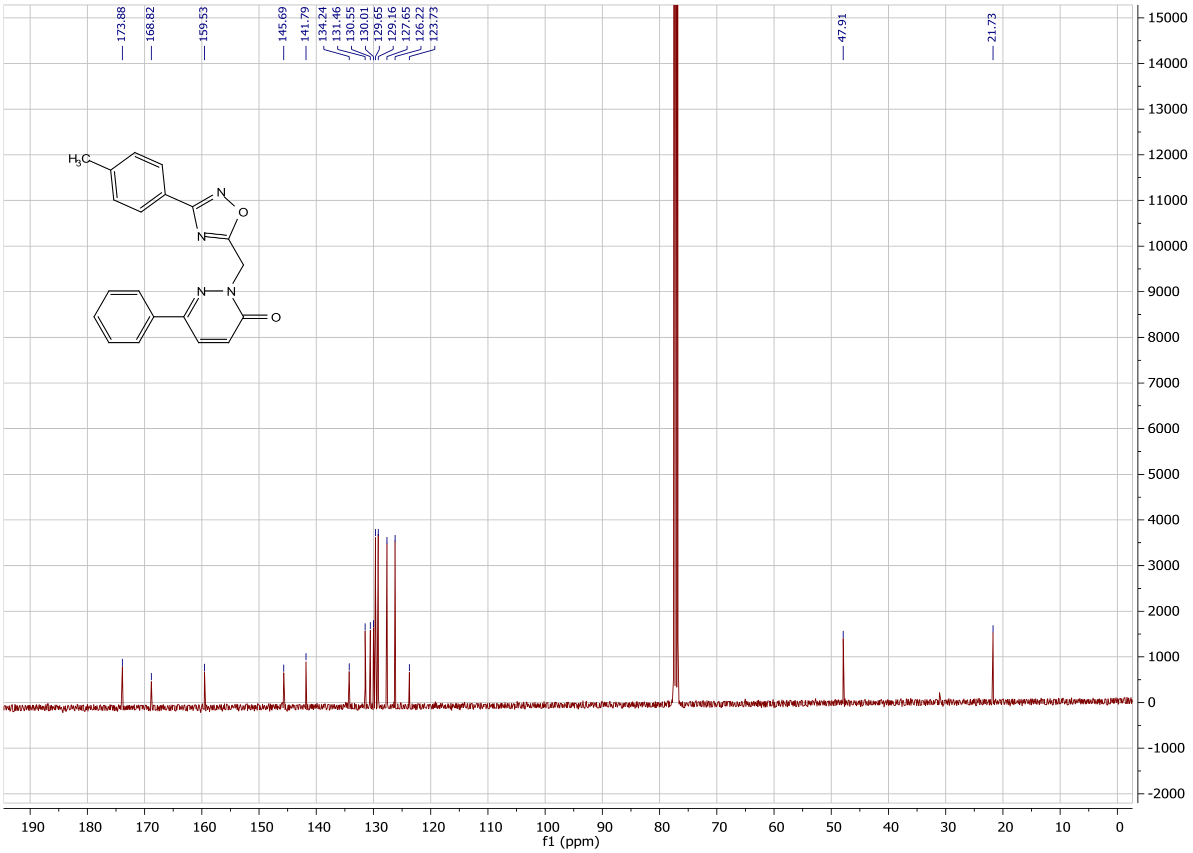

Figure S10: ^{13}C NMR for compound **21**.

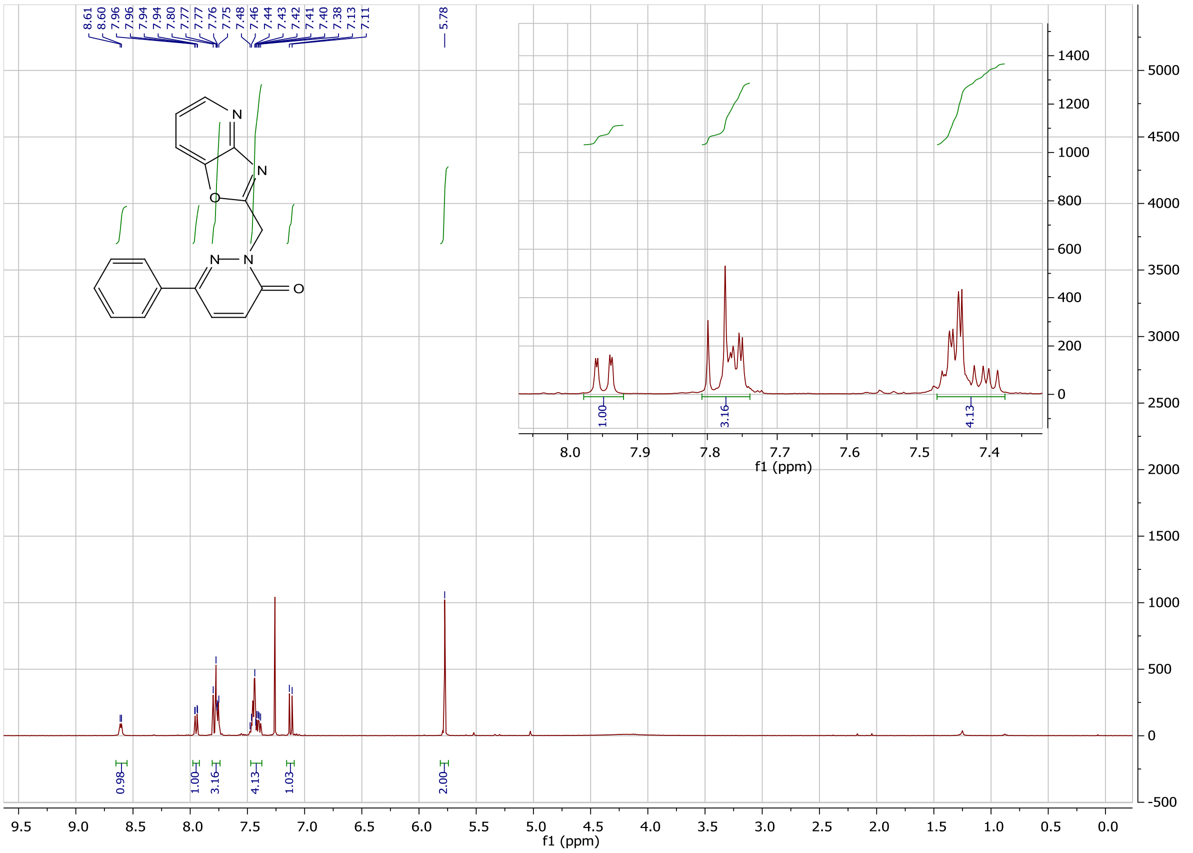

Figure S11: ^{1}H NMR for compound **22**.

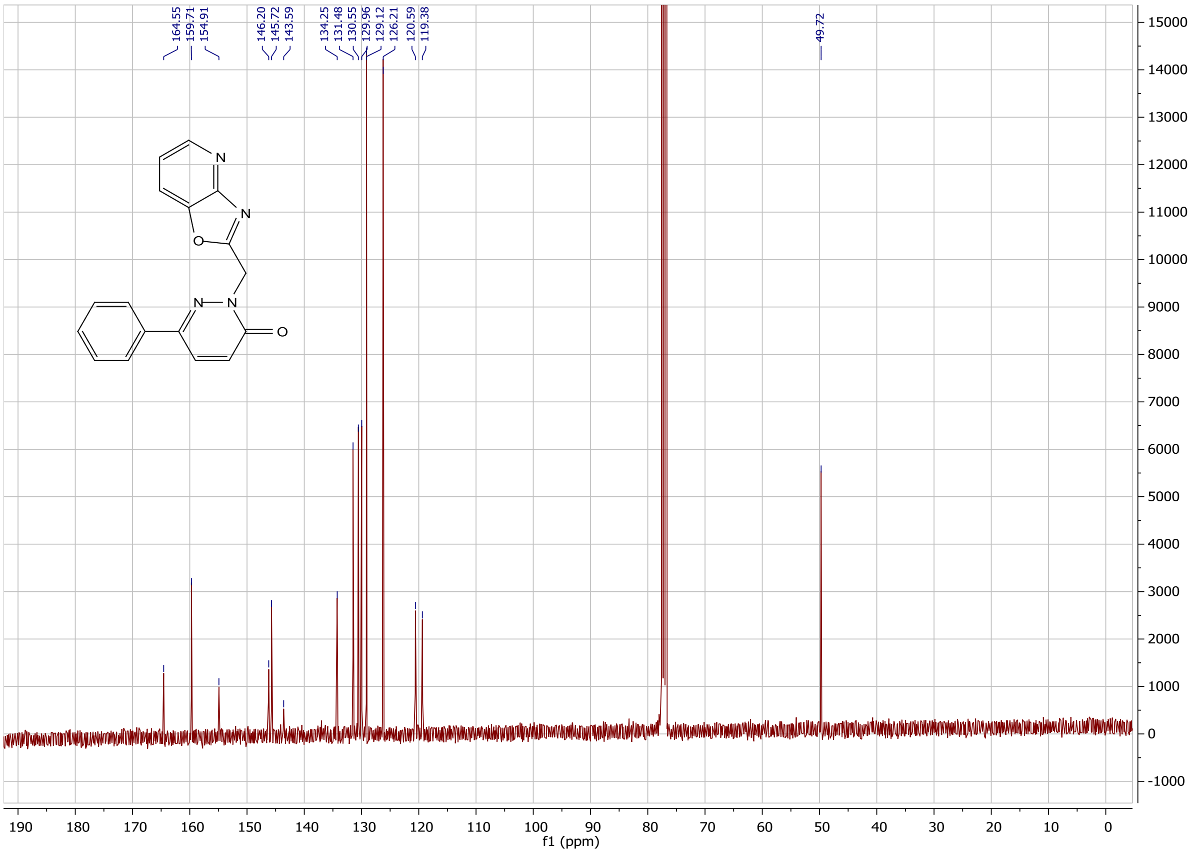

Figure S12: ^{13}C NMR for compound **22**.

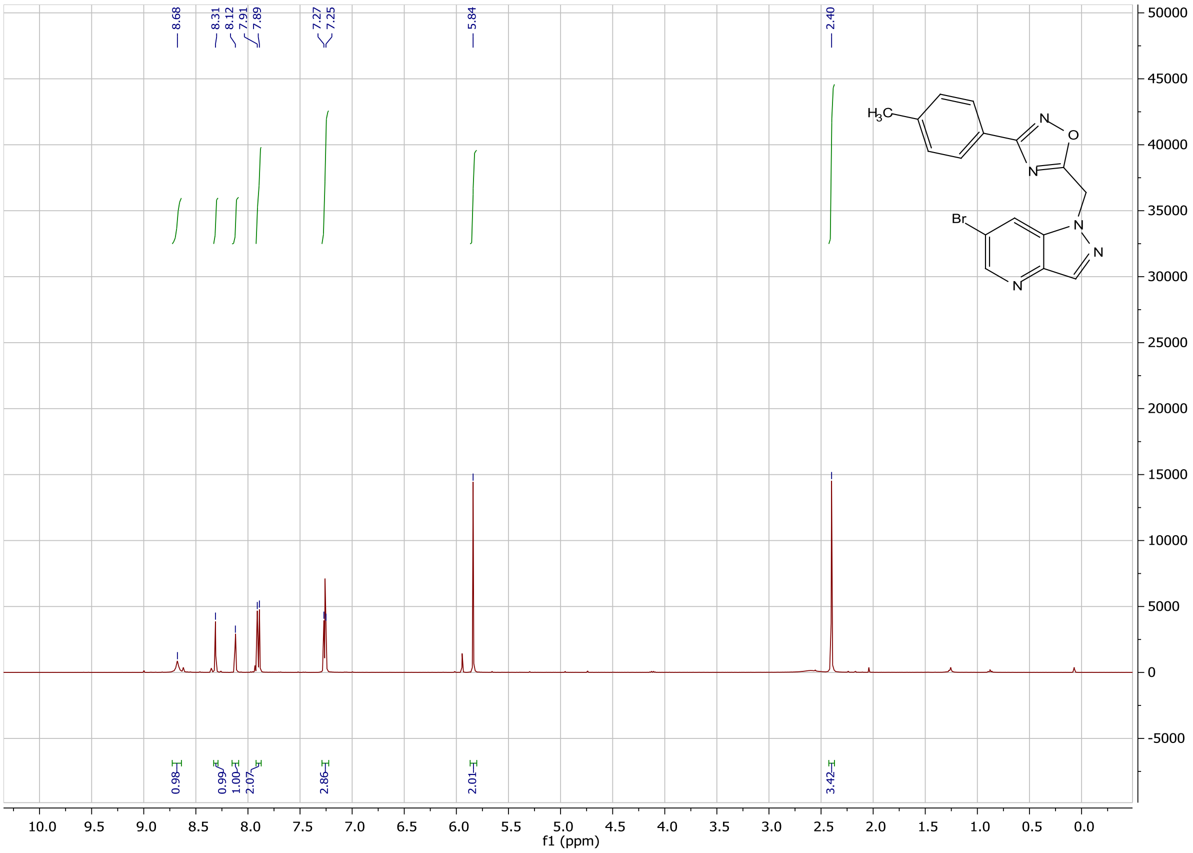

Figure S13: ^{1}H NMR for compound **23**.

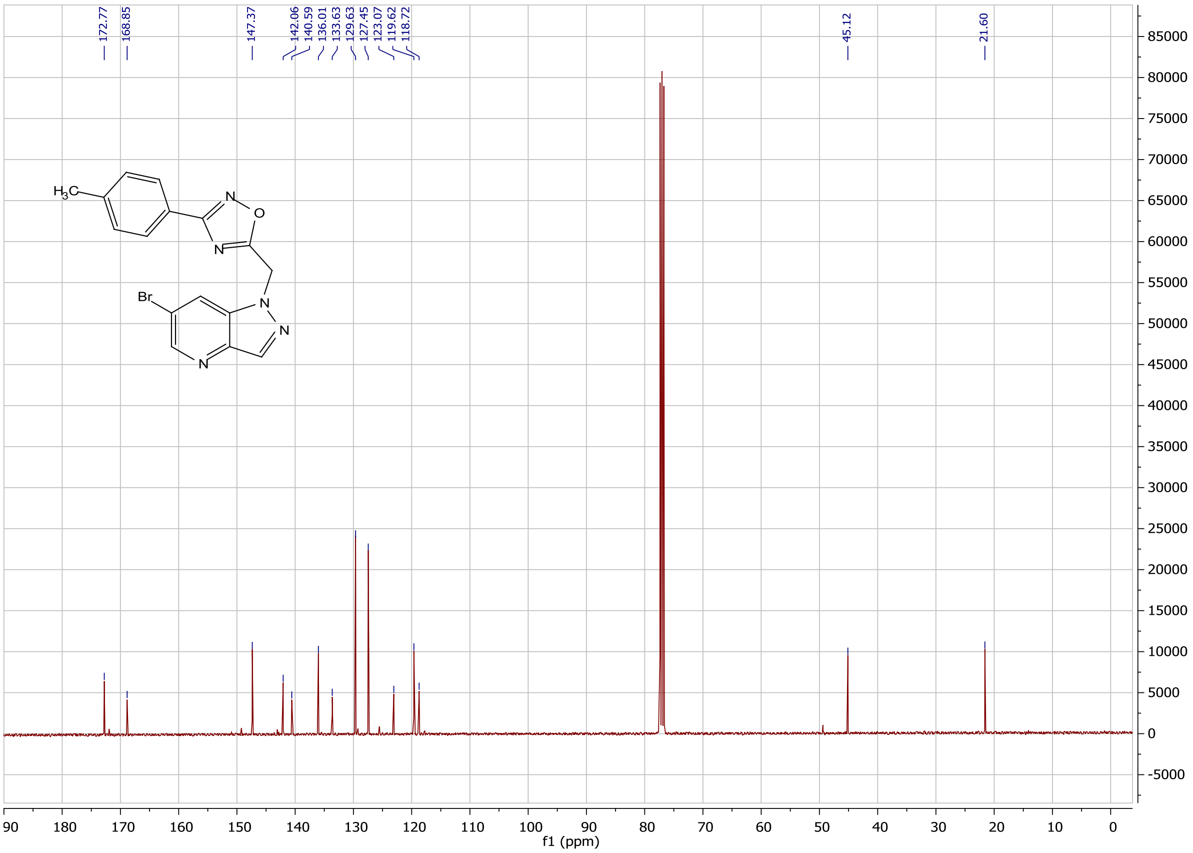

Figure S14: ^{13}C NMR for compound **23**.

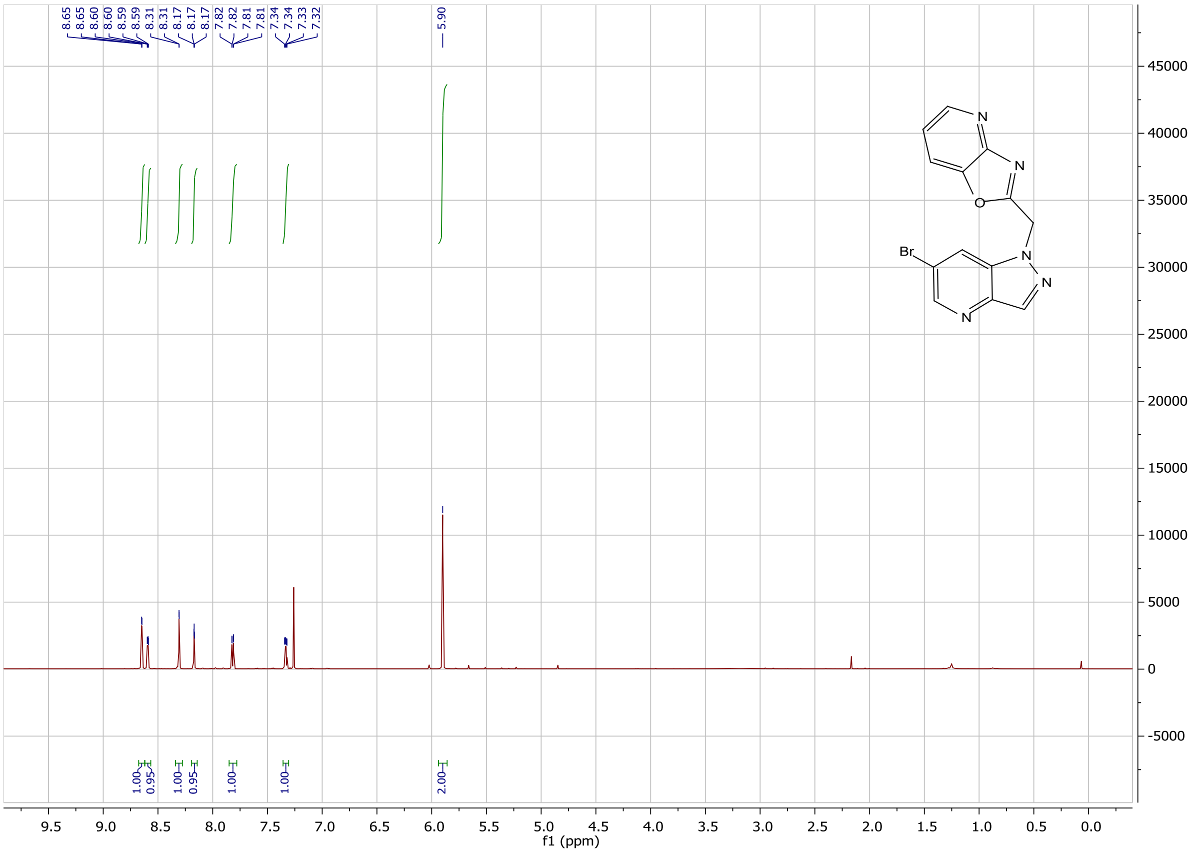

Figure S15: ^{1}H NMR for compound **24**.

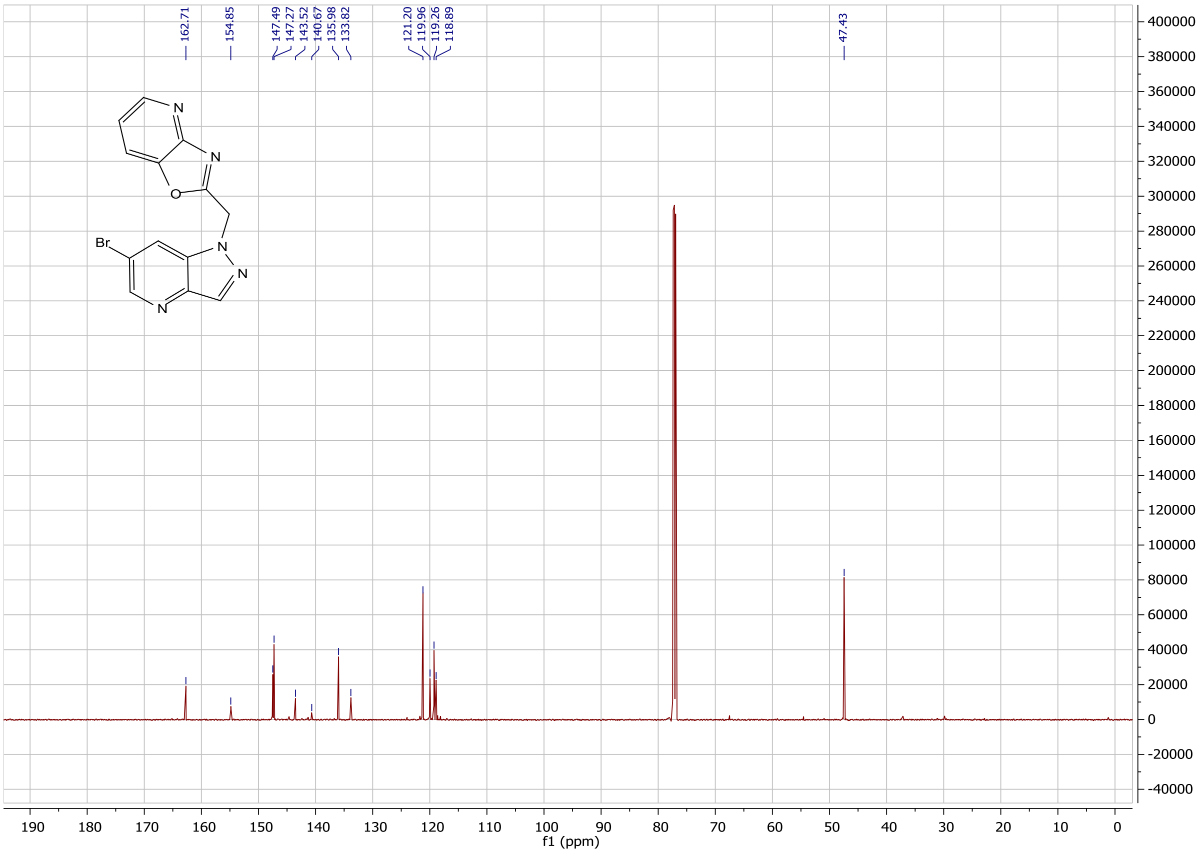

Figure S16: ^{13}C NMR for compound **24**.

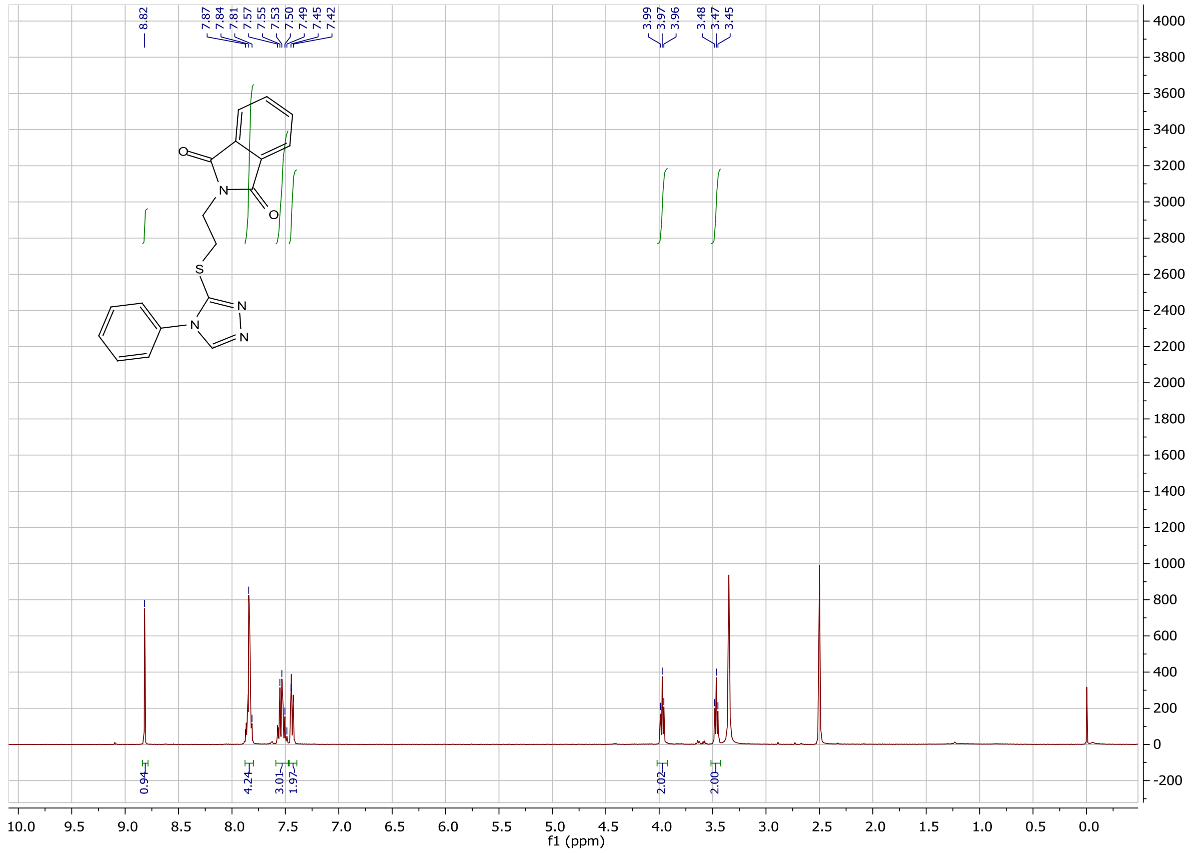

Figure S17: ^{1}H NMR for compound **25**.

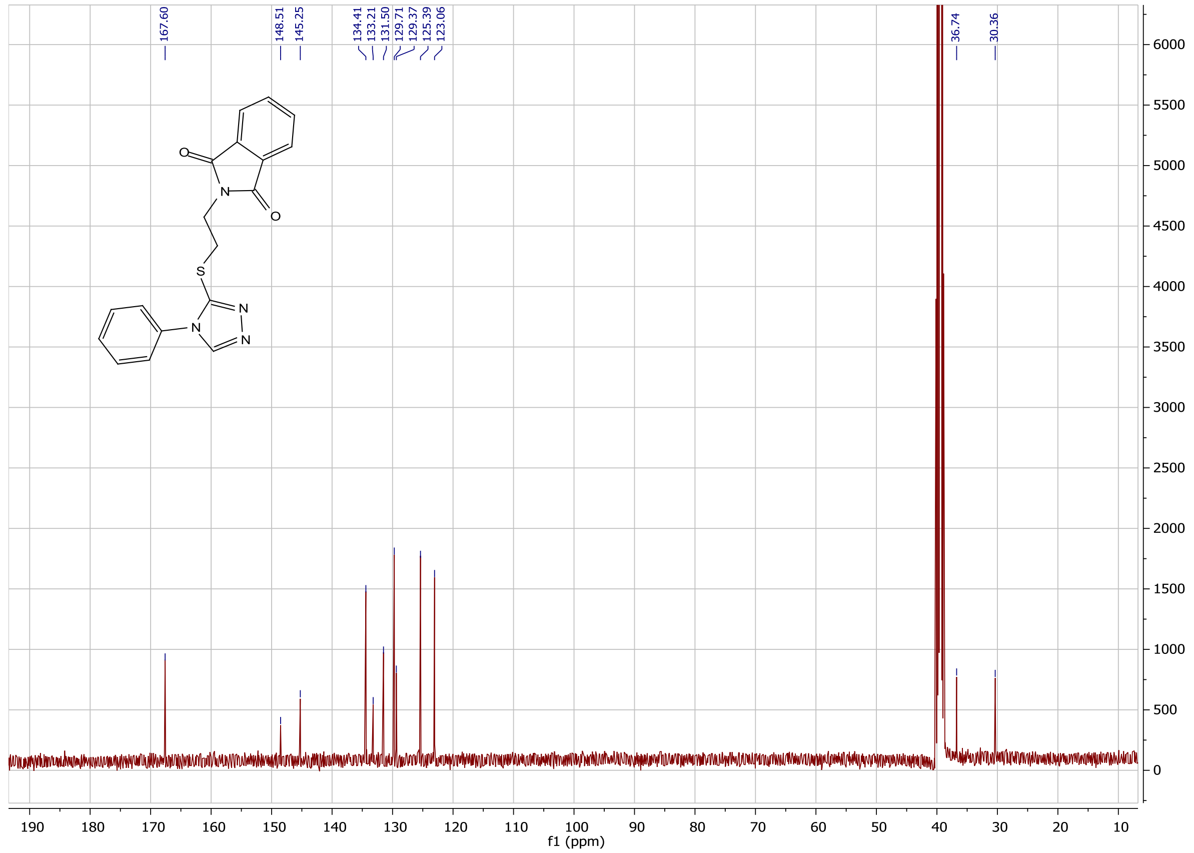

Figure S18: ^{13}C NMR for compound **25**.

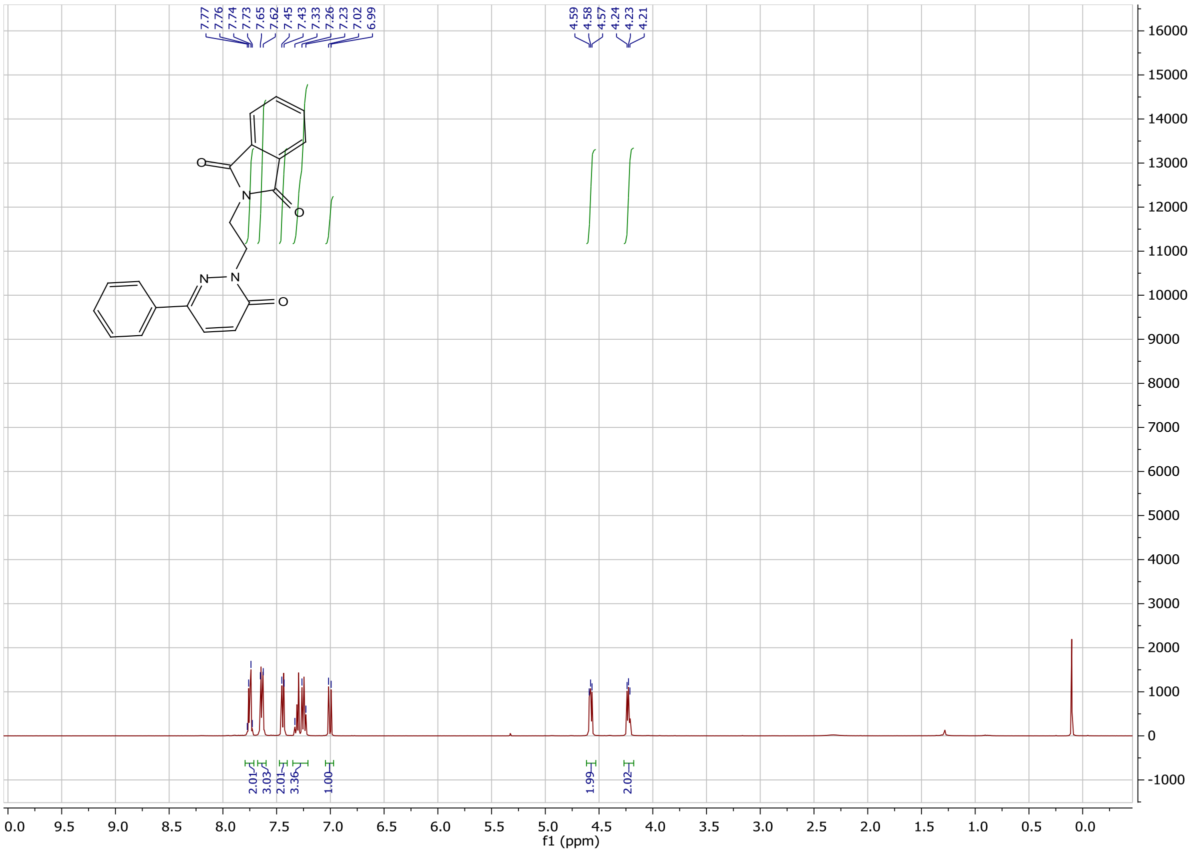

Figure S19: ^{1}H NMR for compound **26**.

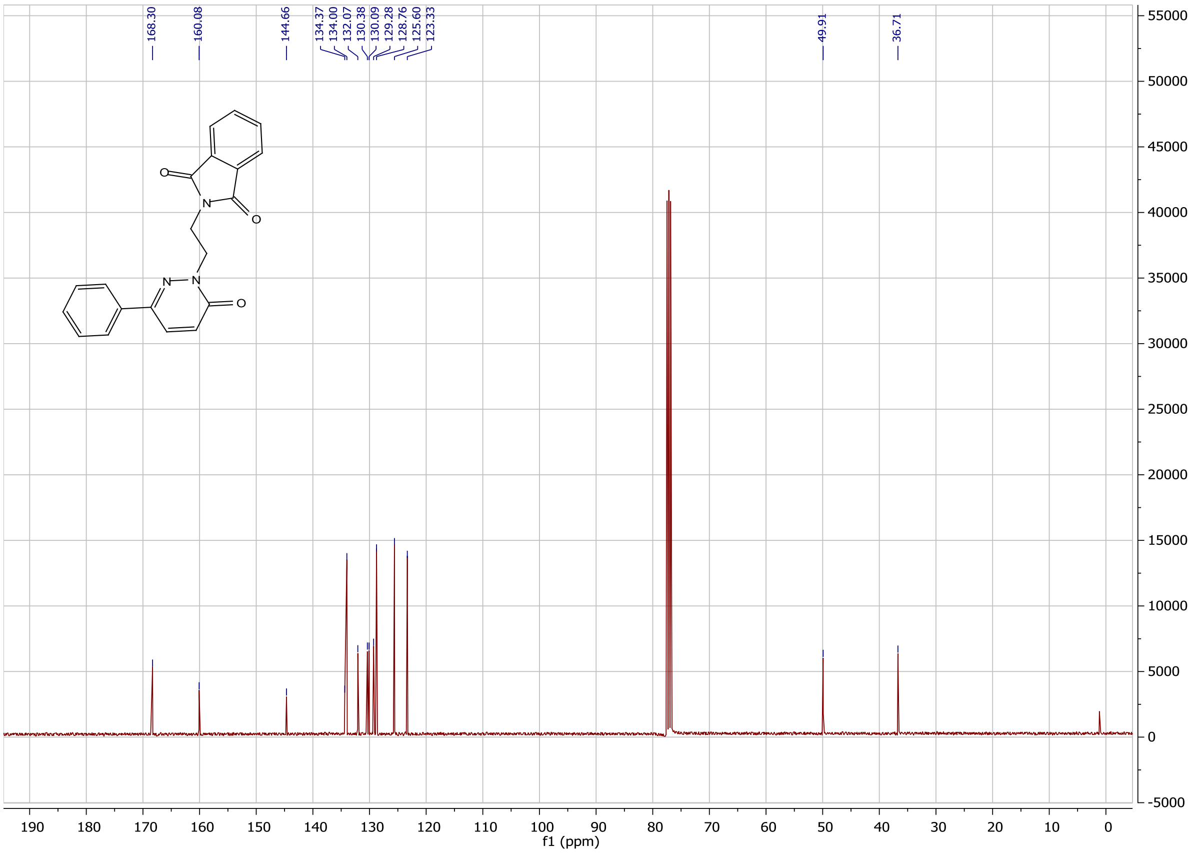

Figure S20: ^{13}C NMR for compound **26**.

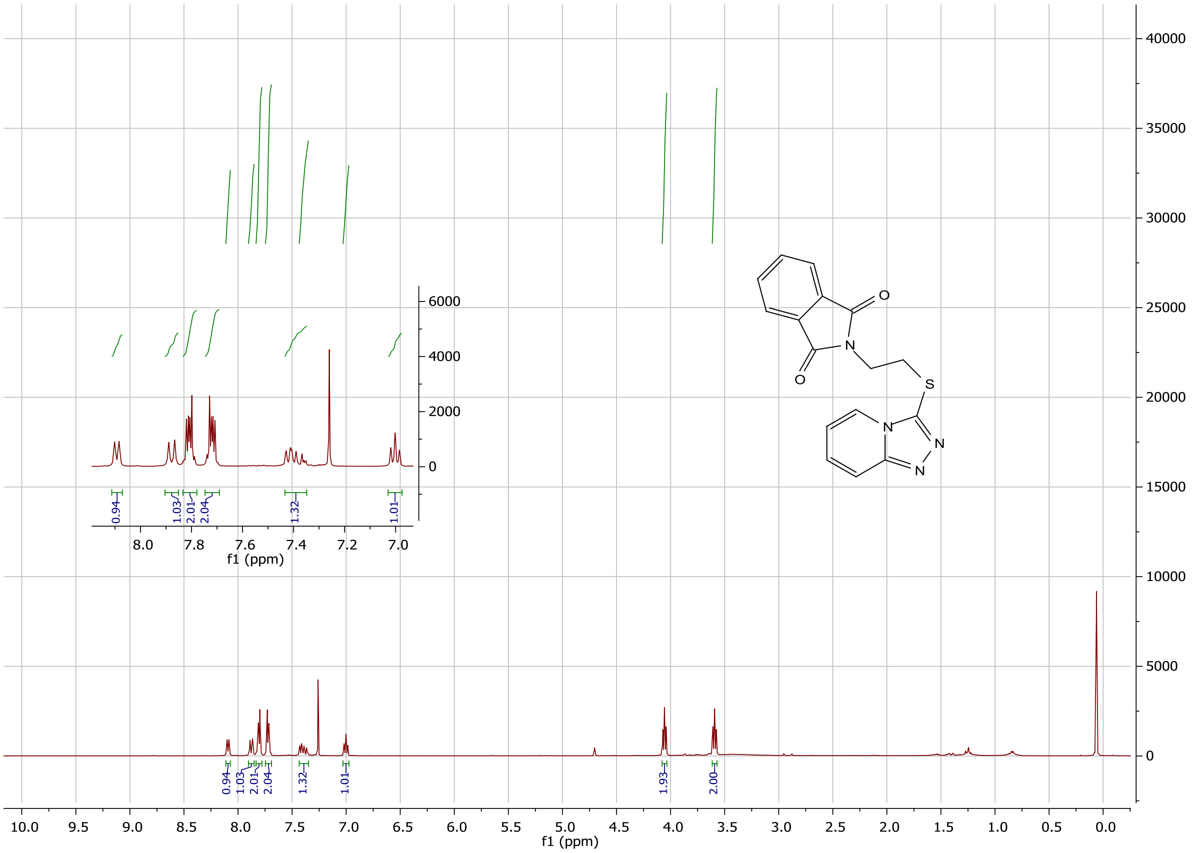

Figure S21: ^{1}H NMR for compound **27**.

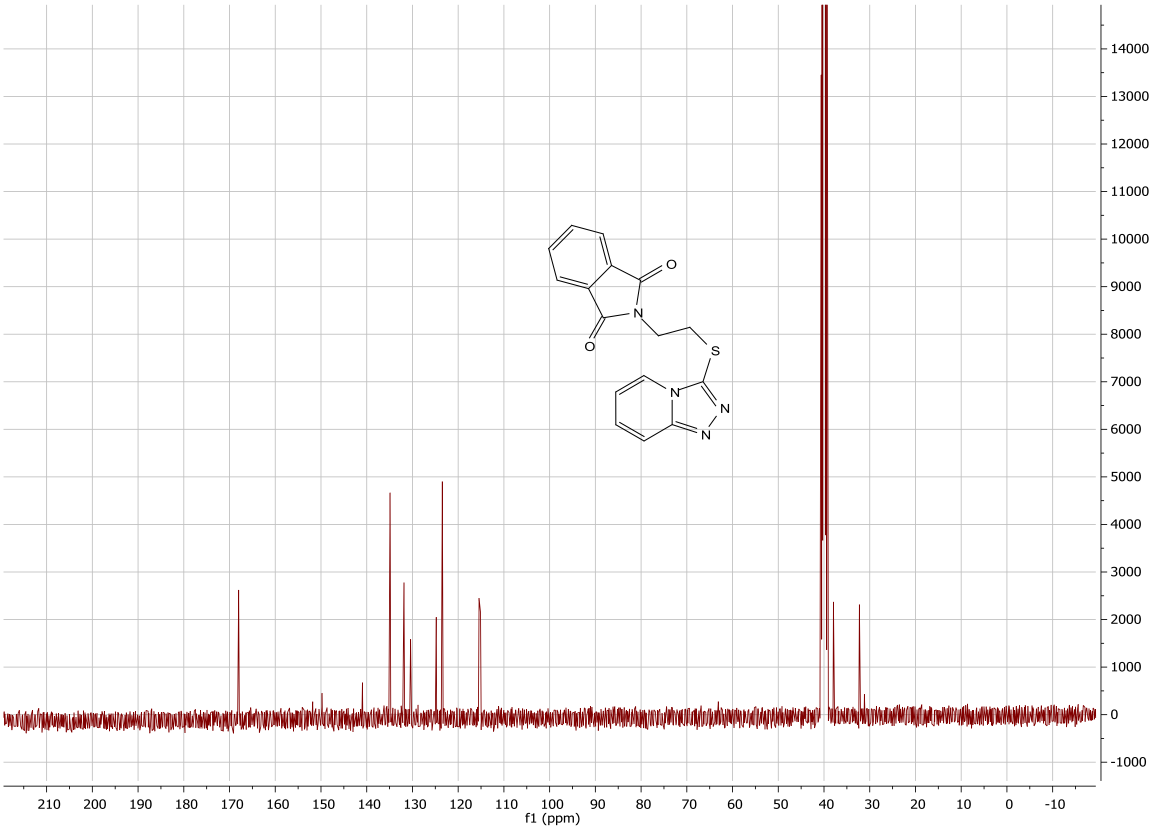

Figure S221: ^{13}C NMR for compound **27**.

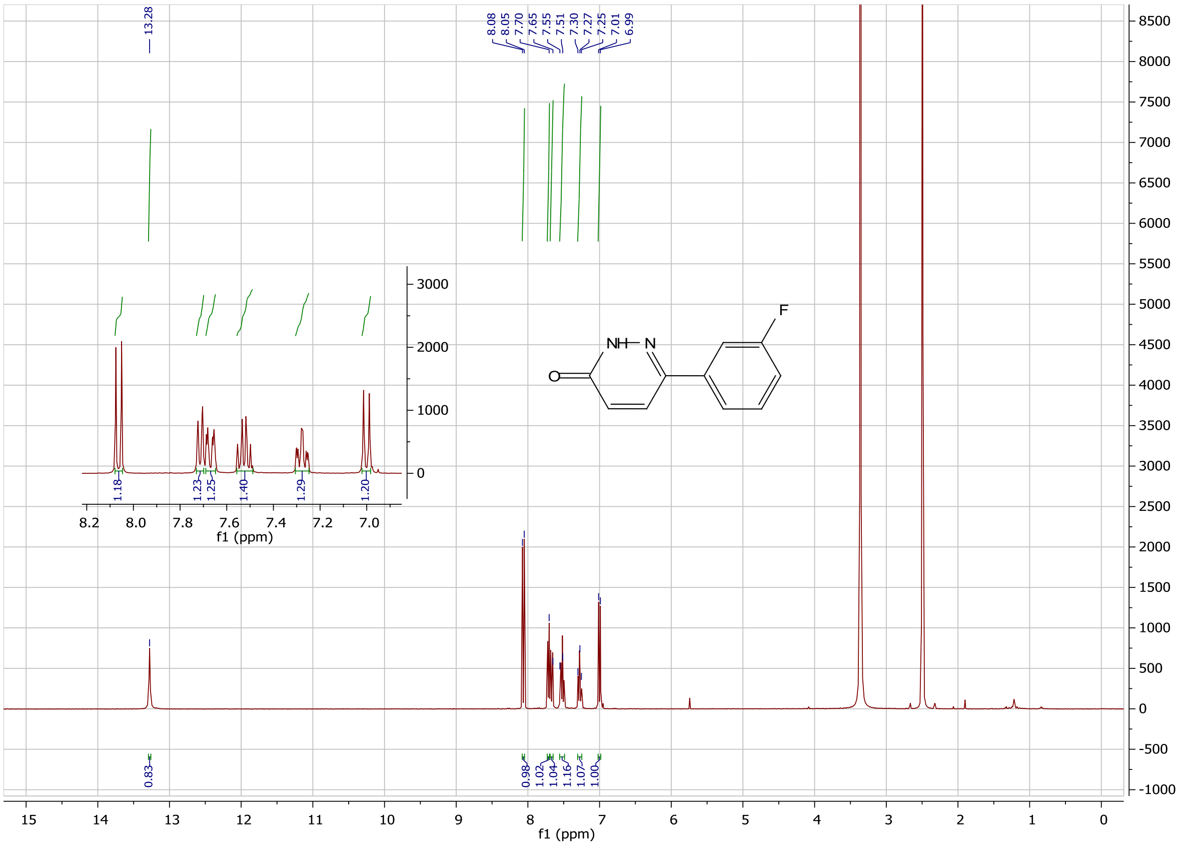

Figure S23: ^{1}H NMR for compound **29a**.

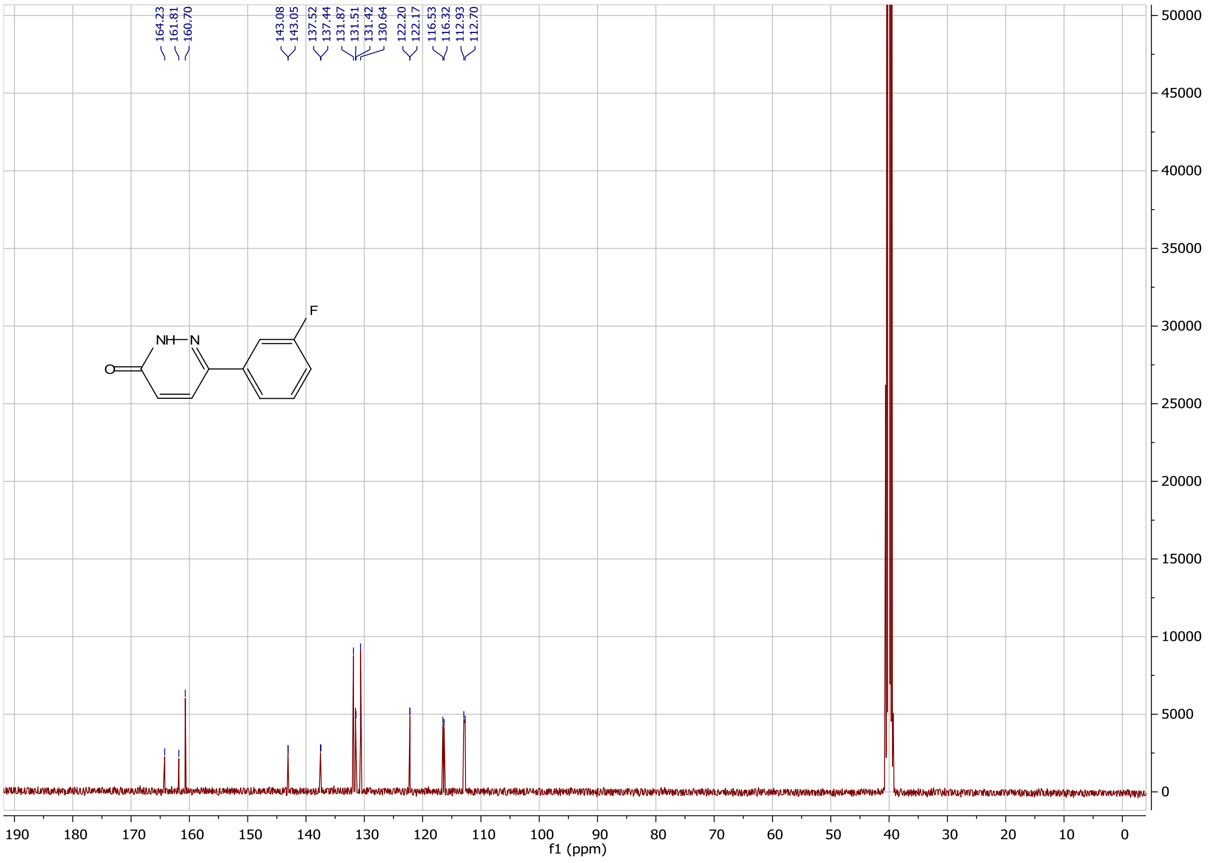

Figure S24: ^{13}C NMR for compound **29a**.

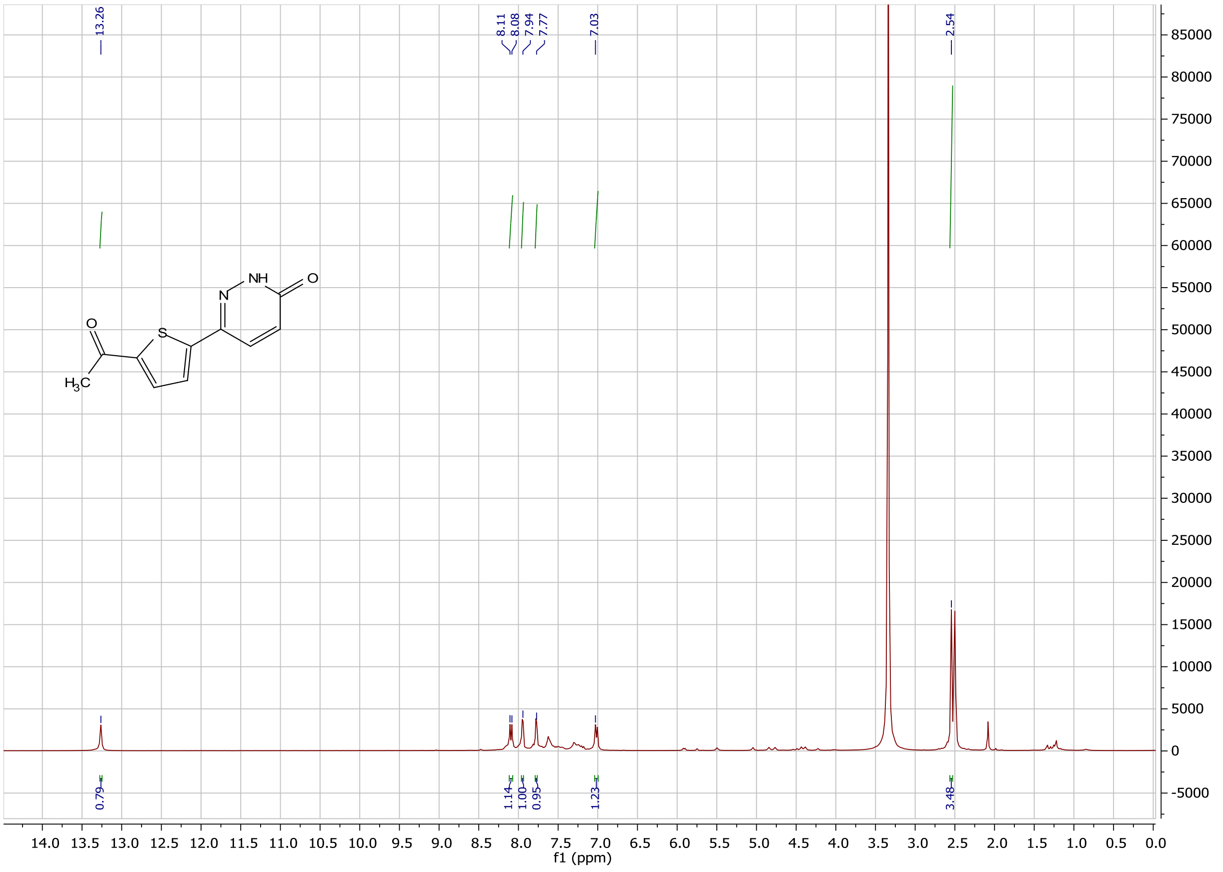

Figure S25: ^{1}H NMR for compound **29c**.

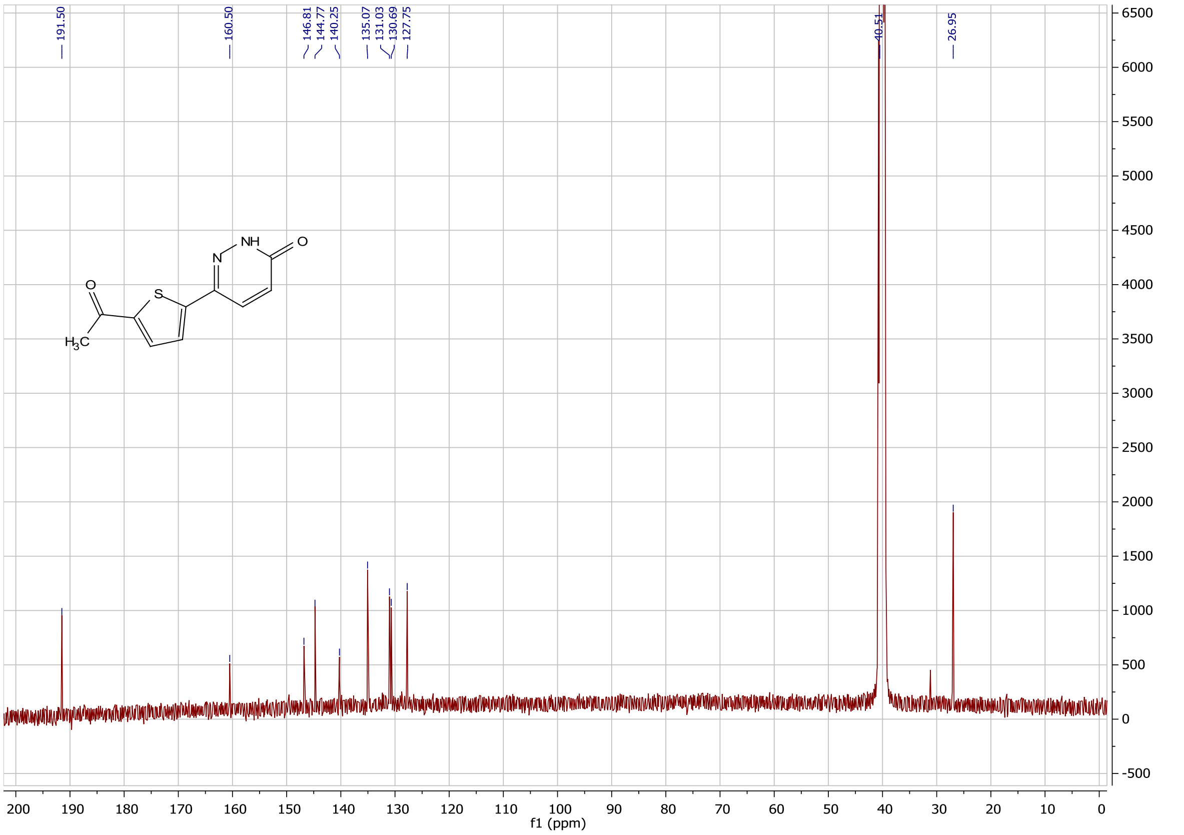

Figure S26: ^{13}C NMR for compound **29c**.

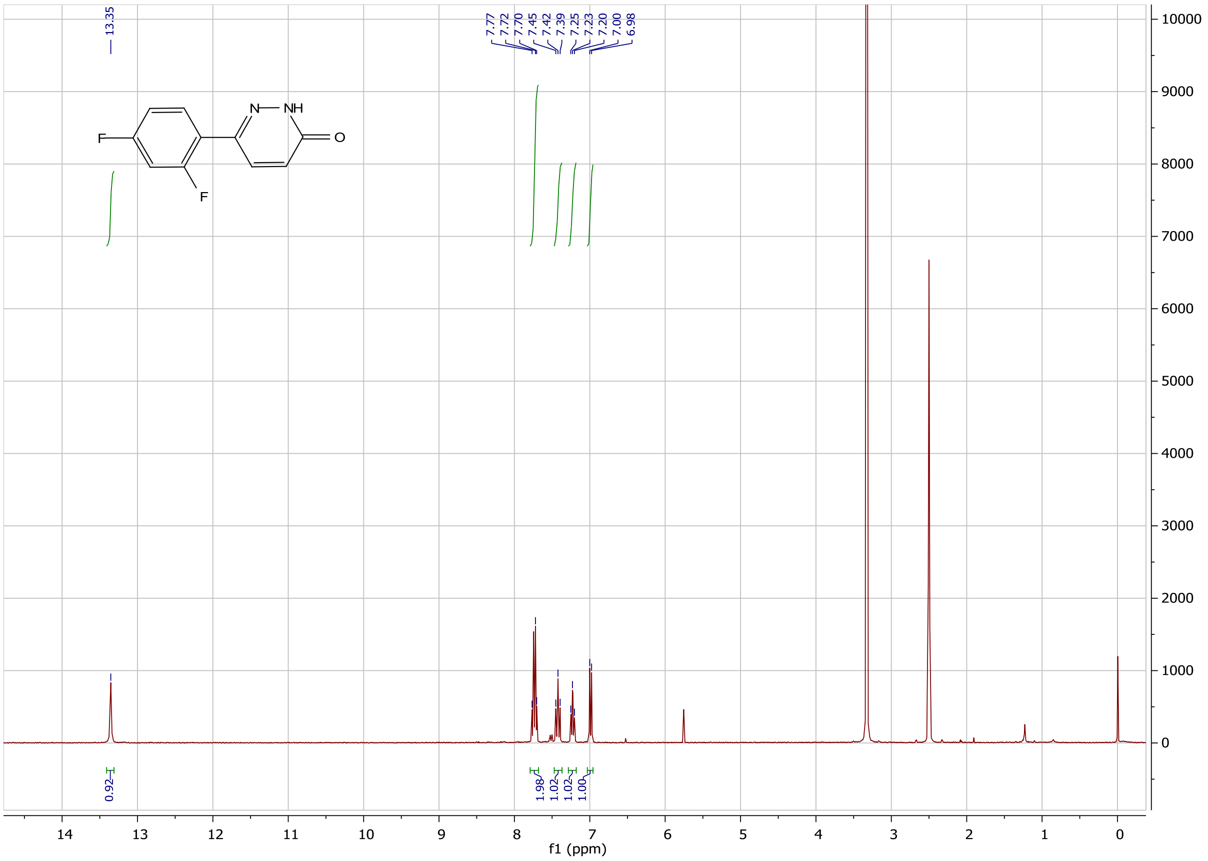

Figure S27: ^{1}H NMR for compound **29d**.

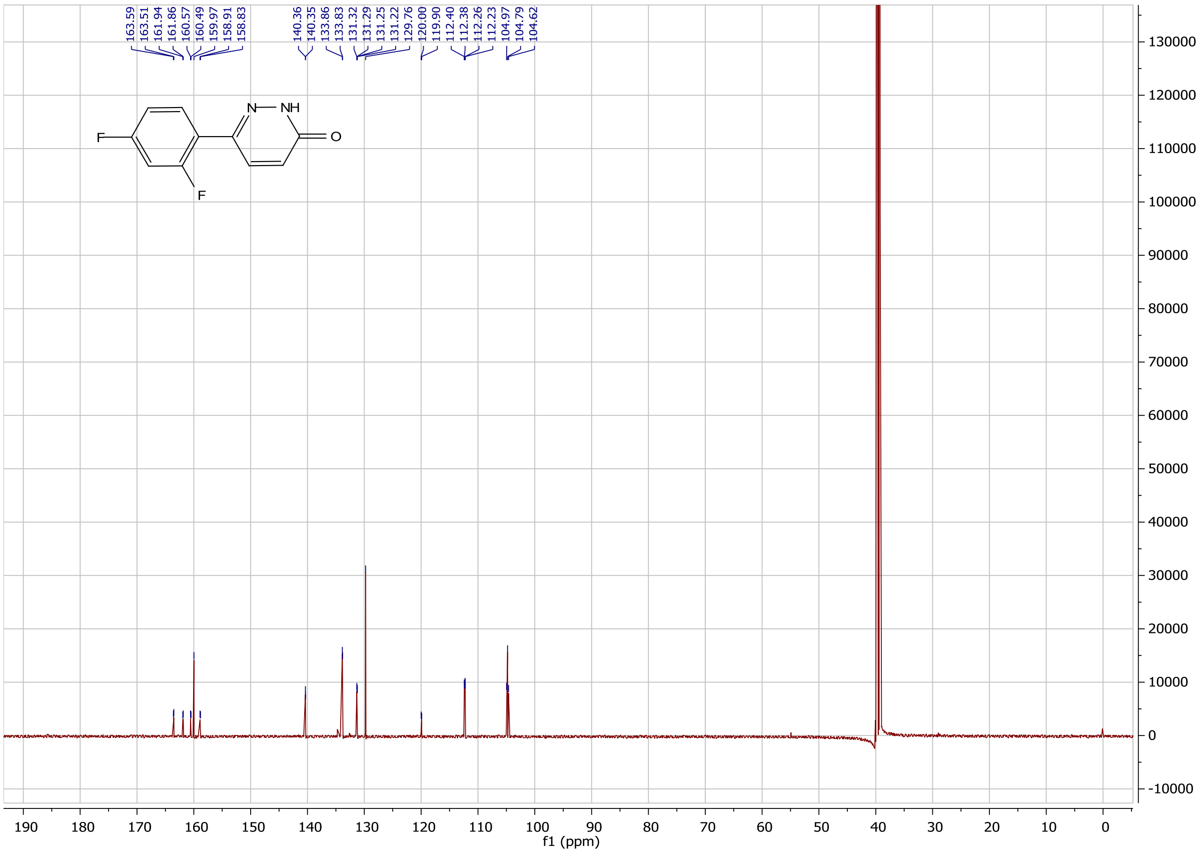

Figure S28: ^{13}C NMR for compound **29d**.

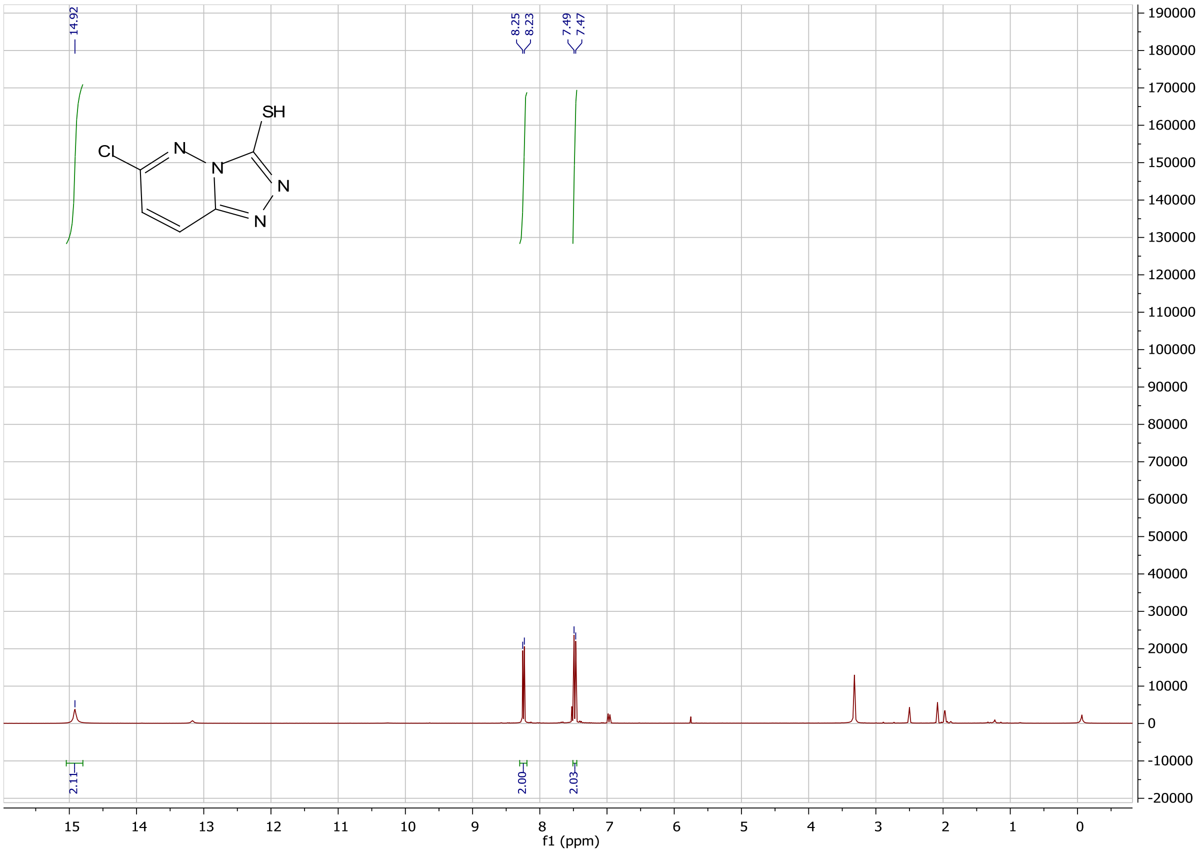

Figure S29: ^{1}H NMR for compound **31a**.

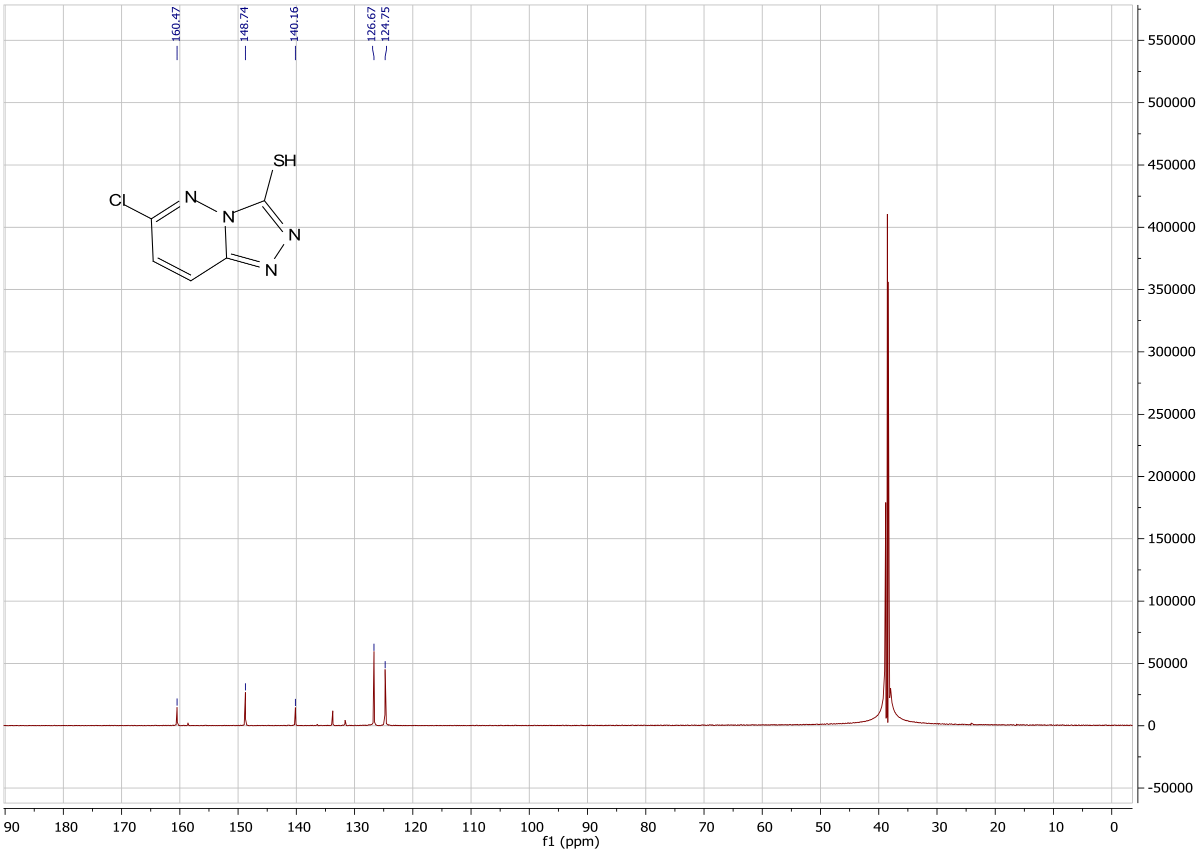

Figure S30: ^{13}C NMR for compound **31a**.

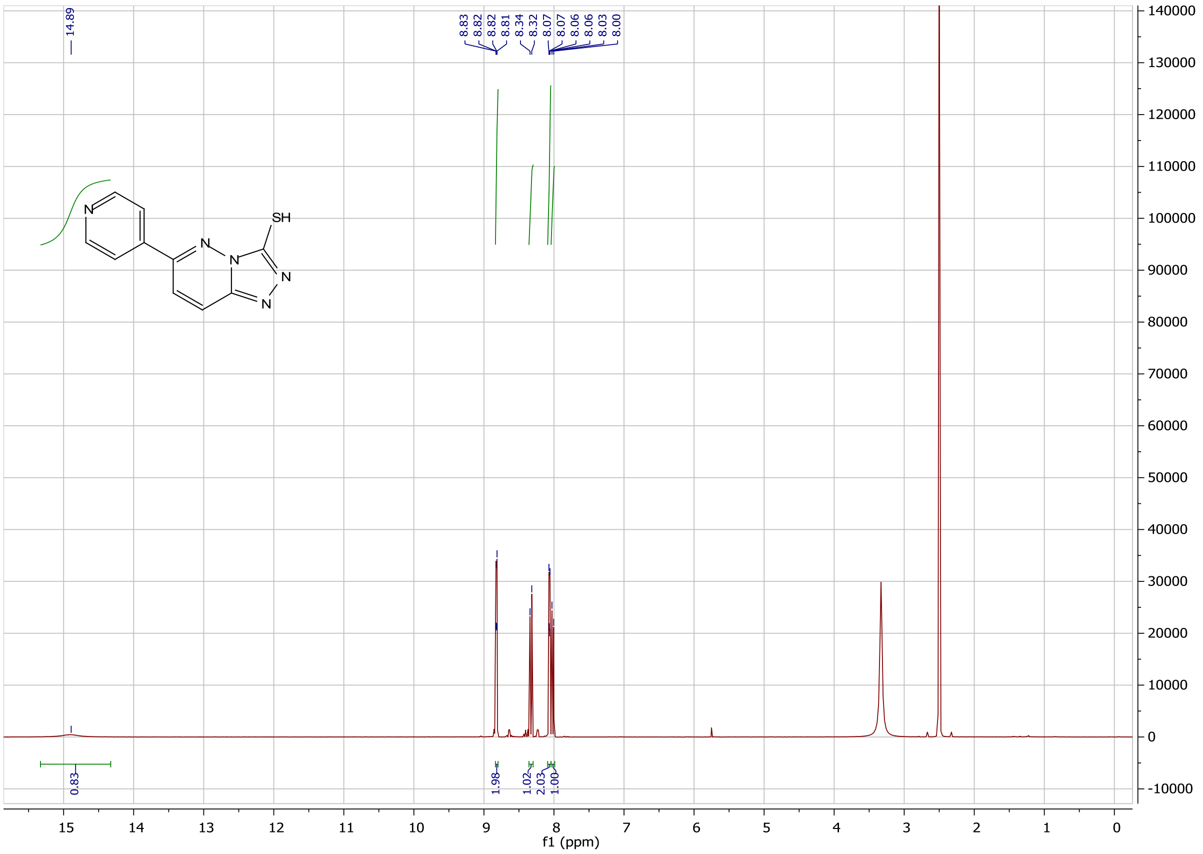

Figure S31: ^{1}H NMR for compound **31b**.

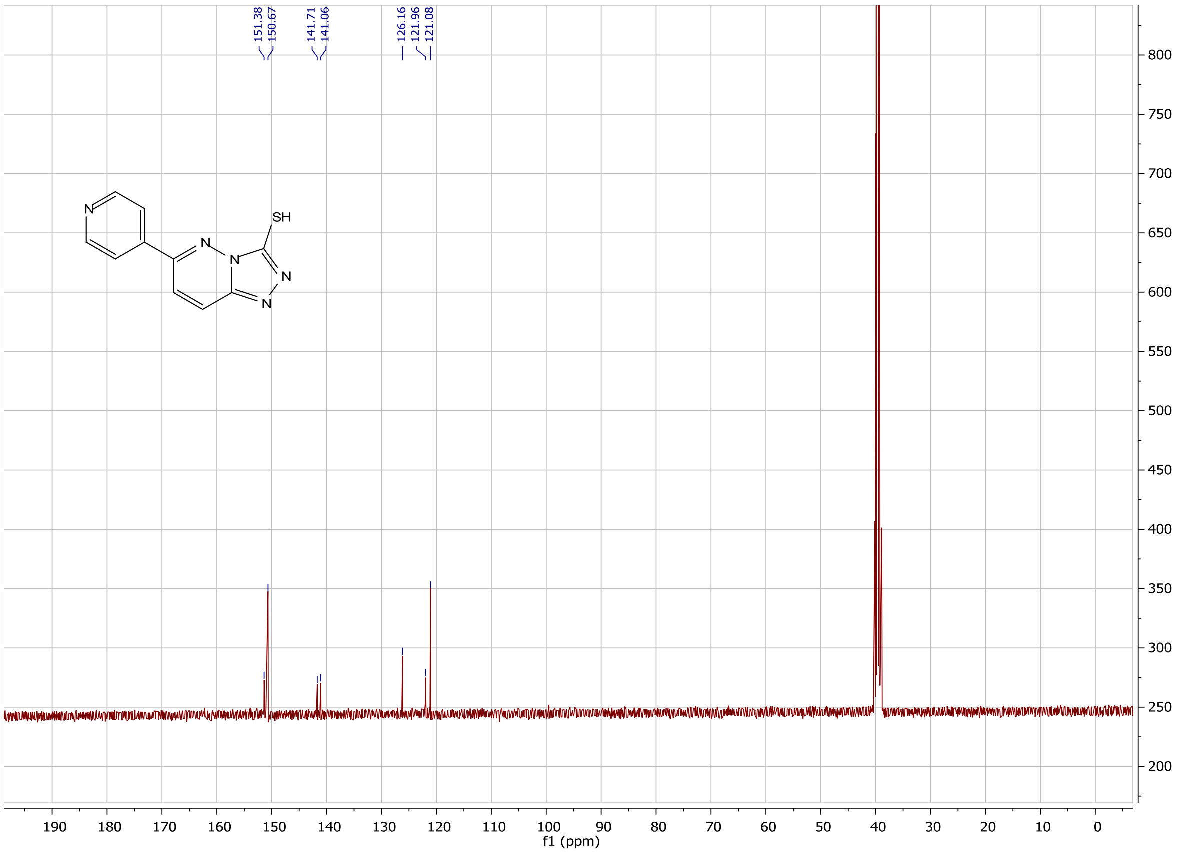

Figure S32: ^{13}C NMR for compound **31b**.

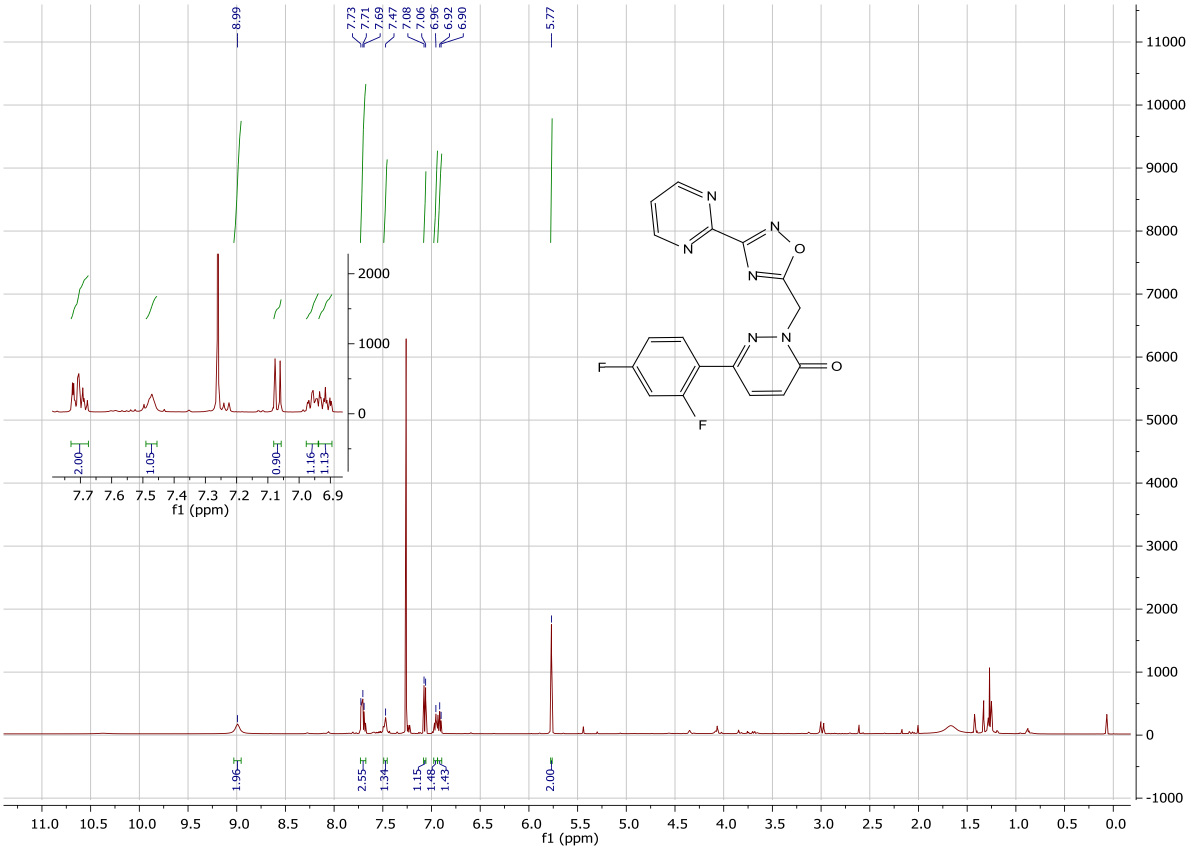

Figure S33: ^{1}H NMR for compound **34**.

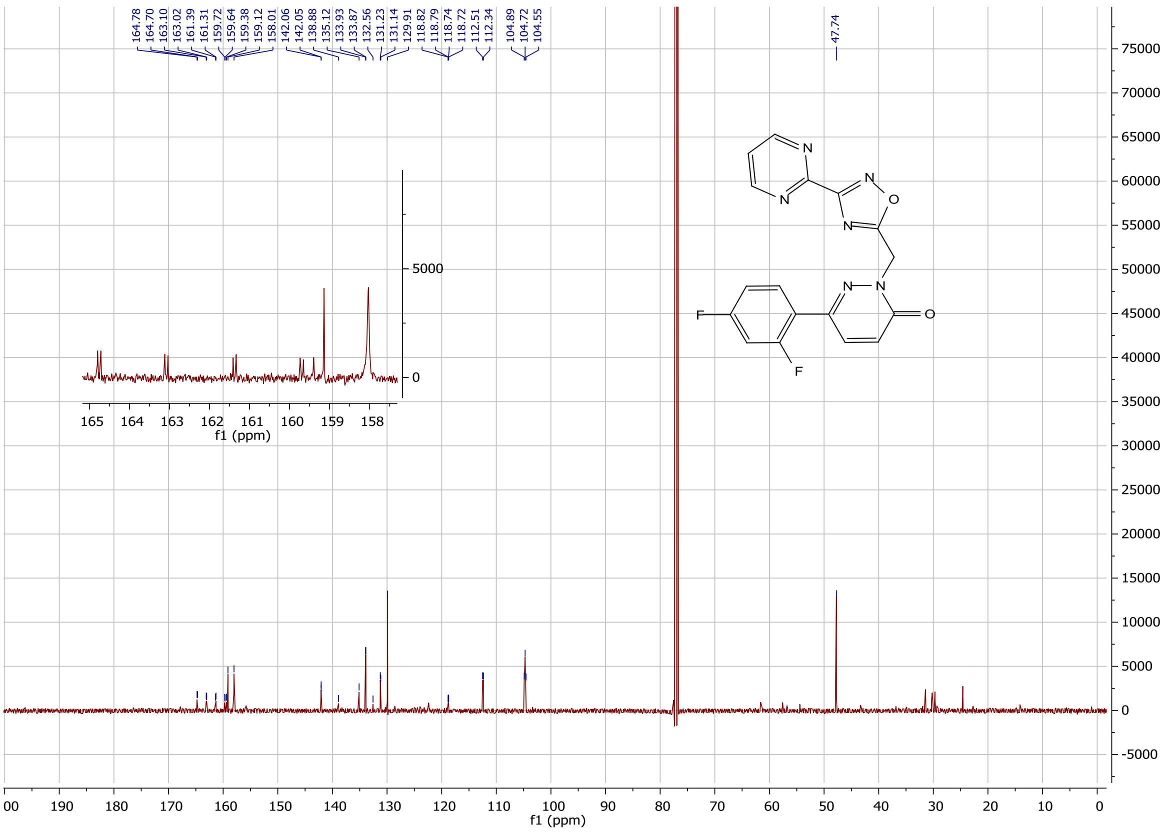

Figure S34: ^{13}C NMR for compound **34**.

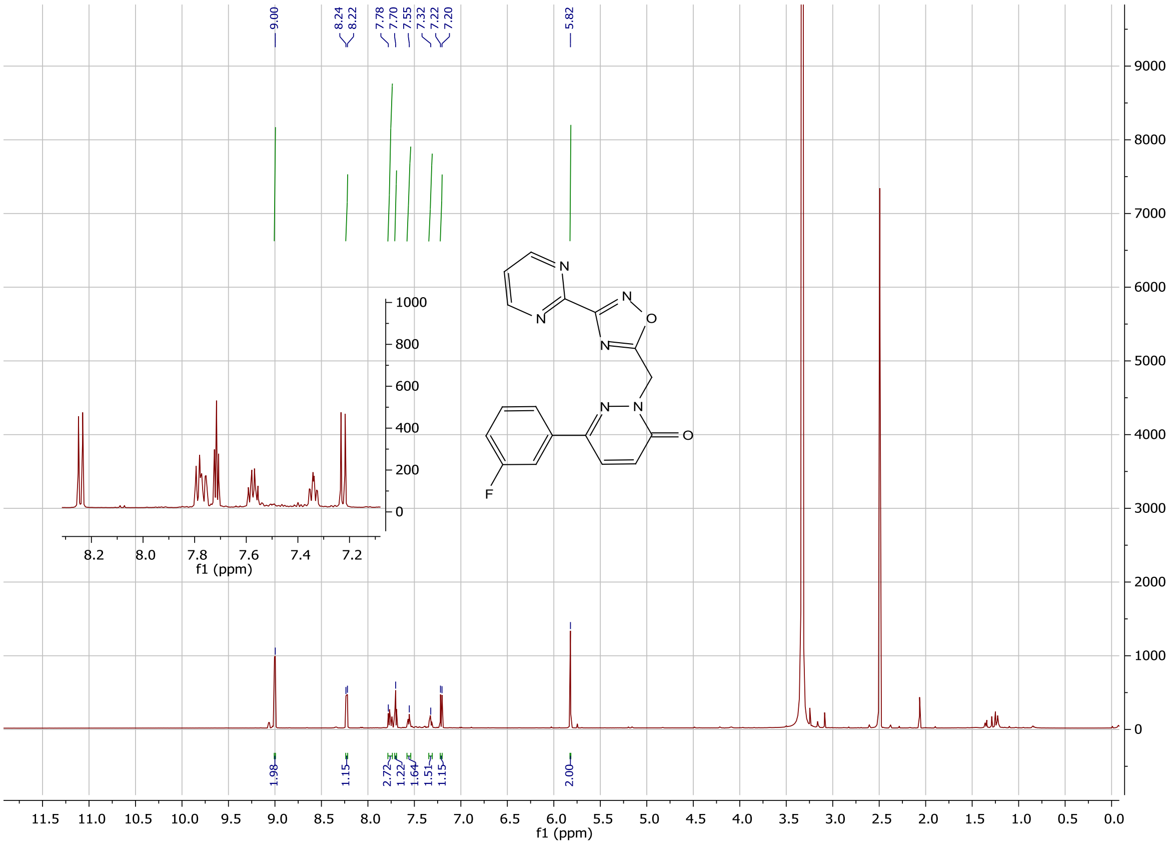

Figure S35: ^{1}H NMR for compound **35**.

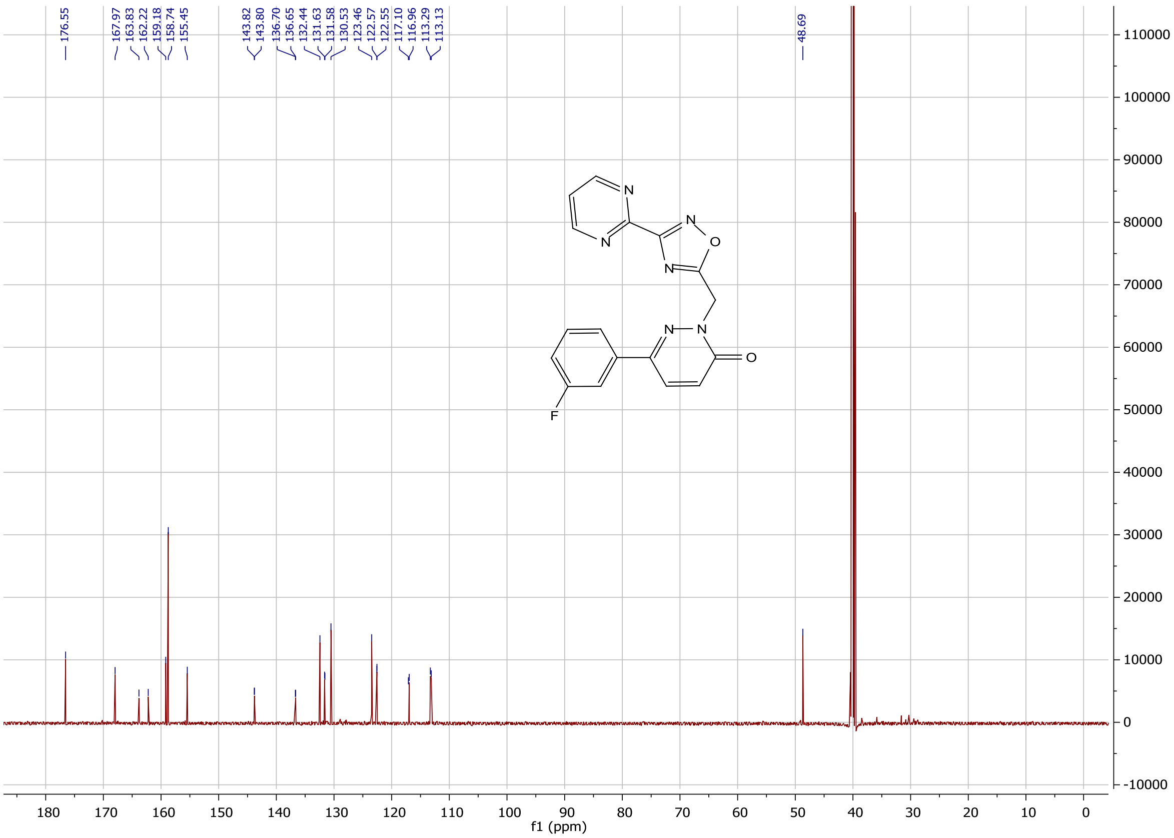

Figure S36: ^{13}C NMR for compound **35**.

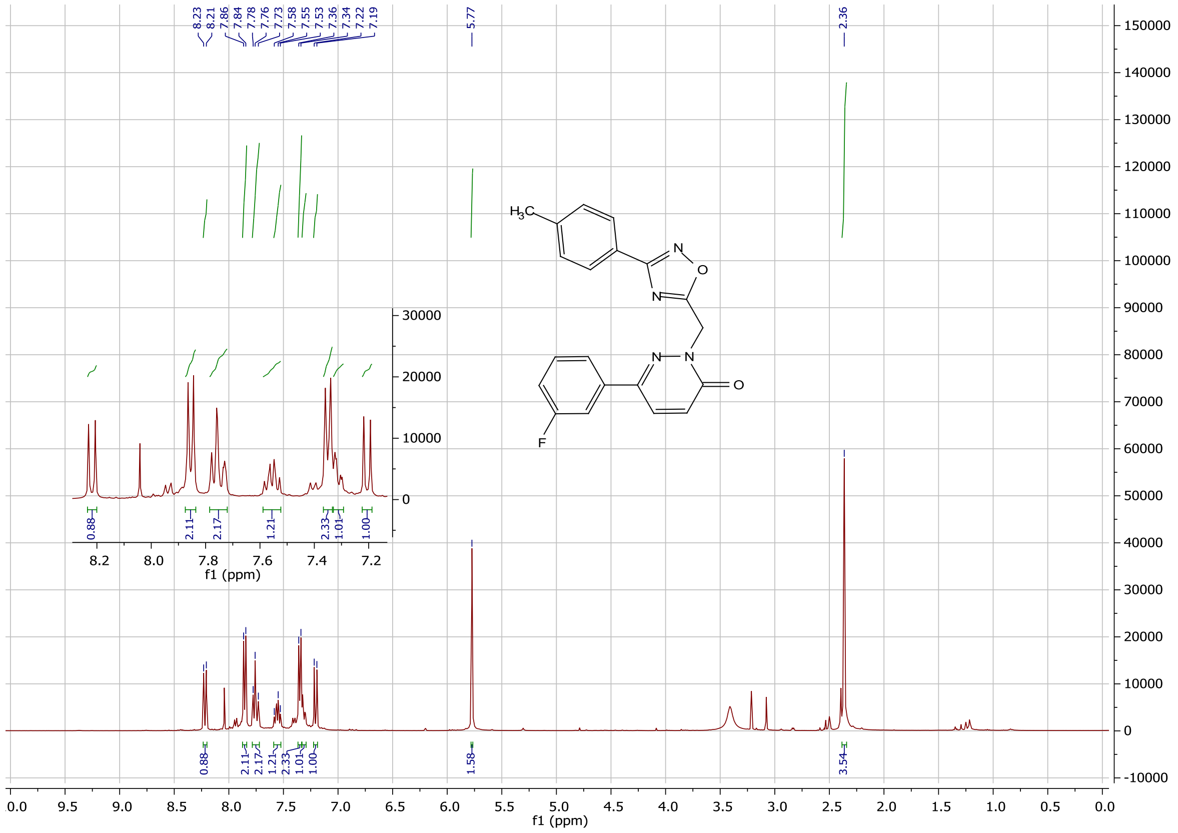

Figure S37: ^{1}H NMR for compound **36**.

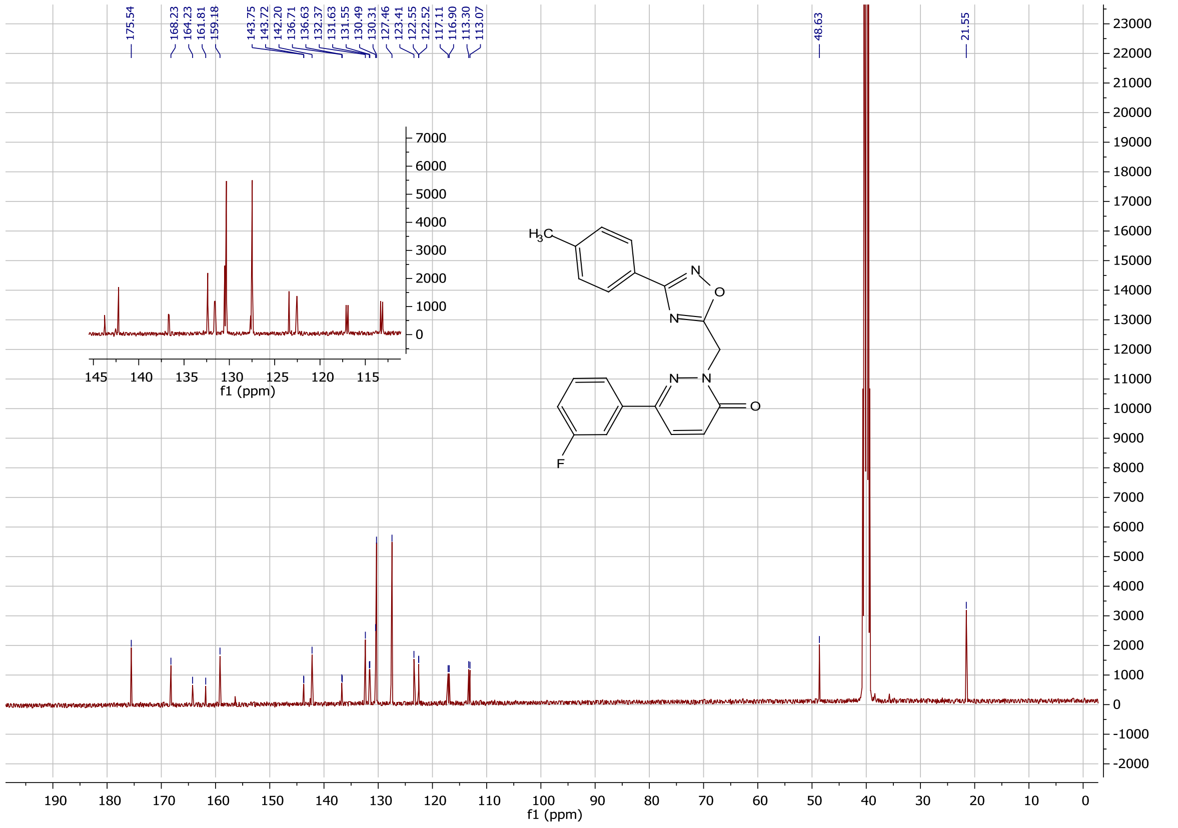

Figure S38: ^{13}C NMR for compound **36**.

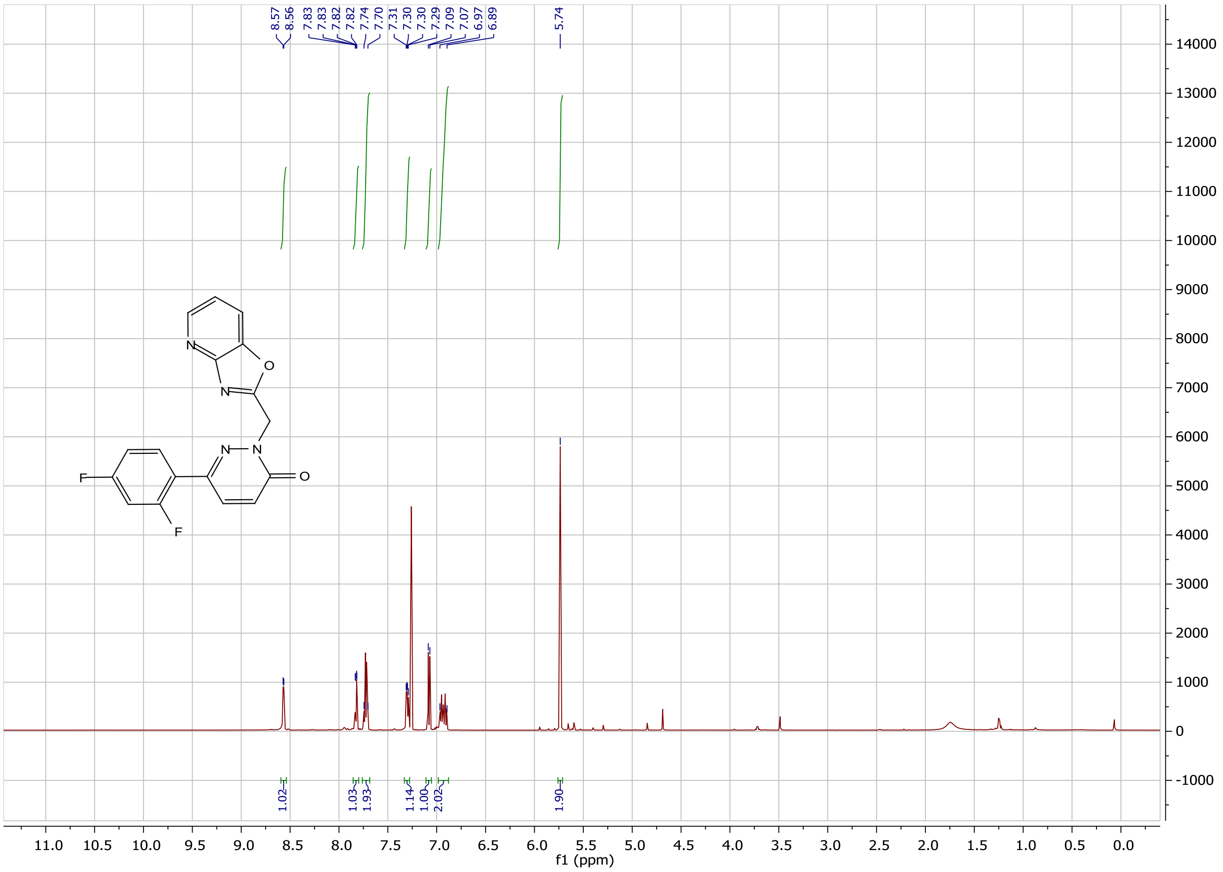

Figure S39: ^{1}H NMR for compound **37**.

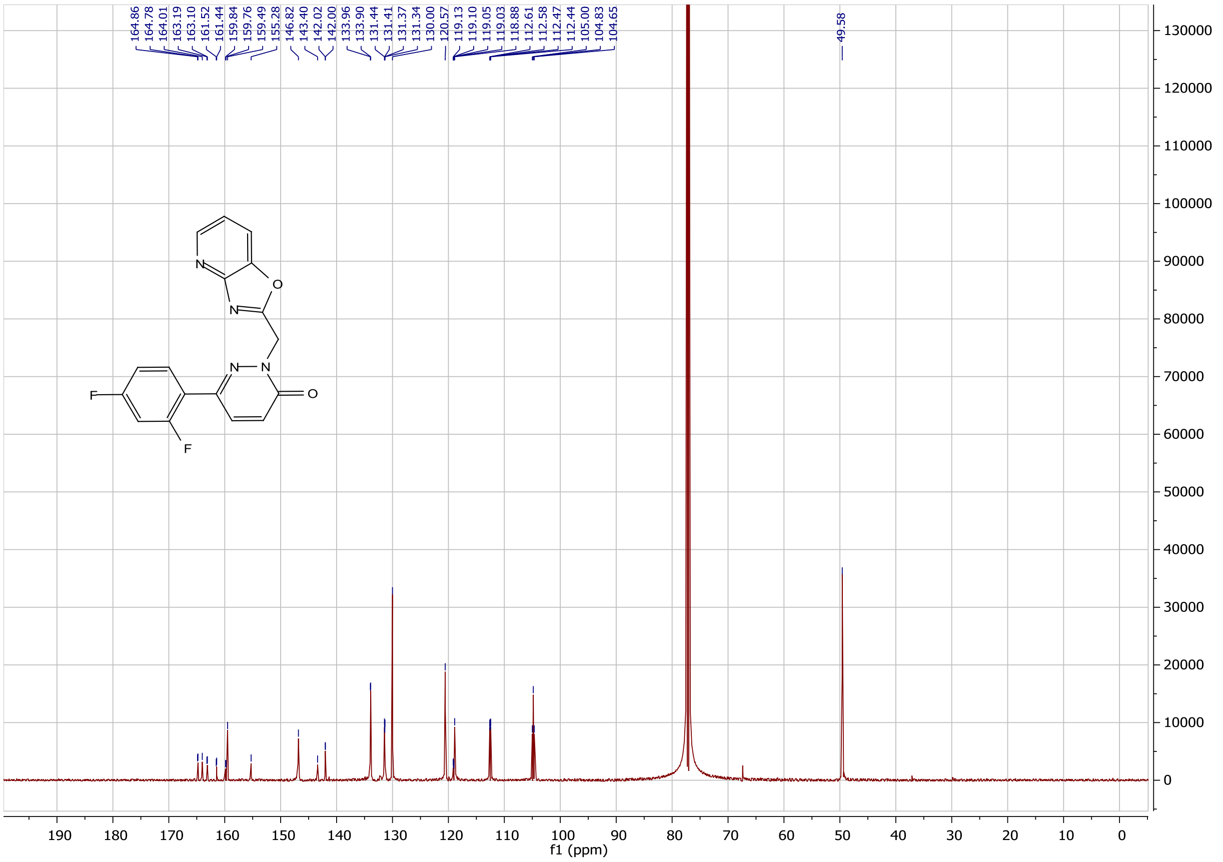

Figure S40: ^{13}C NMR for compound **37**.

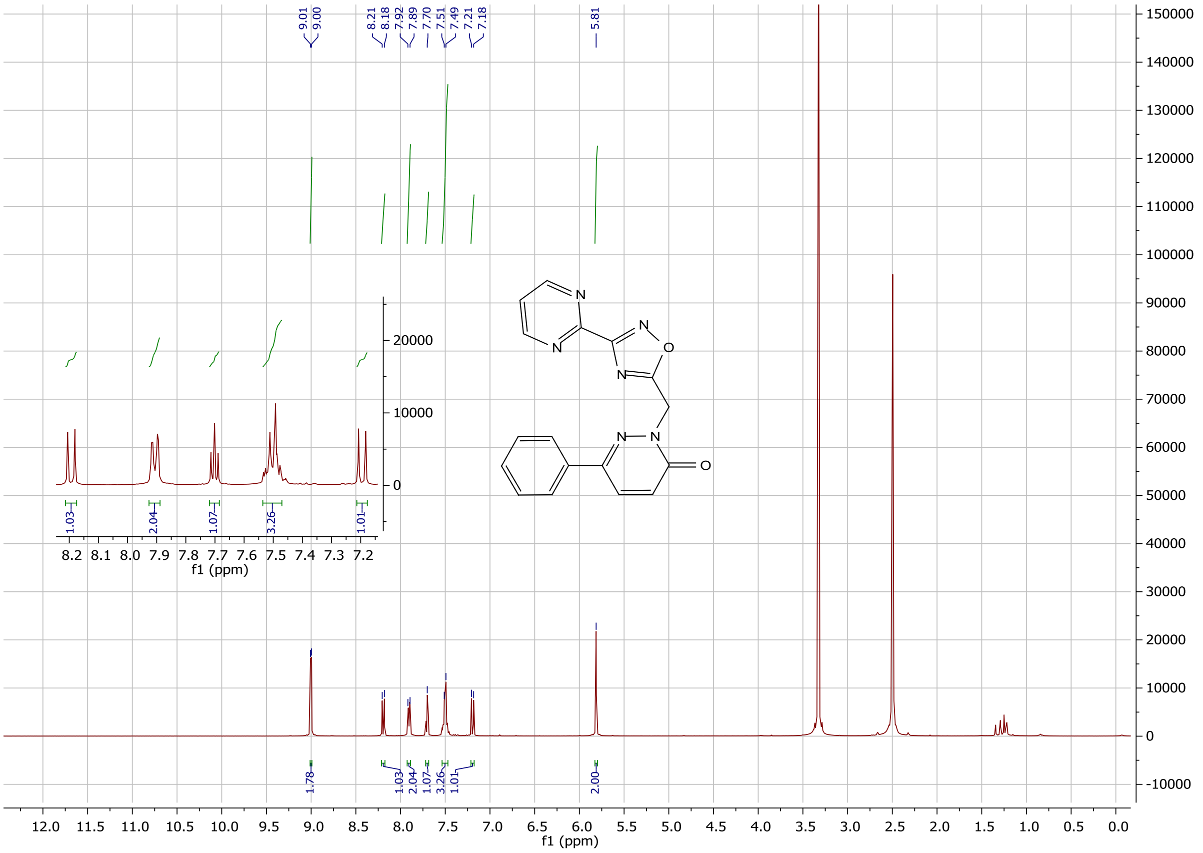

Figure S41: ^{1}H NMR for compound **38**.

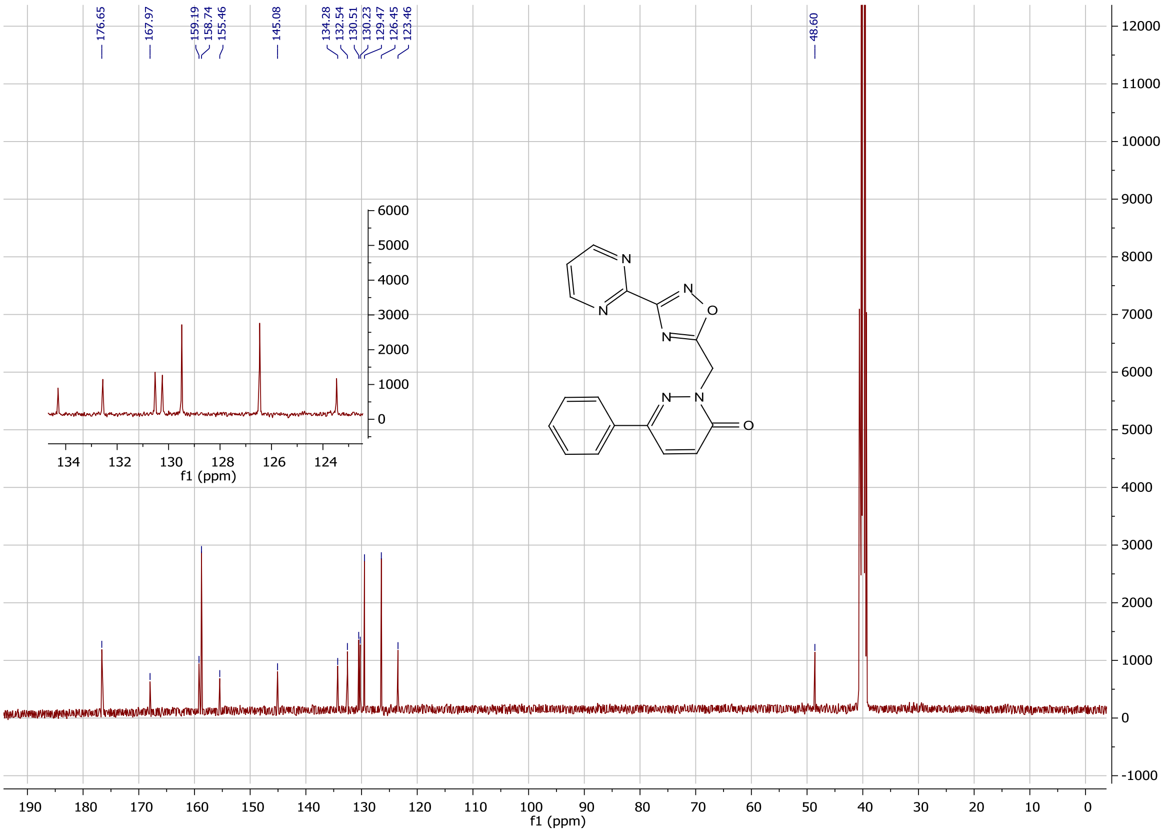

Figure S42: ^{13}C NMR for compound **38**.

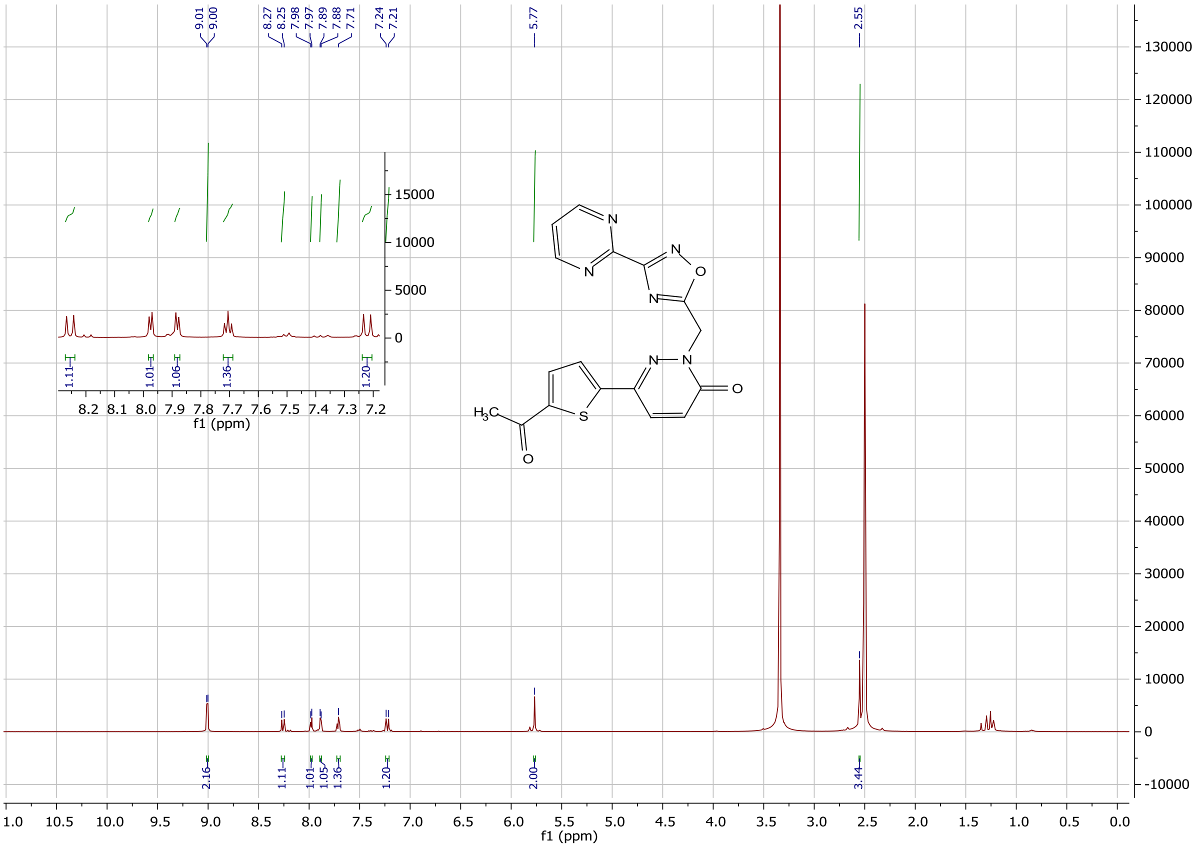

Figure S43: ^{1}H NMR for compound **40**.

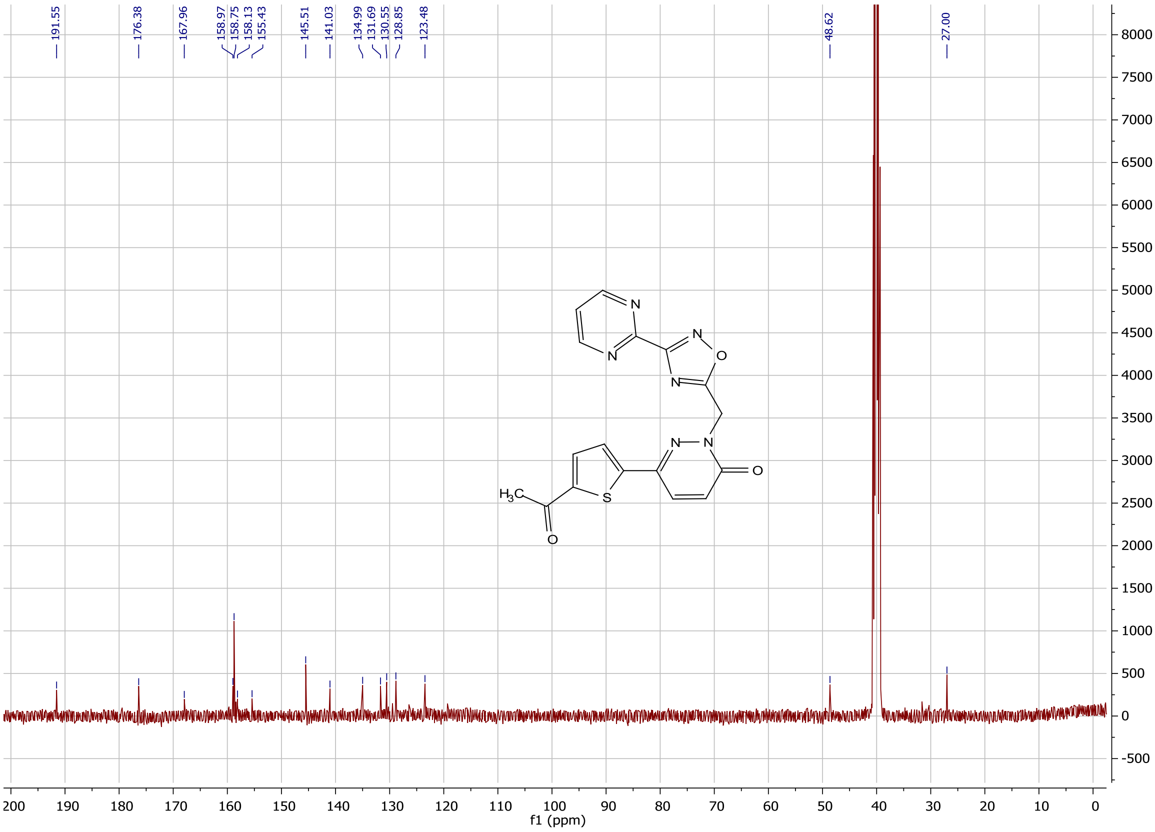

Figure S44: ^{13}C NMR for compound **40**.

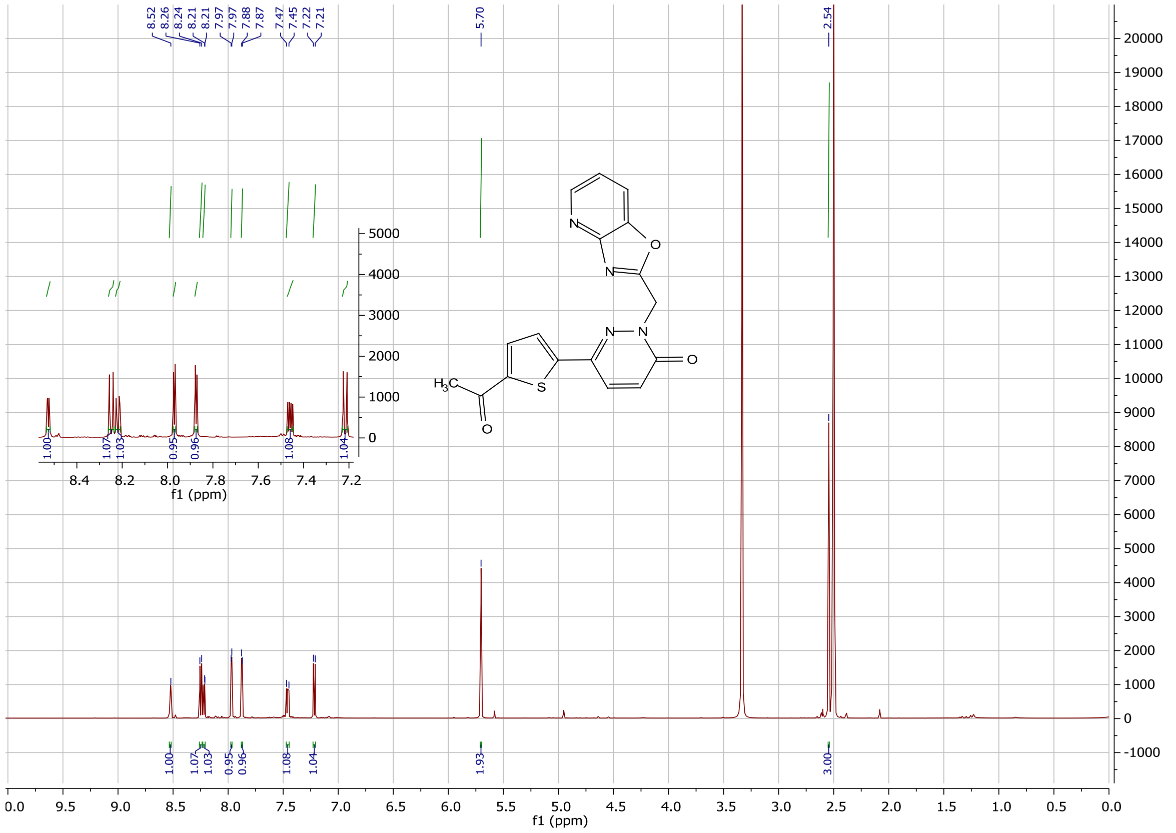

Figure S45: ^{1}H NMR for compound **41**.

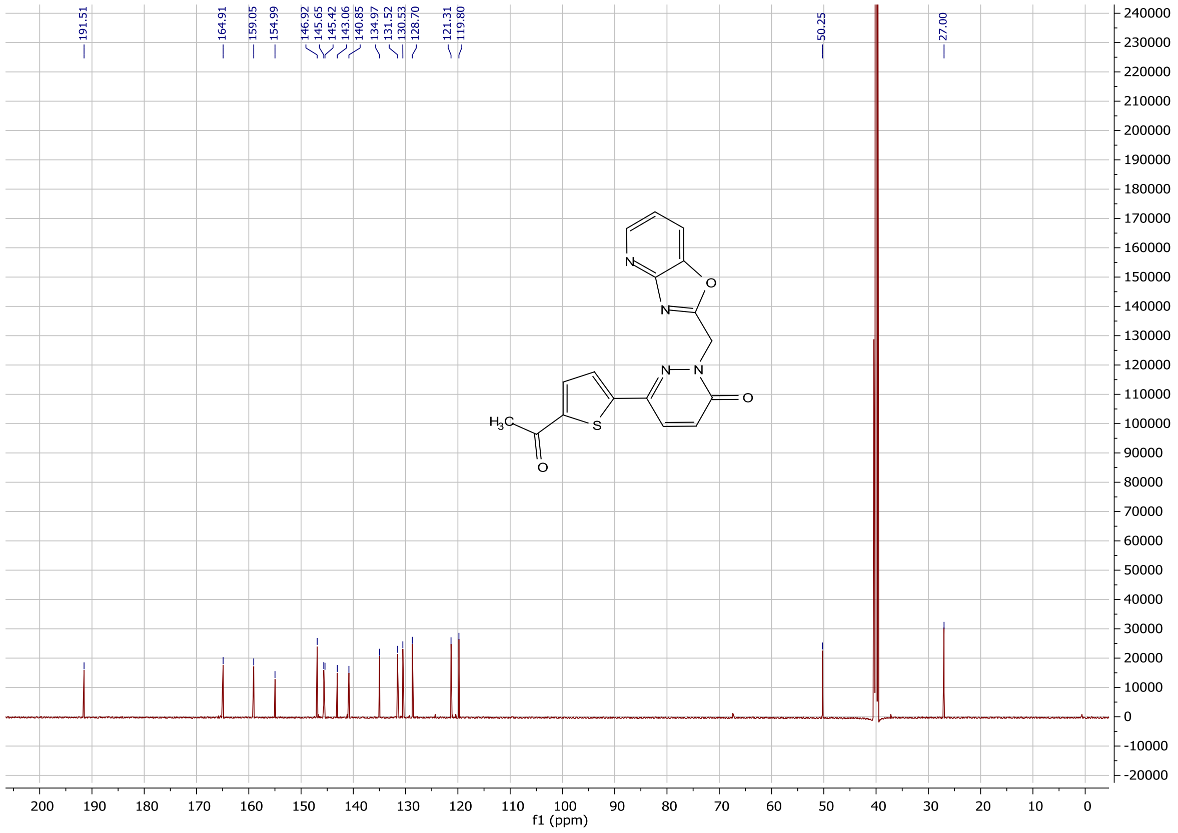

Figure S46: ^{13}C NMR for compound **41**.

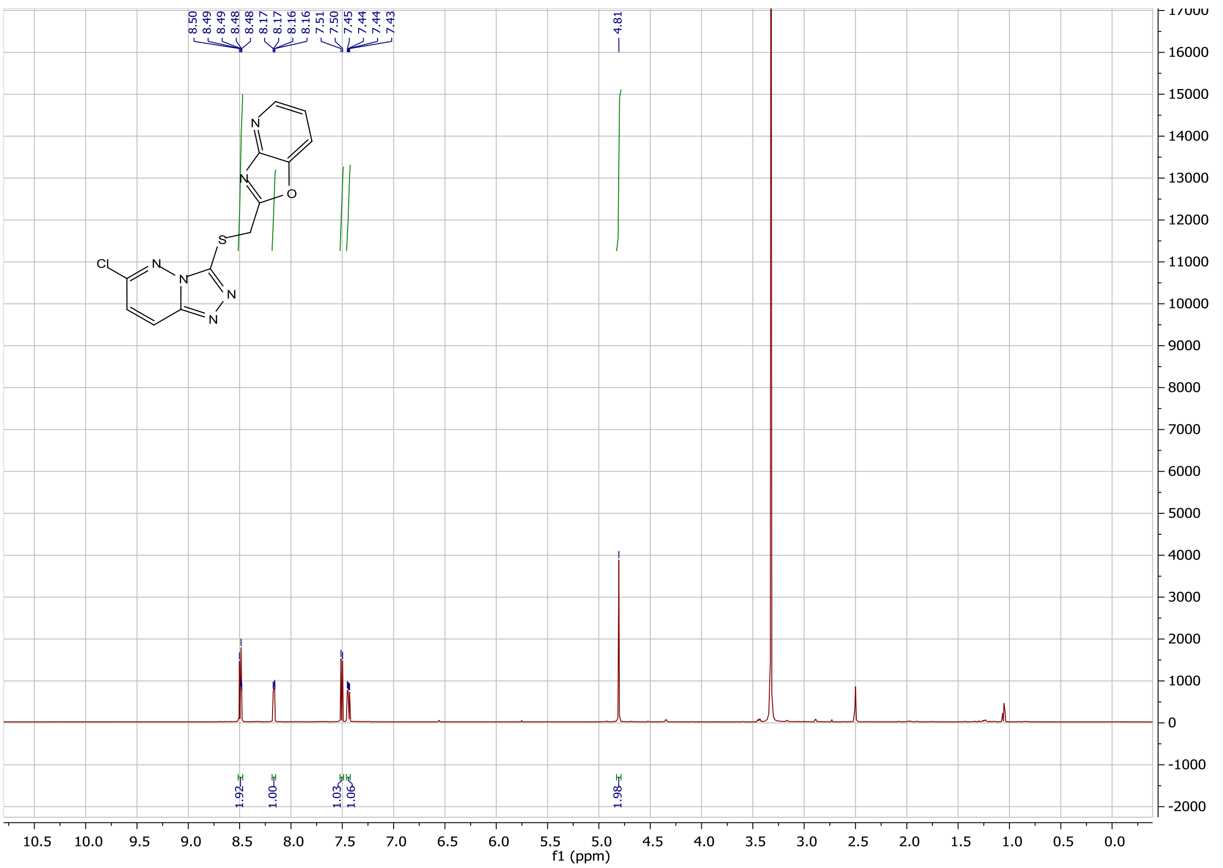

Figure S47: ^{1}H NMR for compound **42**.

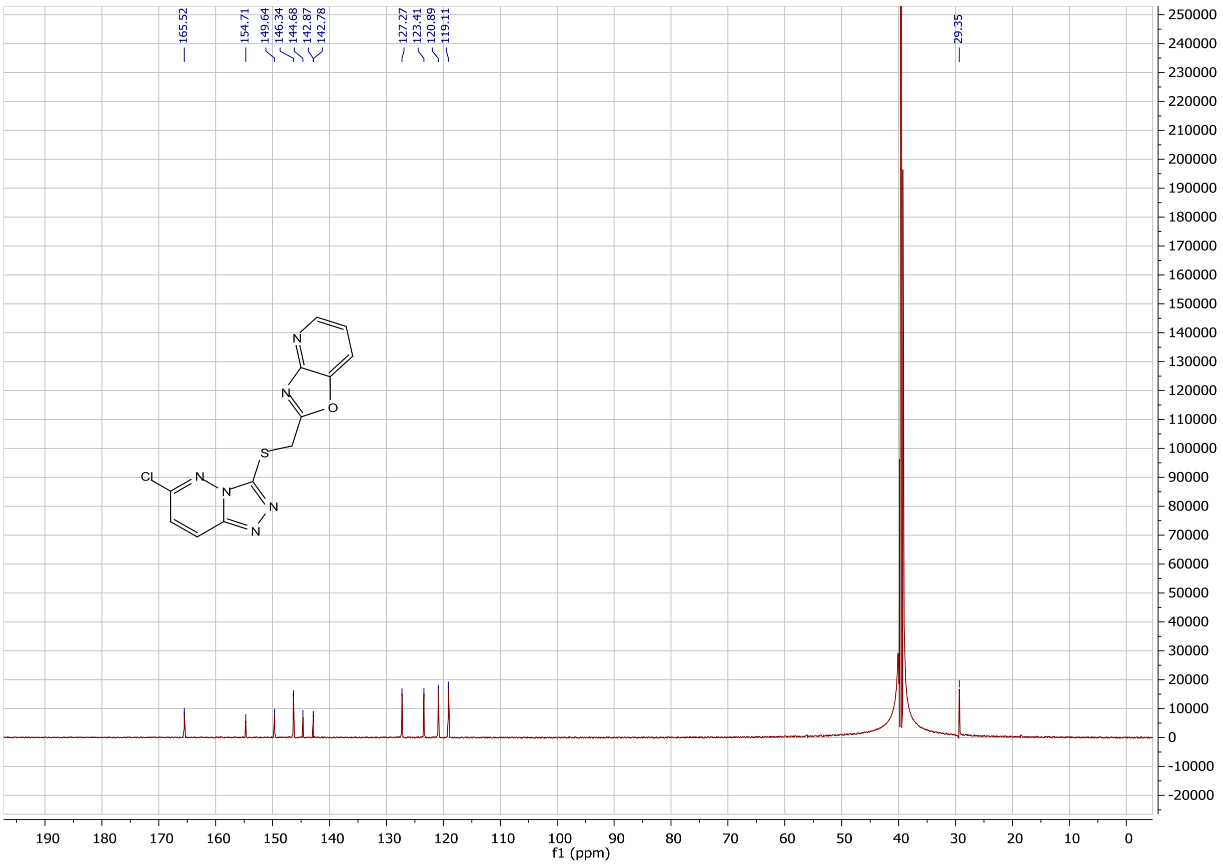

Figure S48: ^{13}C NMR for compound **42**.

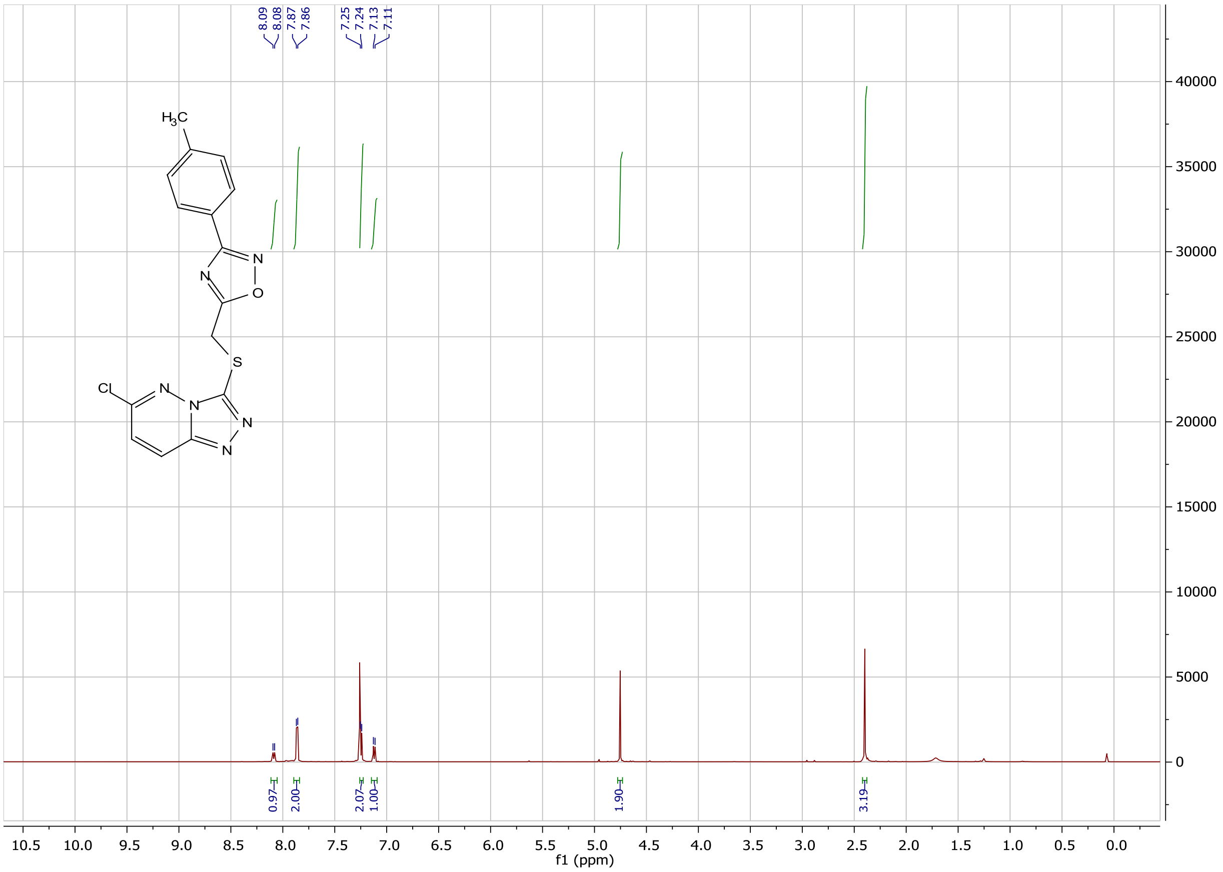

Figure S49: ^{1}H NMR for compound **43**.

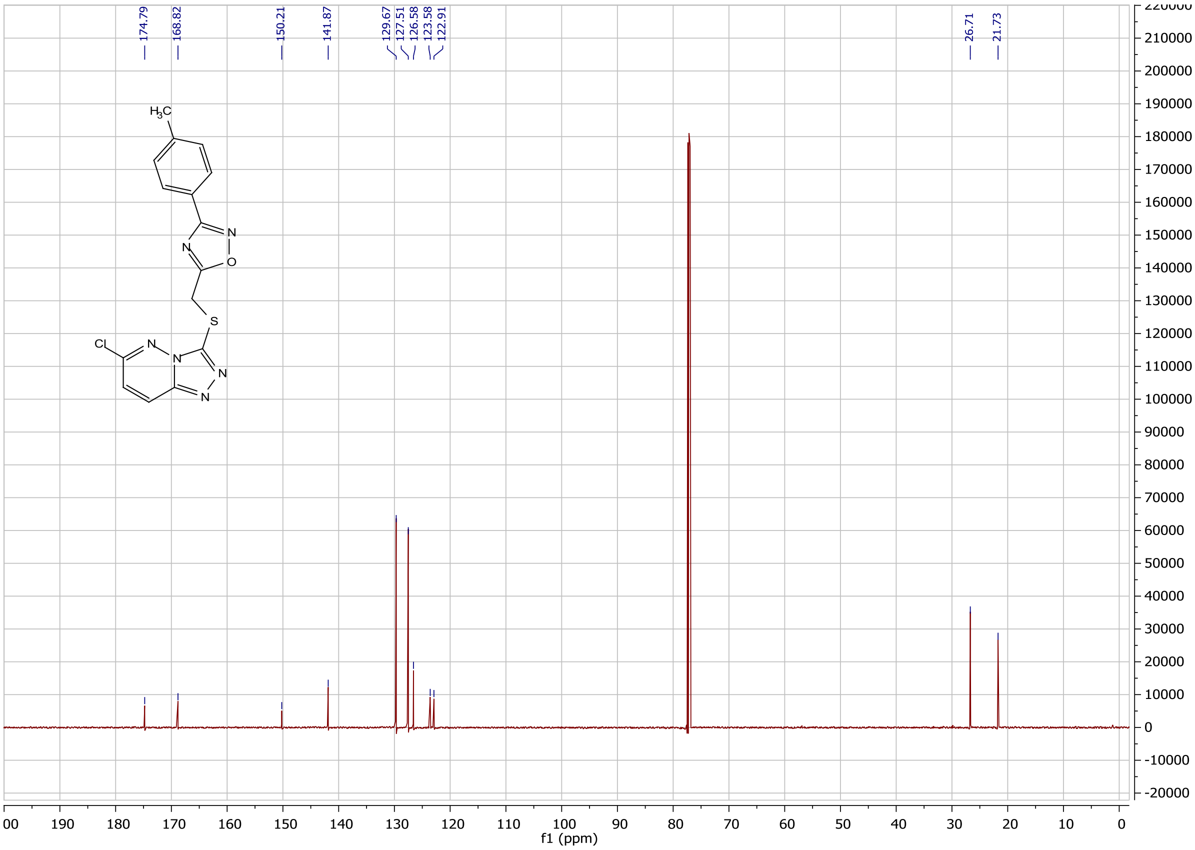

Figure S50: ^{13}C NMR for compound **43**.

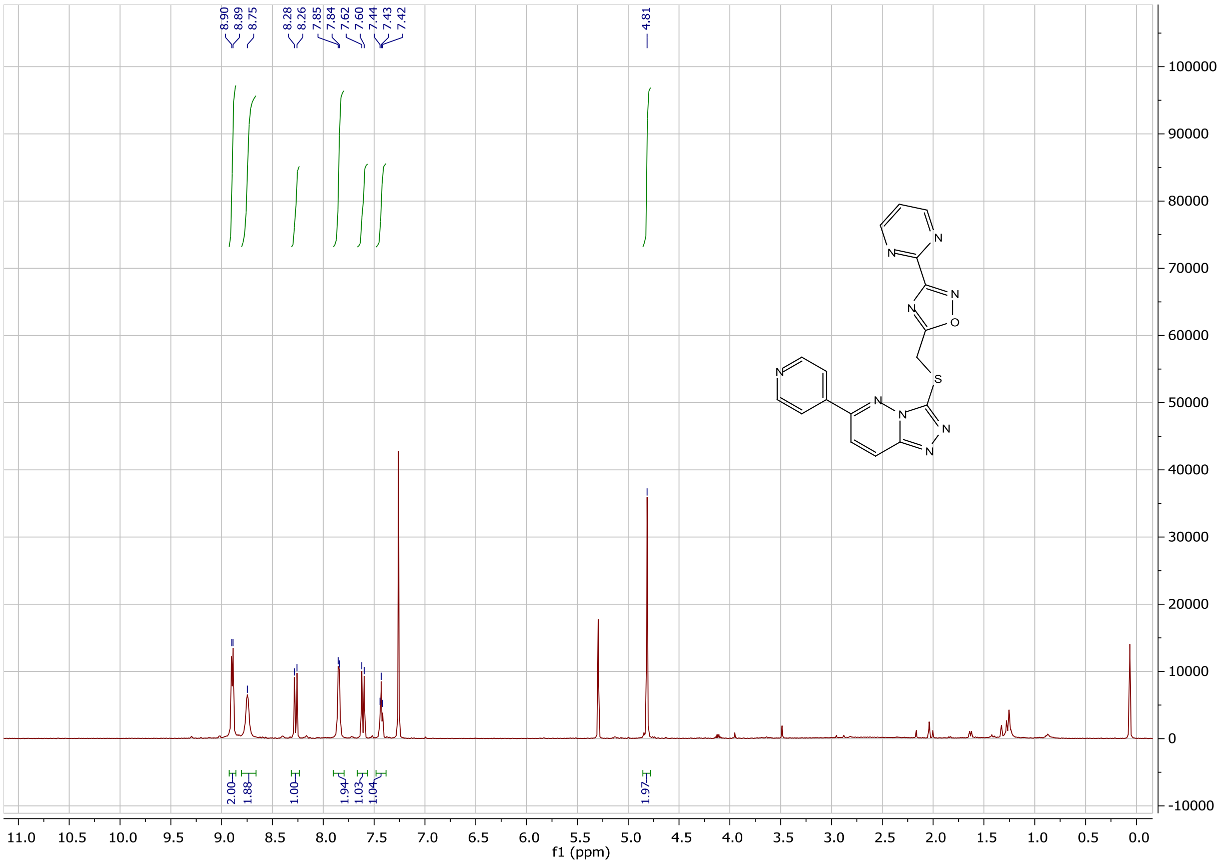

Figure S51: ^{1}H NMR for compound **44**.

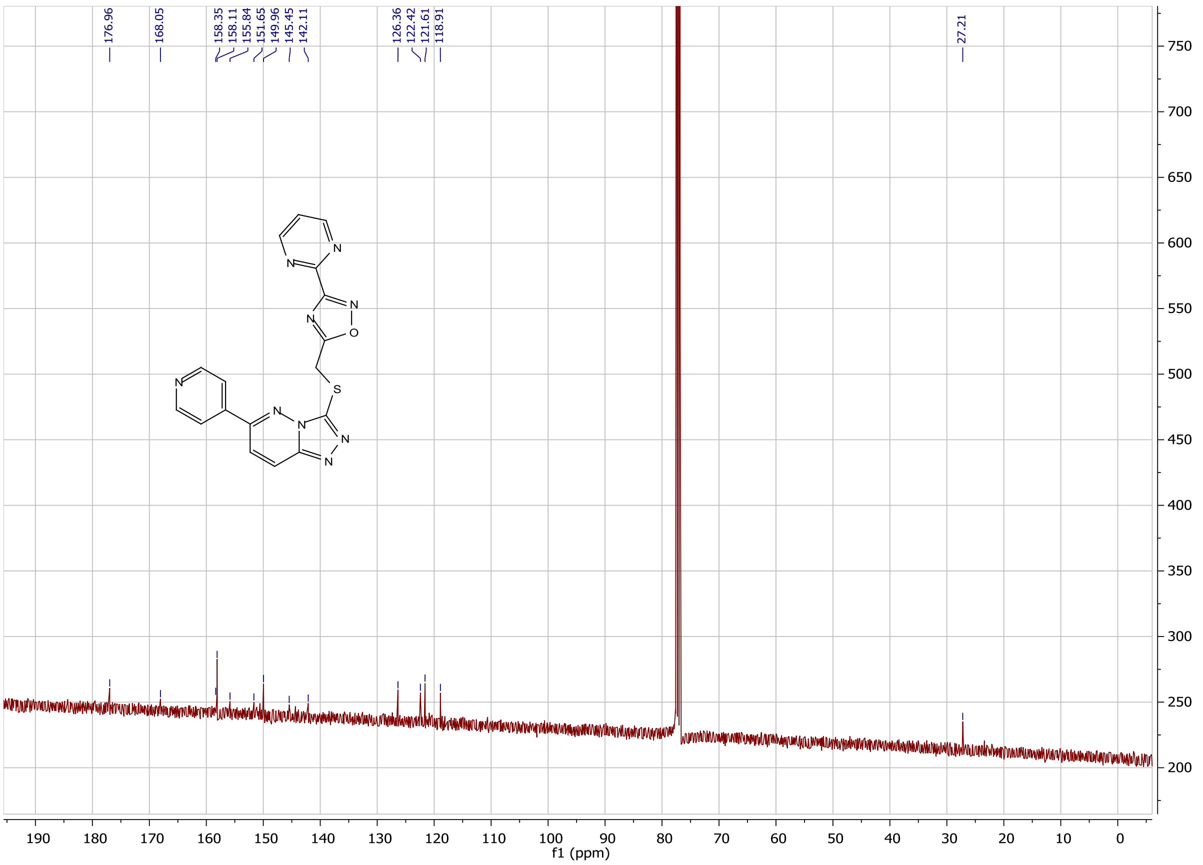

Figure S52: ^{13}C NMR for compound **44**.

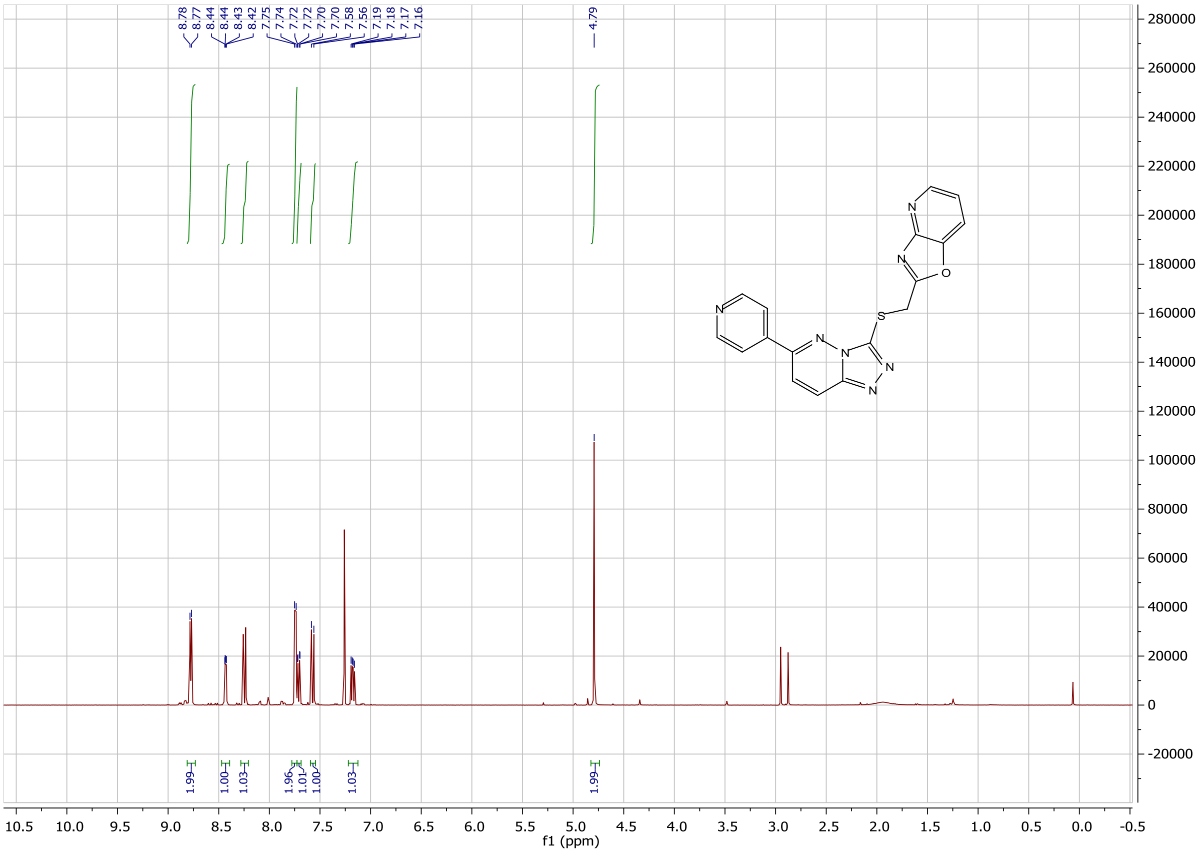

Figure S53: ^{1}H NMR for compound **45**.

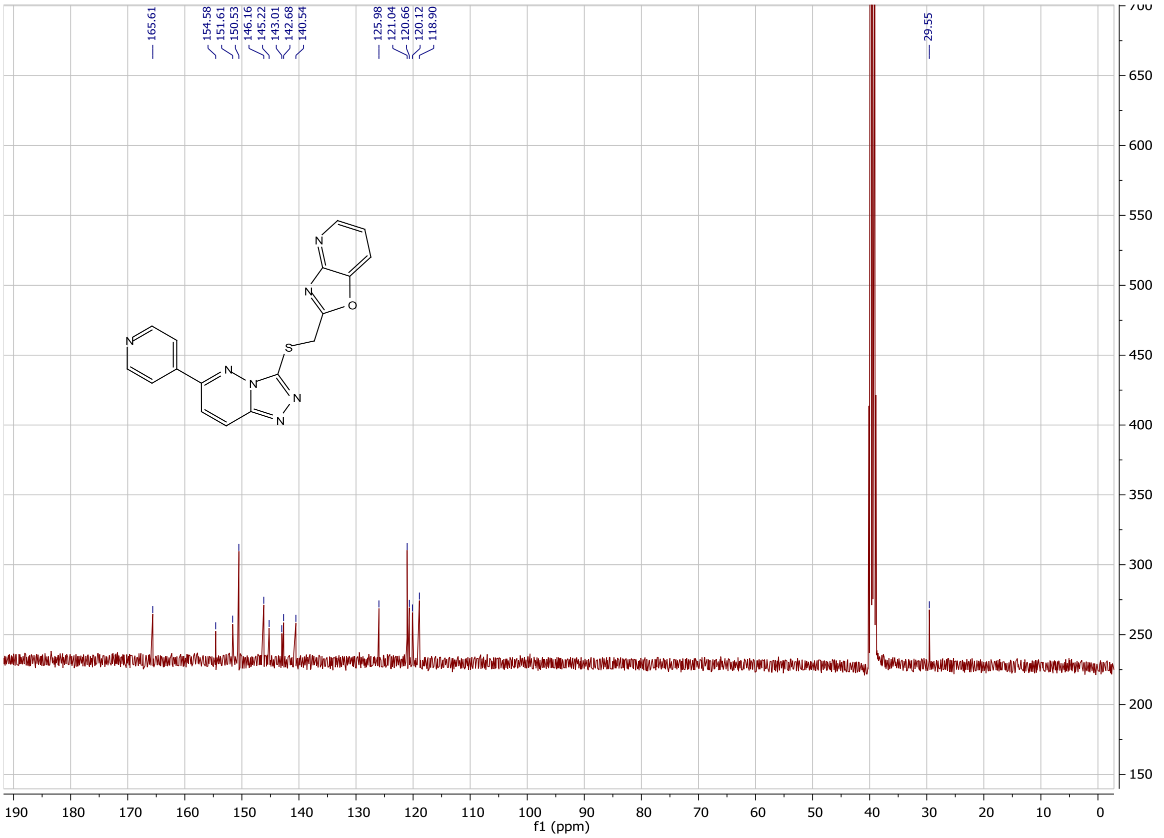

Figure S54: ^{13}C NMR for compound **45**.
